# Influence of heating temperature and time on mechanical-degradation, microstructures and corrosion performances of Teflon/granite coated aluminum alloys used for non-stick cookware

**DOI:** 10.1016/j.heliyon.2024.e34676

**Published:** 2024-07-20

**Authors:** Abdulaziz S. Alaboodi, S. Sivasankaran, Hany R. Ammar

**Affiliations:** a Department of Mechanical Engineering, College of Engineering, Qassim University, Buraydah, 51452, Saudi Arabia

**Keywords:** Non-stick cookware, Aluminum alloys, PTFE coating, Ceramic coating, Taber wear, Adhesive pull-off, Corrosion, Microstructures

## Abstract

This study explores the functional characteristics (erosion, corrosion, mechanical damage, and microstructural features) of non-stick cookware made from aluminum alloys. Typically coated with polytetrafluoroethylene (PTFE-Teflon) or ceramic for non-stick properties, we conducted a systematic investigation using corrosion, abrasion, and mechanical tests on six types of cookware from different manufacturers (Manuf-1-6). The cookware was heated at various temperatures [Room temperature (RT), 100, 175, 250, & 350 °C] and times (45 & 120 min). Tests included Taber wear, Adhesive Pull-off, hot & RT corrosion, and surface roughness measurements. Characterization involved optical microscopy, scanning electron microscope (SEM) with electron backscattered diffraction (EBSD), and x-ray diffraction (XRD). Ceramic-coated cookware from Manuf-4 demonstrated superior mechanical strength, wear, and corrosion resistance due to refined microstructures. Manuf-1's PTFE-coated cookware also performed well. Optimal results were observed when heating below 250 °C for up to 45 min. Prolonged heating and temperatures beyond 250 °C adversely affected internal structures of all cookware. Thus, it is advisable to use Al-based non-stick cookware below 250 °C for a maximum of 45 min.

## Highlights

• Non-stick cookware performances under varying conditions.
• Microstructural analysis revealed the presence of α-Al and FeAl_3_ precipitates with additional phases.
• Severe wear occurred in all cookware beyond 250 °C.
• Adhesive pull-off tests influenced the C-F atom loss in PTFE and silane loss in ceramic coatings.
• Hot corrosion tests at 175 °C revealed increased weight loss due to oxide layers and spalling.

## Graphical abstract

**Figure:**
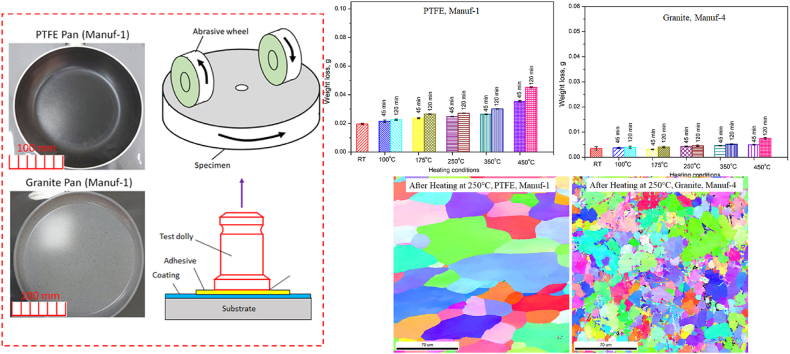

**Table:** List of Abbreviations

PTFE-Teflon: polytetrafluoroethylene	DRV: dynamic recovery
Manuf: Manufacturer	DRX: dynamic recrystallization
XRD: x-ray diffraction	CDRX: continuous dynamic recrystallization
SEM: Scanning Electron microscope	PDP: Potentiodynamic Polarization
EBSD: Electron Back-scattered Diffraction	IPFs: Inverse pole figures
RT: Room Temprature	KAM: Kernal average misorientation
OES: optical emission spectrometer	LAGBs: Low angle grain boundaries
Ra: Surface roughness	HAGBs: High angle grain boundaries
WL: Weight loss	IQ: Image quality
TWI: Taber Wear Index	

## 1 Introduction

Commercially available non-stick cookware exhibits diverse characteristics dependent on the manufacturer, offering various features for both function and aesthetic appeal [1]. Recent market trends have seen an increase in the variety of non-stick cookware, featuring different coating materials and superior surface finishes [2]. Surface engineering, particularly with polymer coatings like Polytetrafluoroethylene (PTFE or Teflon) and ceramic coatings, plays a crucial role in enhancing the performance of engineered parts, promoting health and comfort [3]. Authors have tested Al-Cu-Fe-Cr quasicrystalline and forged aluminum alloy impregnated with PTFE coating materials [4,5]. Non-stick cookware, commonly used in modern kitchens worldwide, is favored for its food non-sticking properties, ease of cleaning, and lightweight design [6]. However, concerns about the toxicity of PTFE arose in 2006. PTFE, with its auto-lubricant properties and resistance to high temperatures, has faced scrutiny, leading to the introduction of ceramic non-stick cookware by various manufacturers [7,8]. Most non-stick cookware is now made with either PTFE or ceramic-coated materials like Zirconium Nitride (ZrN), Titanium Aluminium Nitride (TiAlN), and Zirconium oxide (ZrO_2_) [5]. Commercially available cookware varies widely in characteristics across products and countries, with a focus on meeting market demands for aesthetics and features [9]. Non-stick options, usually coated on substrates like steel, aluminum, or cast iron, dominate the market, with PTFE and ceramic-based coatings being prevalent [10]. The examination of non-stick cookware behavior after mechanical damage is a current focus in the research community [1]. PTFE, or Teflon resin, is known for its smooth surface, friction-free nature, chemical resistance, high thermal stability, and electric current barrier [11]. While PTFE is inert below 260 °C, exceeding this temperature can lead to the release of harmful fumes, causing environmental pollution. Temperatures beyond 350 °C result in smoke emission and, around 400 °C, trigger pyrolysis, leading to significant thermal decomposition of PTFE [12]. Gases emitted from Teflon-coated cookware at high temperatures can pose health risks, causing respiratory problems, chest pain, and cough [13]. A study also reported a high mortality rate in poultry exposed to PTFE-coated light bulbs [14]. Table 1 illustrates commonly used cooking temperatures for different foods, while Table 2 outlines the influence of temperature on PTFE-coated cookware and its corresponding toxicity.

**Table 1:** Temperature range of different food preparation using non-stick pans/cookware [11,[15], [16], [17], [18]].

Name of food/condition/purpose	Typical required temperature, ^o^C
Baking of food items	175 to 180
Cooking of meat in an oven or grill	250 to 270
Exposing of meat in a pre-heated grill	350 to 375
The PTFE-coated pan was heated in a typical stove after 8 min	380 to 400
The PTFE-coated pan heated over the electric coil	410 to 430
Broiling of chickens in a high-end oven	750 to 815

**Table 2:** Influence of Temperature on PTFE coated non-stick cookware

Temperature, ^o^C	Effects/causes/observations [References]
160^o^C	Chicks/birds started to die [11]
200 °C	Chicken birds die when they are exposed to a light made of PTFE-coated bulbs [13]
240 °C	Teflon coating started to get off the pan [19]
280 °C	Toxic gas started to form [11]
290 °C	PTFE particles were formed at ultra-fine levels from pan [11]
360 °C	Toxic gases released from PTFE-coated pans and affected the animal carcinogen [20]
470^o^C	SiF_4_- Silica tetrafluoride formed, which is highly toxic by inhalation [21]
500^o^C	CoF_2_-Carbonyl fluoride toxic gas formed [11]
600^o^C	CF_3_COF-trifluoroacetic acid fluoride degrades to Hydrogen fluoride (HF) and Trifluoroacetic acid (TFA)-highly poisonous [21]
650^o^C	CF_4_-Carbon tetrafluoride – global warming, affecting lungs, hearts, and nervous system [21]

Heating non-stick cookware can release toxic gases, as shown in Table 2. Cookware damage occurs when exposed to sharp objects or during cleaning with iron brushes. Manufacturers recommend slow heating, but control is challenging for users. Coating removal by sharp items poses ingestion risks [15]. Ultra-fine particles form at temperatures above 290 °C, sticking to body parts (stomach and kidneys) [16]. Heating PTFE-coated pans to 486 °C generates 16 nm nanoparticles, forming highly toxic gases when mixed with fumes [22]. Damages and gases lead to the formation of perfluorooctanoic acid (PFOA), a non-biodegradable and highly toxic substance [17,18]. Studies indicate widespread PFOA presence in people, with health implications [23,24]. PFOA forms harmful gases during PTFE-coated pan use, unavoidable under normal conditions [25]. Attempts to replace PFOA with GenX still yield toxic gases [26]. Limited scientific data on the effects of GenX on PTFE-coated cookware exist. Ceramic-coated non-stick cookware, an alternative, uses nanoparticles like TiO_2_, SiO_2_, and nano-clay. However, repeated use, cleaning, and heating impact the coating, with insufficient literature on peel-off effects [27]. This study offers a comprehensive and comparative analysis of PTFE and ceramic-coated non-stick cookware made from various aluminum alloys. Unlike previous research, which often focuses on a single type of coating or aluminum alloy, our work investigates a diverse set of forged aluminum alloys (AA 1050, AA 1100, AA 1230, AA 4145, AA 1100, and AA 1145) used by different manufacturers. This study provides a detailed comparison of the mechanical, wear, adhesive, and corrosion properties of PTFE and ceramic coatings across different aluminum alloys. This comparative approach reveals the strengths and weaknesses of each type of coating under various conditions, which is not extensively covered in existing literature. The use of advanced techniques such as OES, SEM-EBSD, and XRD provides a deeper understanding of the microstructural and compositional variations between the different types of cookware. This work covered Taber wear tests, adhesive pull-off tests, and both hot and room temperature corrosion tests to assess the durability and performance of the cookware. The detailed results, including weight loss, surface roughness, and corrosion resistance, offer valuable insights into the long-term usability and reliability of the cookware under real-world cooking conditions. Further, this study systematically investigates the impact of different temperatures on the wear, adhesion, and corrosion properties of the coatings. This aspect is particularly novel as it provides practical guidelines for the safe use of non-stick cookware, emphasizing the importance of maintaining temperatures below 250 °C to minimize wear and degradation.

## 2 Methodology

### 2.1 Non-stick cookware products collection

**Fig. 1:**
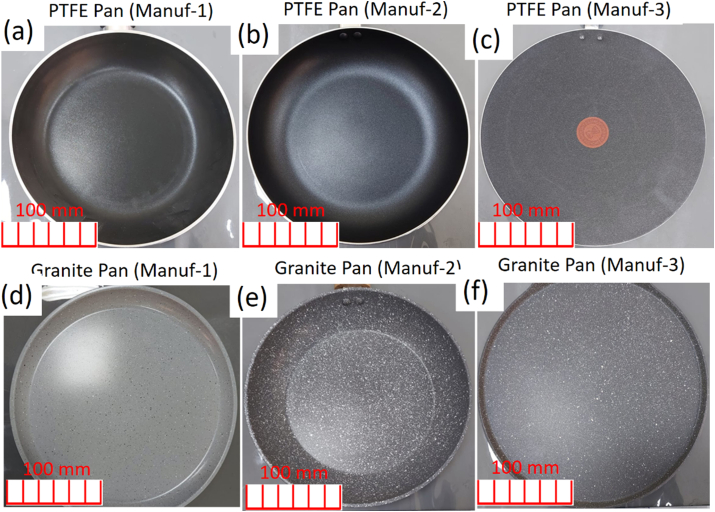
Five layers of PTFE and granite coated non-stick cookware's produced by different manufacturers: (a) PTFE Pan, Manuf-1; (b) PTFE Pan, Manuf-2; (c) PTFE Pan, Manuf-3; (d) Granite Pan. Manuf-4; (e) Granite Pan, Manuf-5; (f) Granite Pan, Manuf-Characterization of as-received non-stick cookware.

For this study, three PTFE-coated aluminum pans and three ceramic (Granite) pans from different manufacturers (Manuf 1–6) were obtained. A total of 78 freshly purchased pans, detailed in Table 3, were selected for the research. Six commonly used non-stick cookware varieties, with an average price below USD 20, were chosen based on global popularity and common usage. The manufacturer names are omitted for confidentiality. Pans with outer diameters ranging from 26 cm to 30 cm were considered. Fig. 1 displays photographs of the selected pans coated with PTFE and Granite materials from various manufacturers.

**Table 3:** Selected non-stick cookware's coated with PTFE and Granite of Al based pans made by different manufacturers

Coating type	Coating color	Sample ID
Five-layer Teflon Classic	Black	PTFE Manuf-1
Five-layer Teflon Classic	Black	PTFE Manuf-2
Five-layer Teflon Titanium	Black	PTFE Manuf-3
Five layer Granite	Gray Cream	Granite Manuf-4
Five layer Granite	Gray white	Granite Manuf-5
Five layer Granite	Gray white	Granite Manuf-6

Before assessing non-stick cookware performance, initial analyses were conducted in as-received conditions. Basic analyses, including chemical composition, x-ray diffraction, surface roughness, optical microstructures, and SEM with EBSD, were performed. The chemical composition was determined using an optical emission spectrometer (OES) by M/s Bruker AXS, Germany, with samples cut to 15 × 15 mm using a ceramic-based hand-cutter. PTFE and ceramic coating thickness over the aluminum substrate was measured using a Wintact digital ultrasonic thickness gauge, adhering to EN ISO 4287 standards. Surface roughness (Ra) was measured with a Qualitest TR 1900 surface profilometer, following ISO 468 standards.

### 2.2 Experimental plan and execution – heating temperature and time

The acquired PTFE and Granite-coated pans from various manufacturers were cleaned with ethanol to eliminate grease or dirt. To investigate heating effects, the pans underwent heating at various temperatures and times, as detailed in Table 4. The electric infrared stove/furnace, equipped with a temperature controller (refer to Appendices), heated the pans at a rate of 15 °C/min. Given cooking temperature and times typically ranging from 20 to 120 min (Table 1, Table 2, [5,11,[15], [16], [17], [18],^28^]), an average of 45 min and a maximum of 120 min were chosen for this study.

**Table 4:** Selection of operating temperatures and time for the present research work [5,11,[15], [16], [17], [18],^28^].

Heating rate, ^o^C/min	Operating temperature, ^o^C	Holding Time, minute
15 °C/min is selected based on an electric stove, furnace and according to literature	100	45, and 120
	175	45, and 120
	250	45, and 120
	350	45, and 120
	450	45, and 120

### 2.3 Taber rotary abrasion test

**Fig. 2:**
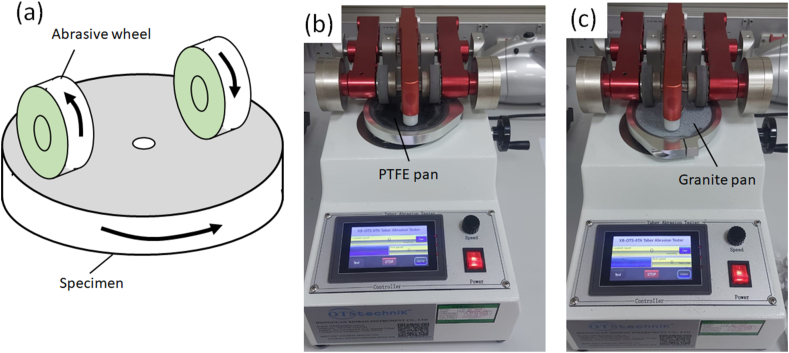
Taber rotary abrasion test: (a) schematic representing rotary abrasion test on cookware using abraded wheel; (b) Taber abrasion equipment mounted with PTFE circular pan; (c) Taber abrasion equipment mounted with ceramic circular pan.

The Taber Rotary abrasion test, commonly employed for assessing the resistance of cookware, tiles, and textiles to rubbing, scratching, scraping, and erosion, follows the ASTM D 4060 standard. This test utilizes a 100 mm diameter or square disc mounted on a turntable that rotates on a vertical axis at a specified speed. Two Taber abraded wheels with specific applied pressure are employed, using weights of 500g, 750g, or 1000g on each wheel. Fig. 2 a illustrates the schematic of the Taber rotary abrasion test as per ASTM D4060 standard. Fig. 2b and c depict the Taber abrasion equipment with a PTFE-coated pan and a Granite-coated pan, respectively.

Post-heating, a 105 mm diameter circular disc was laser-cut (refer to Appendices for details and photos). To assess abrasion resistance, weight loss (WL) and the Taber wear index (TWI) were employed. The TWI represents the wear rate per 1000 cycles (Eq. (1)) [29].

(1) $$Taber\;wear\;index\;\left(TWI\right)=\frac{\left(W_{before}-W_{after}\right)}{N}\times 1000$$

where, *W*_*before*_ is the pre-test weight, and *W*_*after*_ is the post-test weight, N is the number of cycles (72 rpm and 500 cycles were used in this study). CS-10 abrading wheels with a 1 kg counterweight were employed for three tests per sample, totaling 1500 cycles.

### 2.4 Pull-off adhesion test

**Fig. 3:**
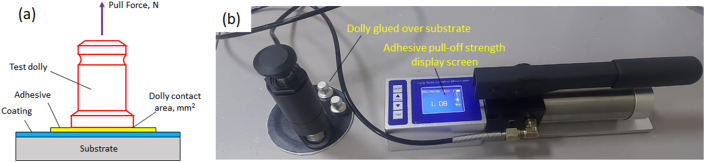
(a) Schematic diagram representing the Pull-Off adhesive test; (b) Pull-Off adhesive tester used during the experiment.

The best method for assessing protective coating strength is the portable pull-off adhesion test, a uniaxial test following ASTM: D4541 standards. The process involves cleaning the sample surface, applying strong glue to a designated area, and pressing a 20 mm diameter dolly onto the substrate. After curing (at least one day), a portable pull-off adhesion tester applies a perpendicular force, removing the dolly from the coated materials. The force at which the coating fails per dolly contact area provides the adhesive strength. Fig. 3a illustrates the schematic diagram of the pull-off adhesive test, and Fig. 3b displays the pull-off tester used. Further details and sample photos are available in the Appendices.

### 2.5 Surface roughness measurement on damaged surfaces

**Fig. 4:**
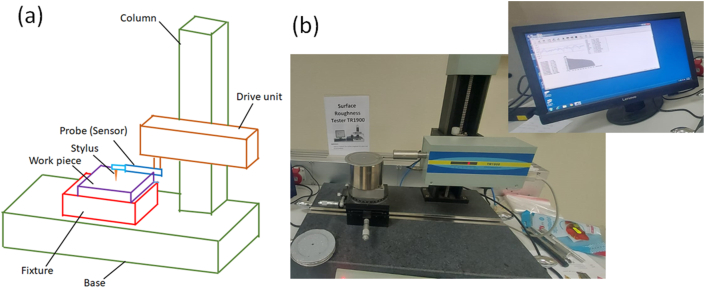
(a) Schematic of stylus-type surface roughness measuring instrument; (b) Photograph of TR 1900 surface roughness tester used in this work.

Surface roughness post-heating and damage from the abrasion test was assessed using a TR-1900 surface profile meter in adherence to ISO 468 standards. Fig. 4a illustrates the stylus-type surface profilometer schematic, encompassing a base, column, drive unit along x-, y-, and z-axes, fixture, probe (sensor), stylus, and workpiece. Fig. 4b displays the TR 1900 surface roughness testing equipment used. Prior to measuring surface roughness after the Taber abrasion test, samples were cleaned with ethanol for debris-free surfaces and placed over the fixture. The probe, connected to a drive system with adjustable knobs, ensured contact with the stylus on the sample. Software interpreted results, recording at least ten readings in each damaged sample, with the average reported.

### 2.6 Hot oxidation and corrosion test

Hot oxidation and corrosion resistance are crucial for cookware exposed to varying temperatures in an open atmosphere [[30], [31], [32]]. Using 15 × 15 mm^2^ samples from as-received non-stick cookware, hot oxidation and corrosion tests were conducted in a tube furnace under air at temperatures of 100, 175, 250, and 350 °C for cycles ranging from 0 to 25 h, following ASTM G111-21a standards. A hostile environment was created with a mix of vanadium oxide (V_2_O_5_, 75 %), sodium sulfide (Na_2_SO_4_, 20 %), and sodium chloride (NaCl, 5 %). The salts, mixed in a ratio of 0.75:0.2:0.05 and dissolved in distilled water, were deposited on the sample surface. Weight differences were measured every 5 h during the 25 h cycles using an electronic balance with 0.001 mg accuracy. For corrosion resistance, a RT corrosion test, as per ASTM G5 standards, was also performed on 10 × 12.5 × 6 mm samples cut from the pans. Samples were mechanically ground with SiC papers (grits ranging from 400 to 1200), polished with 1 μm Al_2_O_3_ abrasive paste, cleaned with ethanol, and dried. The polished sample cross-sections were exposed to NaCl liquid. Room temperature corrosion resistance was evaluated using an electrochemical workstation (WEIS500), employing a three-electrode cell with a saturated calomel electrode (SCE) as the reference, platinum foil as the auxiliary, and the sample as the working electrode. Three trials per pan were conducted, and an average value was used for analysis.

## 3 Results and discussion

### 3.1 Examination of chemical composition and coating thickness of as-received non-stick cookware

The chemical composition of as-received non-stick cookware was analyzed using OES. Six trials for each pan were conducted, and Table 5 displays the average values. The observed elements (Si, Mn, P, Cr, Ni, Ti, Mg, Pb, Sn, Sr, Zr, Al, Cu, Zn, Fe, V, Co, Ca, Sb, B, Cd, Ag, Ga) were compared with ASM International Aluminum and Aluminum Alloys handbook [33]. The non-stick cookware was identified as forged aluminum alloys—AA 1050, AA 1100, AA 1230, AA 4145, AA 1100, and AA 1145 series for Manuf-1 through Manuf-6, respectively. Physical and mechanical properties from Table 6 were sourced from various handbooks [[33], [34], [35], [36]]. While density values were similar, melting point and tensile strength varied due to differing chemical compositions. For example, Manuf-4 exhibited a tensile strength of approximately 480 MPa, attributed to higher Si and Cu content (Table 5). Coating thickness was measured with an ultrasonic gauge. The velocity of sound for aluminum, PTFE, and Granite was set at 6420 m/s, 1400 m/s, and 5950 m/s, respectively. After applying coupling gel, thickness was measured in at least ten places. Average coating thicknesses was 275 ± 2.8 μm, 235 ± 4.8 μm, 345 ± 3.9 μm, 265 ± 5.6 μm, 125 ± 3.4 μm, and 205 ± 2.5 μm for Manuf-1 through Manuf-6, respectively. PTFE-coated cookware features carbon-fluorine (C-F) atoms over the Al alloy substrate, while ceramic-coated cookware includes silane compounds over the Al alloy substrate. Coating thickness, measured ultrasonically, was consistent across pans, except for Manuf-3 and Manuf-5 non-stick cookware.

**Table 5:** Chemical composition of as-received non-stick cookware tested by OES (weight %)

Coating type, Manufacturer	Si	Mn	P	Cr	Ni	Ti	Mg	Pb	Sn	Sr	Zr	Al
PTFE, Manuf-1	0.0468	0.0113	0.0038	0.0019	0.0005	0.0246	0.0048	0.0065	0.0005	0.0010	0.0002	99.5333
PTFE, Manuf-2	0.13	0.63	0.00	0.01	0.00	0.02	0.00	0.01	0.00	0.00	0.00	98.60
PTFE, Manuf-3	0.15	0.00	0.00	0.00	0.00	0.02	0.00	0.01	0.00	0.00	0.00	99.30
Granite, Manuf-4	10.60	0.23	0.00	0.03	0.11	0.04	0.21	0.05	0.02	0.00	0.01	84.37
Granite, Manuf-5	0.16	0.69	0.00	0.01	0.00	0.02	0.00	0.01	0.00	0.00	0.00	98.50
Granite, Manuf-6	0.11	0.02	0.00	0.02	0.00	0.01	0.01	0.01	0.00	0.00	0.00	99.40

Coating type, Manufacturer	Cu	Zn	Fe	V	Co	Ca	Sb	B	Cd	Ag	Ga	Identified Al alloy series
PTFE, Manuf-1	0.0010	0.0033	0.3175	0.0101	0.0005	0.0006	0.0034	0.00	0.00	0.00	0.01	AA 1050
PTFE, Manuf-2	0.00	0.01	0.51	0.03	0.00	0.00	0.00	0.00	0.00	0.00	0.01	AA 1100
PTFE, Manuf-3	0.00	0.00	0.48	0.01	0.00	0.00	0.00	0.00	0.00	0.00	0.01	AA 1230
Granite, Manuf-4	2.59	0.89	0.82	0.01	0.00	0.00	0.00	0.00	0.00	0.00	0.01	AA 4145
Granite, Manuf-5	0.00	0.01	0.57	0.02	0.00	0.00	0.00	0.00	0.00	0.00	0.01	AA 1100
Granite, Manuf-6	0.01	0.07	0.33	0.01	0.00	0.00	0.00	0.00	0.00	0.00	0.01	AA 1145

**Table 6:** Physical, electrical, and mechanical properties of as-received non-stick cookwares [[33], [34], [35], [36]].

Coating type-Manufacturer	Name of Aluminium alloy of substrate non-stick cookware	Property	Value
PTFE Manuf-1	AA 1050	Density	2.71 g/cm^3^
		Melting point	650 °C
		Thermal expansion	24 × 10^−6^/K
		Modulus of elasticity	71 GPa
		Thermal conductivity	222 W/mK
		Electrical resistivity	0.0282 × 10^−6^ Ωm
		Tensile strength	125 MPa
		Brinell hardness strength	34 HB
		Elongation	12 % and above
Granite Manuf-4	AA 4145	Density	2.74 g/cm^3^
		Melting point	585 °C
		Thermal expansion	23.7 × 10^−6^/K
		Thermal conductivity	138 W/mK
		Tensile strength	480 MPa
		Elongation	Upto 13 %
PTFE Manuf-2 & Granite Manuf-5	AA 1100	Density	2.71 g/cm^3^
		Melting point	657 °C
		Thermal expansion	23.6 × 10^−6^/K
		Modulus of elasticity	75 GPa
		Thermal conductivity	218 W/mK
		Electrical resistivity	0.030 × 10^−6^ Ωm
		Tensile strength	110 MPa
		Brinell hardness strength	28 HB
		Elongation	12 % and above
PTFE Manuf-3	AA 1230	Density	2.70 g/cm^3^
		Melting point	640 °C
		Thermal expansion	23 × 10^−6^/K
		Modulus of elasticity	69 GPa
		Thermal conductivity	230 W/mK
		Tensile strength	130 MPa
Granite Manuf-6	AA 1145	Density	2.70 g/cm^3^
		Melting point	647 °C
		Modulus of elasticity	75 GPa
		Thermal conductivity	227 W/mK
		Electrical resistivity	0.028 × 10^−6^ Ωm
		Tensile strength	130 MPa
		Brinell hardness strength	21 B

### 3.2 Optical microstructures and SEM with EBSD analyses on as-received non-stick cookware

**Fig. 5:**
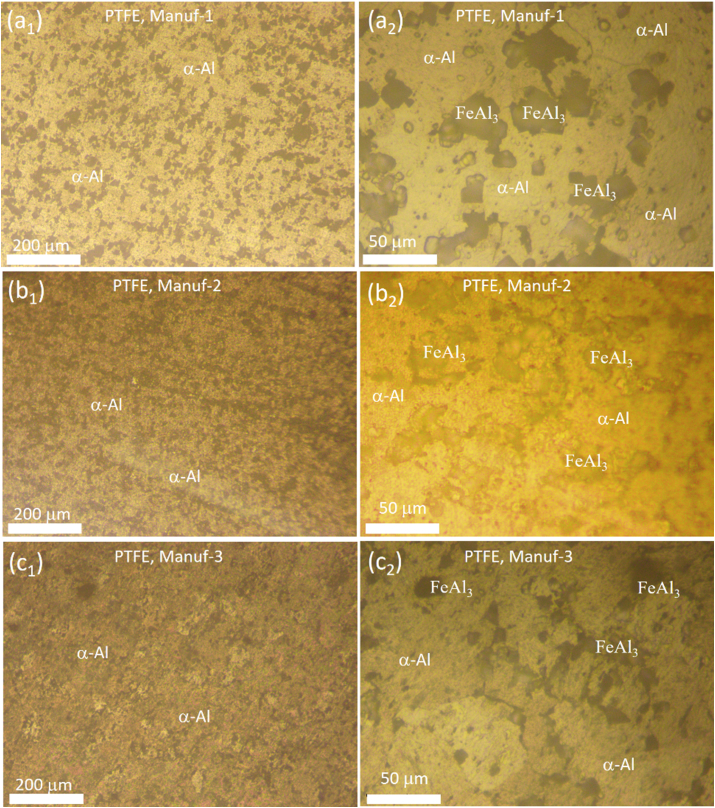

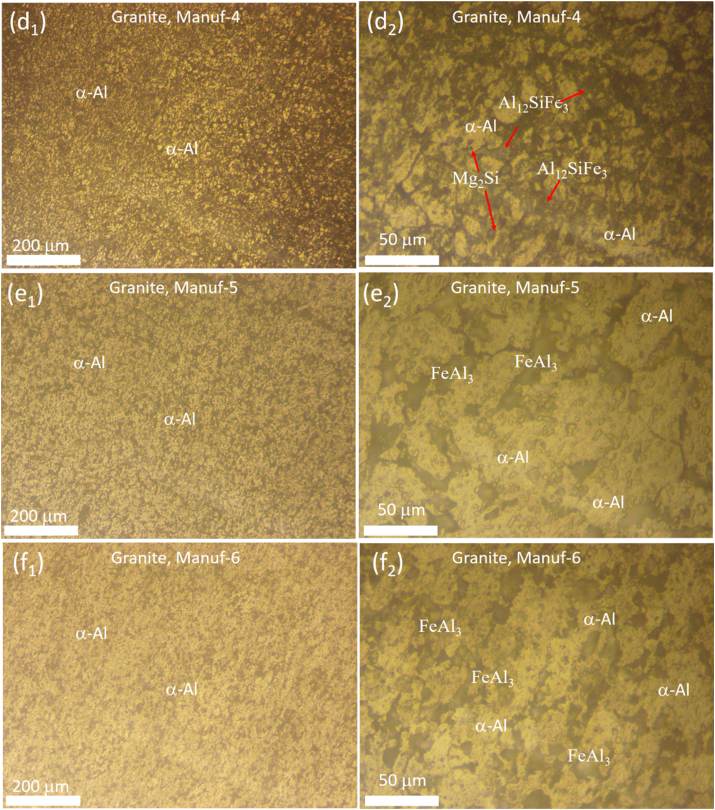
Optical microstructures of as-received non-stick cookware made of different manufacturers: (a1-a2) AA1050 PTFE, Manuf-1; (b1-b2) AA1100 PTFE, Manuf-2; (c1-c2) AA1230 PTFE, Manuf-3; (d1-d2) AA4145 Granite, Manuf-4; (e1-e2) AA1100 Granite, Manuf-5; (f1-f2) AA1145 Granite, Manuf-6.

**Fig. 6:**
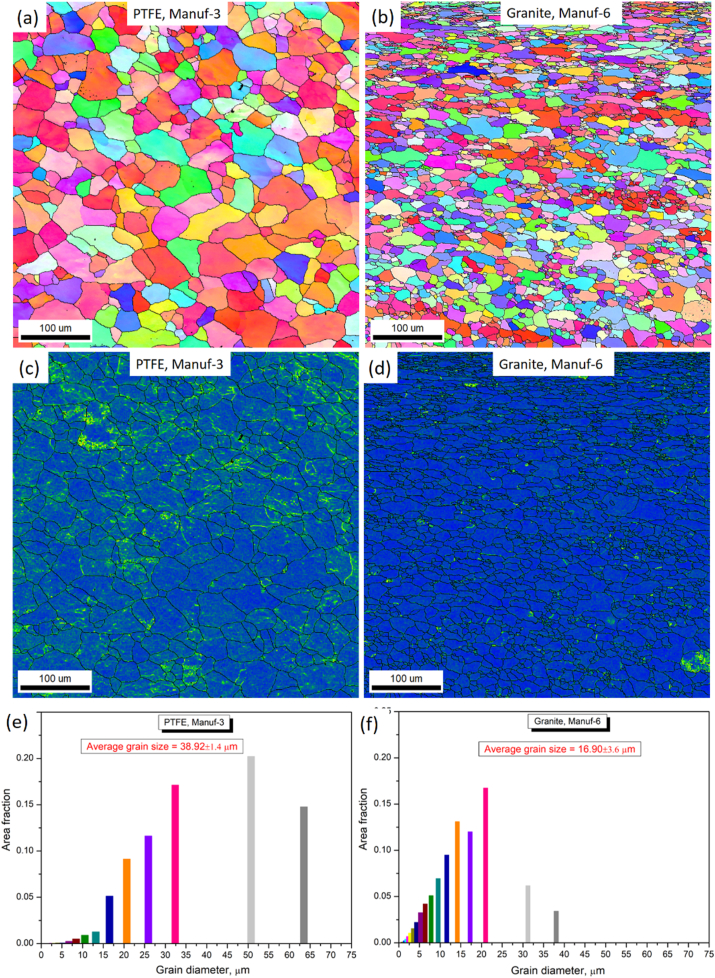
HRSEM-EBSD analyses of as-received non-stick cookware's of PTFE, Manuf-3, and, Granite, Manuf-6: (a) & (b) Grain colored map; (c) & (d) KAM map; (e) & (f) grain size distribution with area fraction.

**Fig. 7:**
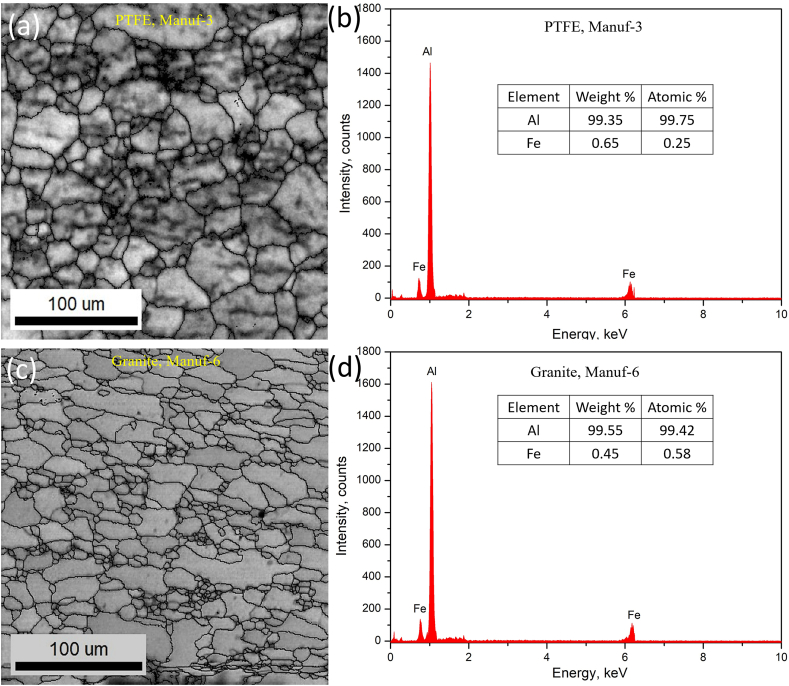
SEM-EBSD IQ (image quality) map of as-received cookware of: (a) PTFE, Manuf-3, and (c) Granite, Manuf-6; corresponding EDAX spectra of: (c) PTFE, Manuf-3, and (d) Granite, Manuf-6.

Optical microstructural analyses were performed to scrutinize the grain morphology and structure of as-received non-stick cookware. Fig. 5a_1_-a_2_, Fig. 5b_1_-b_2_, Fig. 5c_1_-c_2_, Fig. 5d_1_-d_2_, Fig. 5e_1_-e_2_, and Fig. 5f_1_-f_2_ illustrate the optical microstructures of PTFE and Granite non-stick cookware from various manufacturers. The PTFE of Manuf-1, Manuf-2, Manuf-3, and Granite of Manuf-5, and Granite of Manuf-6 exhibited equiaxed α-Al matrix grains and FeAl_3_ intermetallic phases. However, the Granite of Manuf-4 displayed an additional Al_12_SiFe_3_ intermetallic phase due to its AA 4145 series composition. Varied grain structures were observed, with fine grains in Manuf-4, Manuf-5, and Manuf-2, coarse grains in Manuf-3 and Manuf-1, and intermediate grains in Manuf-6. HRSEM with EBSD analyses focused on PTFE pan (Manuf-3) and Granite pan (Manuf-6). EBSD grain-colored maps (selected samples, Fig. 6 a& b) revealed a coarse grain size in PTFE pan of Manuf-3 and a fine grain structure in Granite pan of Manuf-6. Kernel average misorientation (KAM, Fig. 6 c & d) dislocation maps indicated more dislocation lines in PTFE pan of Manuf-3, signifying increased surface defects due to forging. Fewer dislocation lines were observed in Granite pan of Manuf-6, suggesting fewer structural defects. Grain size distribution from HRSEM-EBSD analyses revealed an average grain size of 38.92 μm for Manuf-3 (PTFE) and 16.9 μm for Manuf-6 (Granite), indicating a fine grain structure consistent with the optical microstructure. Further, SEM-EBSD image quality (IQ) maps and EDAX spectra were also carried out for the PTFE pan of Manuf-3 and the Granite pan of Manuf-6 non-stick cookware in as-received condition, as illustrated in Fig. 7. The EDAX spectra results, along with the chemical composition, matched the composition checked by OES, as given in Table 5.

### 3.3 XRD, optical microstructure, and SEM-EBSD analyses on cookware after heating at different temperatures

**Fig. 8:**
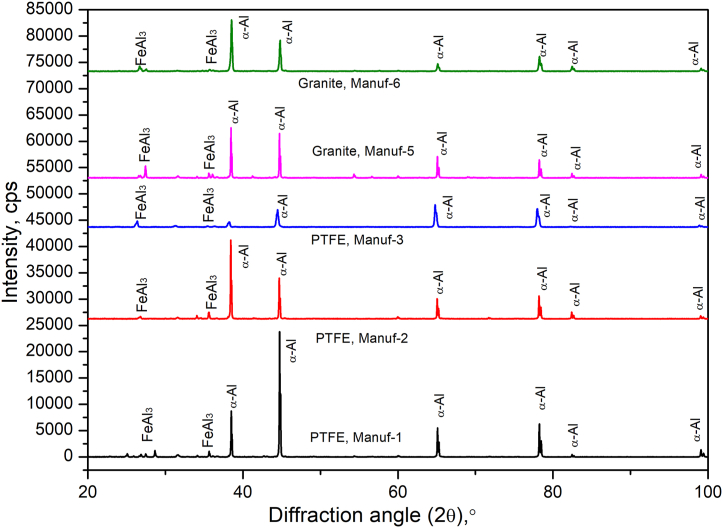
X-ray diffraction peak profile of as-received non-stick cookware from different manufacturers.

X-ray diffraction (XRD) peak profile tests were conducted on as-received non-stick cookware to assess the presence of phases in the manufactured condition. Fig. 8 displays the XRD peak profile of the pans, revealing α-Al and FeAl_3_ phases consistent with the processing of corresponding aluminum alloys. These phases align precisely with those observed in the optical microstructure (Fig. 5). No additional peaks were noted, indicating high purity in the as-received pans. The average surface roughness, measured at a minimum of 15 locations in each as-received cookware using the Qualitest profile instrument, ranged from 0.00567 μm to 0.01589 μm for Manuf-1 to Manuf-6. These results affirm that all pans exhibited good surface finishes in their manufactured conditions.

**Fig. 9:**
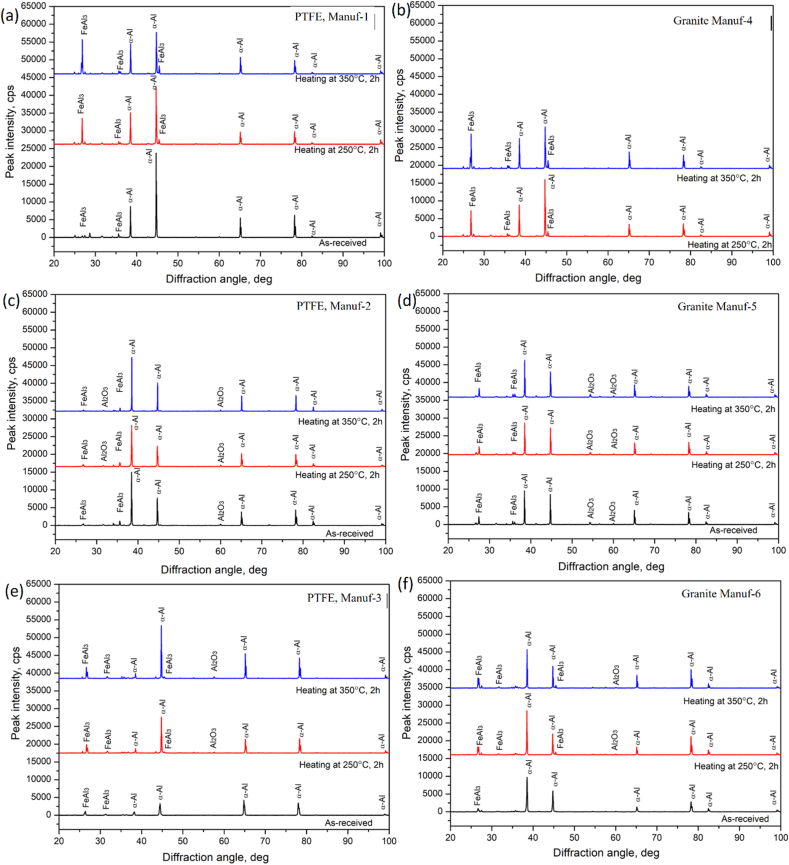
XRD results of cookware heated at different temperatures (as-received, 250 °C, & 350 °C for 2 h supplied by different suppliers: (a) PTFE Manuf-1; (b) Granite Manuf-4; (c)PTFE Manuf-2; (d) Granite Manuf-5; (e) PTFE Manuf-3; (f) Granite Manuf-6.

**Fig. 10:**
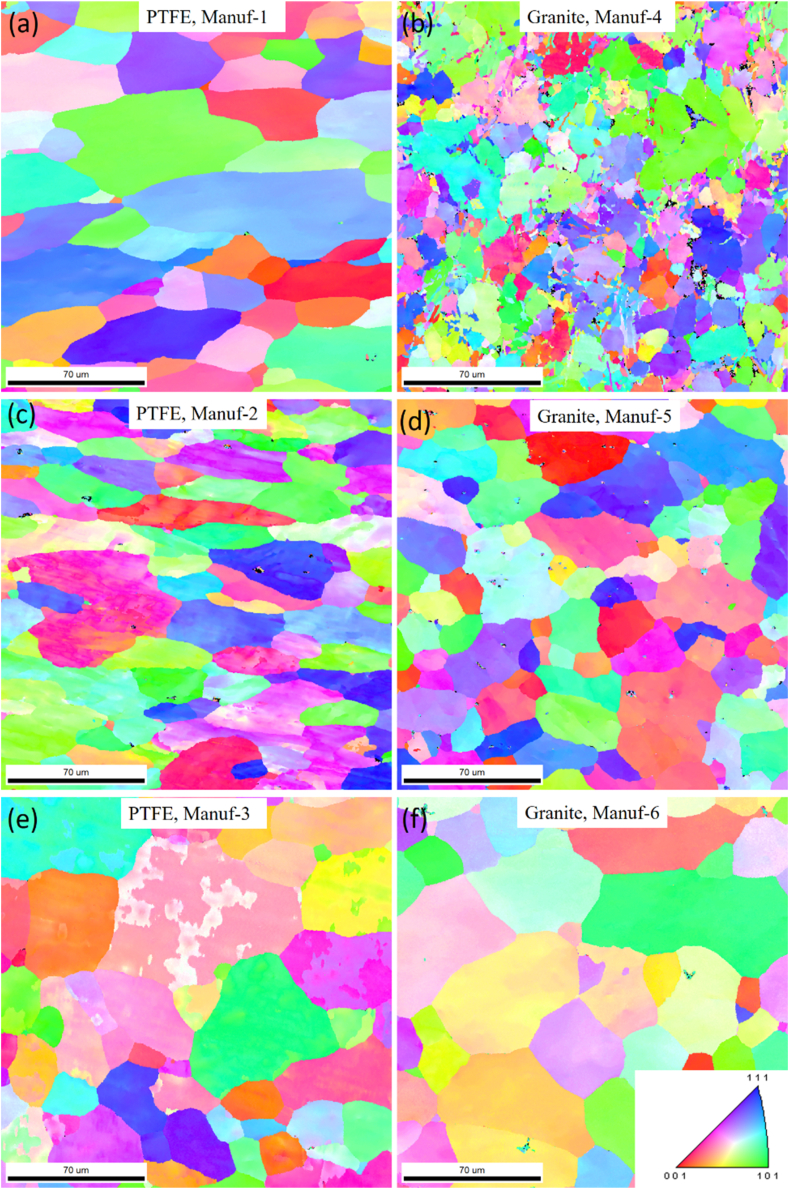
SEM-EBSD IPF results of cookware heated at 250 °C for 2 h produced by different suppliers: (a) PTFE Manuf-1; (b) Granite Manuf-4; (c)PTFE Manuf-2; (d) Granite Manuf-5; (e) PTFE Manuf-3; (f) Granite Manuf-6.

**Fig. 11:**
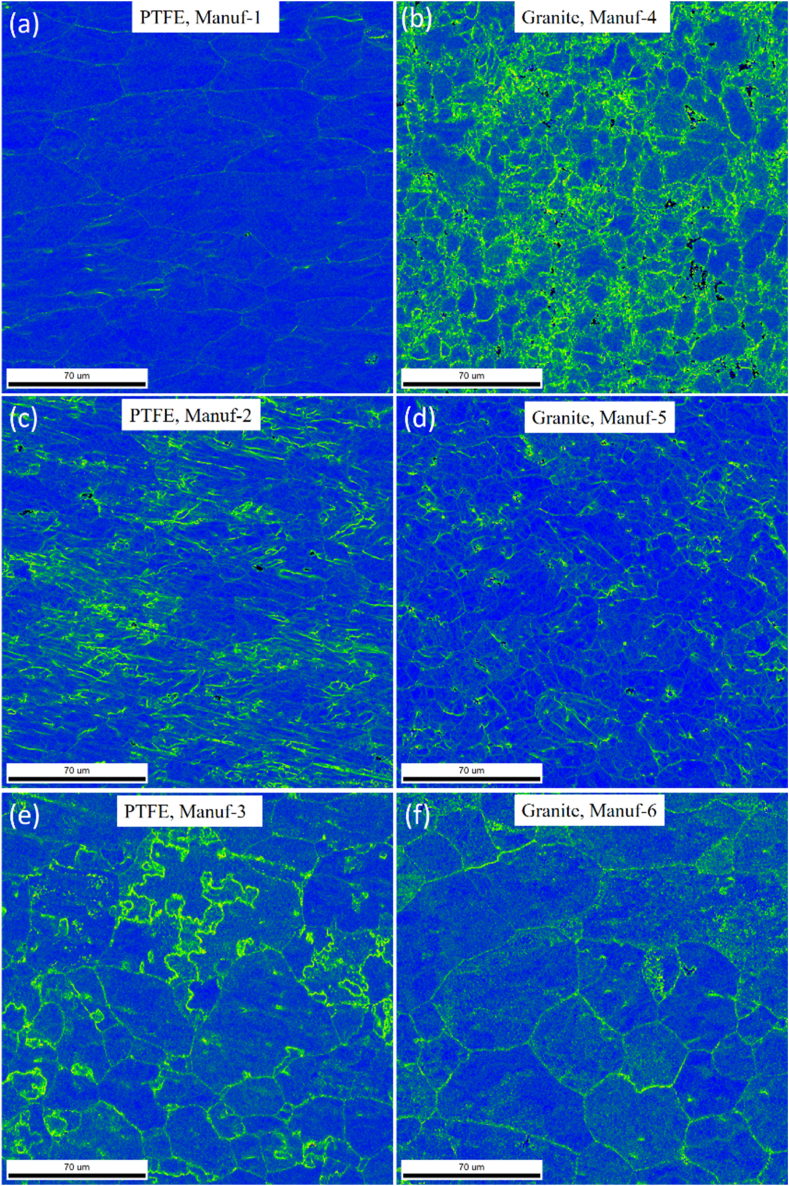
SEM-EBSD local misorientation map results of cookware heated at 250 °C for 2 h produced by different suppliers: (a) PTFE Manuf-1; (b) Granite Manuf-4; (c)PTFE Manuf-2; (d) Granite Manuf-5; (e) PTFE Manuf-3; (f) Granite Manuf-6.

**Fig. 12:**
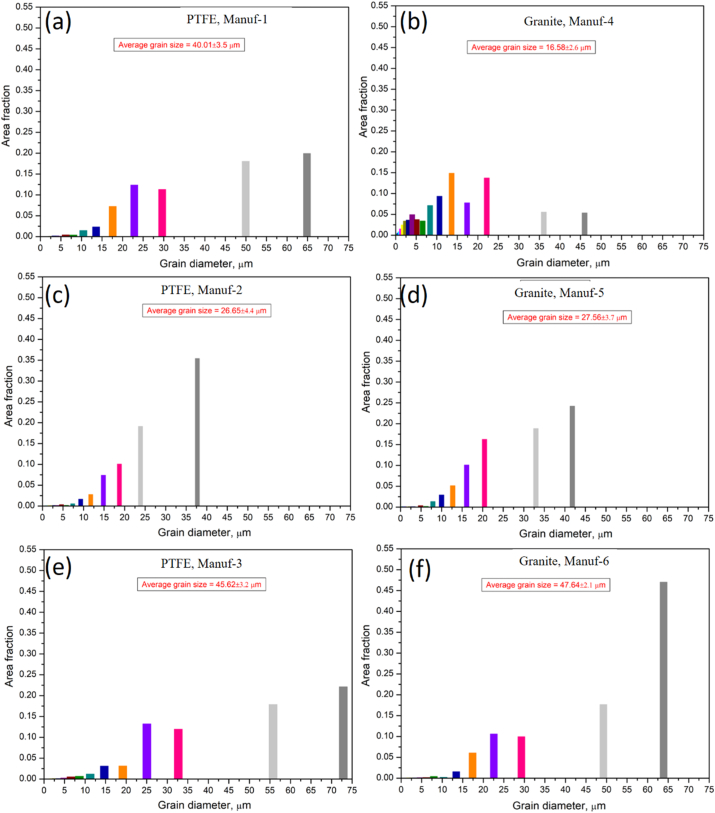
Grain size distribution vs. area fraction obtained from SEM-EBSD analyses of cookware heated at 250 °C for 2 h produced by different suppliers: (a) PTFE Manuf-1; (b) Granite Manuf-4; (c)PTFE Manuf-2; (d) Granite Manuf-5; (e) PTFE Manuf-3; (f) Granite Manuf-6.

**Fig. 13:**
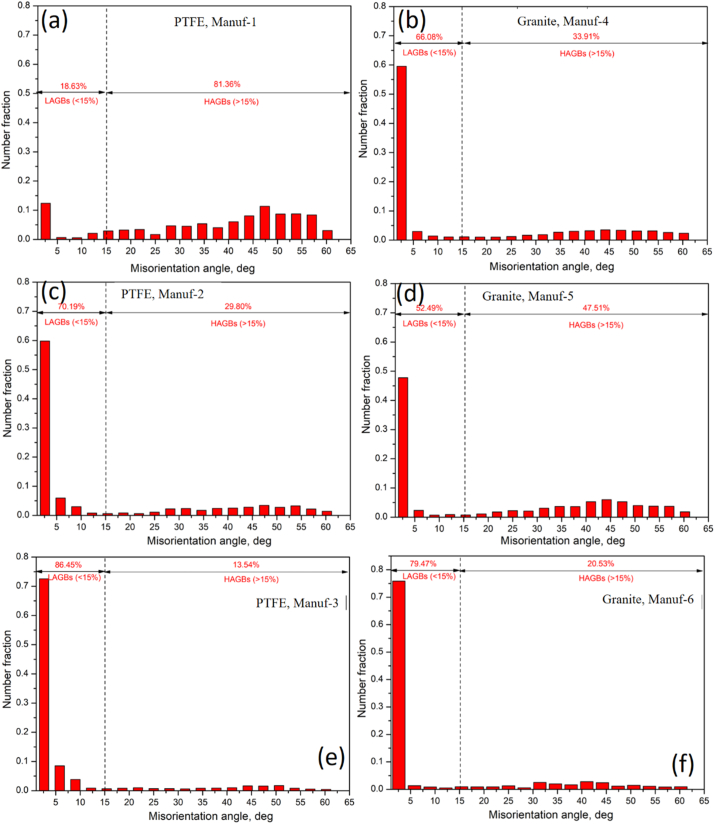
Distribution of misorientation angle vs. number fraction obtained from SEM-EBSD analyses of cookware heated at 250 °C for 2 h produced by different suppliers: (a) PTFE Manuf-1; (b) Granite Manuf-4; (c)PTFE Manuf-2; (d) Granite Manuf-5; (e) PTFE Manuf-3; (f) Granite Manuf-6.

After heating pans, XRD studies at 250 °C & 350 °C for 2 h (Fig. 9) revealed varying peak profiles, indicating structural changes. In all cookware (except PTFE Manuf-2, Fig. 9c), major α-Al and FeAl_3_ peaks were observed, with increased FeAl_3_ peak height at higher temperatures. Granite Manuf-4 showed the most prominent FeAl_3_ peaks (Fig. 9b), suggesting enhanced strength due to FeAl_3_ precipitates. PTFE Manuf-3 (Fig. 9e) and Granite Manuf-6 (Fig. 9f) also exhibited FeAl_3_ peaks, but with lower heights than PTFE Manuf-1 and Granite Manuf-4 ceramic cookware. SEM-EBSD inverse pole Figures (IPF) at 250 °C for 2 h (Fig. 10) revealed structural changes. PTFE Manuf-1 (Fig. 10a) showed little elongated α-Al grains, indicating potential strength loss. Granite Manuf-4 (Fig. 10b) displayed fine equiaxed grains due to more FeAl_3_, enhancing strength. PTFE Manuf-2 (Fig. 10c) exhibited elongated α-Al grains, indicating severe grain growth and lattice distortion. Granite Manuf-5, PTFE Manuf-3, and Granite Manuf-6 (Fig. 10d, e & f) showed regular equiaxed grains, larger than as-received conditions. Structural changes at 250 °C affect cookware performance. The structural changes in terms of the presence of dislocation density can be examined using an SEM-EBSD misorientation map. If any sample possesses more contrast in the green color mean, the corresponding sample would have more dislocation density. Fig. 11 presents SEM-EBSD misorientation maps of cookware after heating at 250 °C for 2 h. Granite Manuf-4 displayed high dislocation density contrast (Fig. 11b), indicating FeAl_3_ precipitates and fine grains, while PTFE Manuf-3 showed the next high contrast (Fig. 11e). PTFE Manuf-2 had an average dislocation density (Fig. 11c), Granite Manuf-6 had little contrast (Fig. 11f), and PTFE Manuf-1 had very little (Fig. 11a). Fig. 12 illustrates the variation of grain size with area fraction. Average grain size of α-Al matrix after heating at 250 °C for 2 h was 40.01 ± 3.5, 16.58 ± 2.6, 26.65 ± 4.4, 27.56 ± 1.7, 45.62 ± 1.2, and 47.64 ± 2.1 μm for PTFE Manuf-1, Granite Manuf-4, PTFE Manuf-2, Granite Manuf-5, PTFE Manuf-3, and Granite Manuf-6, respectively. Granite Manuf-4 exhibited fine grains due to more FeAl_3_ precipitates and dislocation density. Fig. 13 displays grain misorientation angle distribution. Two categories of grain boundaries (GBs) are low-angle (LAGBs, <15°) and high-angle (HAGBs, >15°), influencing cookware performance. The observed results provide insights into the structural changes and potential performance impact during heating.

**Fig. 14:**
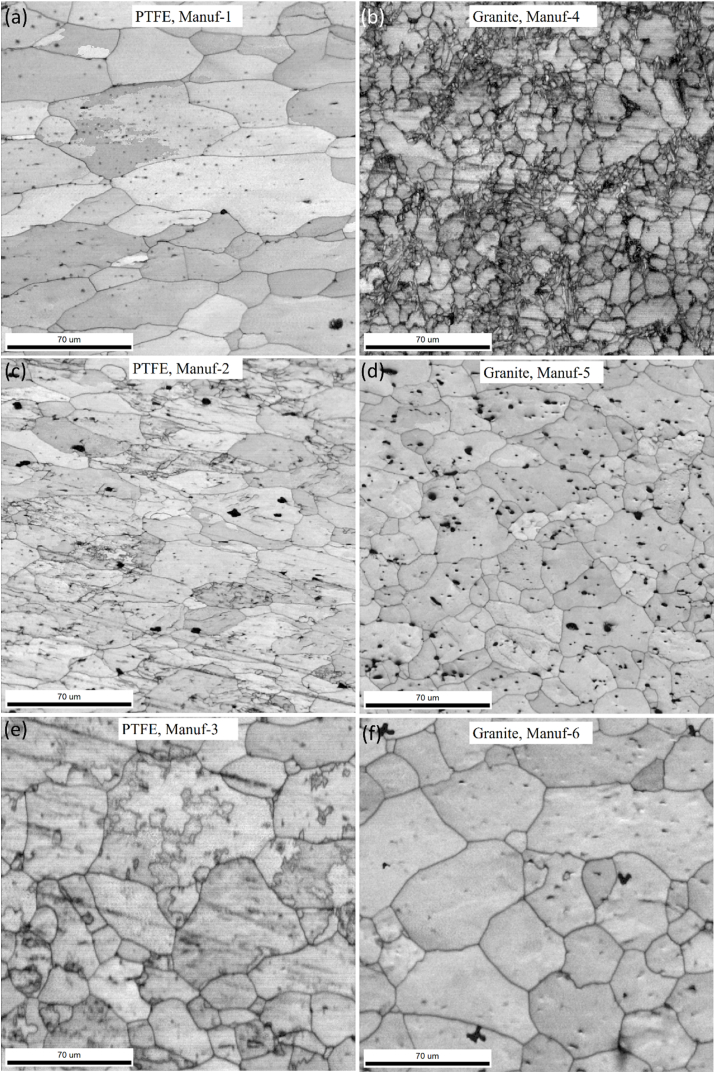
SEM-EBSD IQ (image quality) map of cookware heated at 250 °C for 2 h produced by different suppliers(a) PTFE Manuf-1; (b) Granite Manuf-4; (c)PTFE Manuf-2; (d) Granite Manuf-5; (e) PTFE Manuf-3; (f) Granite Manuf-6.

As per prior studies [37,38], heating Al alloys induces grain growth, grain softening, and precipitate formation. Dynamic softening involves dynamic recovery (DRV) and dynamic recrystallization (DRX), where DRV alters dislocations, softening materials [39]. Concurrently, DRX forms through continuous dynamic recrystallization (CDRX) and discontinuous dynamic recrystallization (DDRX) [40]. These processes impact low-angle grain boundaries (LAGBs) and high-angle grain boundaries (HAGBs) (Fig. 13). The misorientation angle distribution in every pan indicates structural changes during heating. The formation of FeAl_3_ precipitates within or along grain boundaries is evident in the SEM-EBSD IQ map (Fig. 14). Fine grains with more FeAl_3_ precipitates are observed in Granite Manuf-4 cookware (Fig. 14b). Each cookware exhibits distinct grain morphology and FeAl_3_ precipitate patterns. PTFE Manuf-2 cookware shows elongated grains with few FeAl_3_ precipitates (Fig. 14c), while Granite Manuf-5 displays equiaxed grains with FeAl_3_ precipitates (Fig. 14d). PTFE Manuf-1 (Fig. 14a) and Granite Manuf-6 (Fig. 14f) exhibit very large grains with limited elongation and FeAl_3_ precipitates. In summary, temperatures exceeding 175 °C for 2 h significantly impact the internal structures of all cookware, aligning with previous findings [12].

### 3.4 Examination of wear performance using Taber abrasion test and surface roughness of cookware

**Fig. 15:**
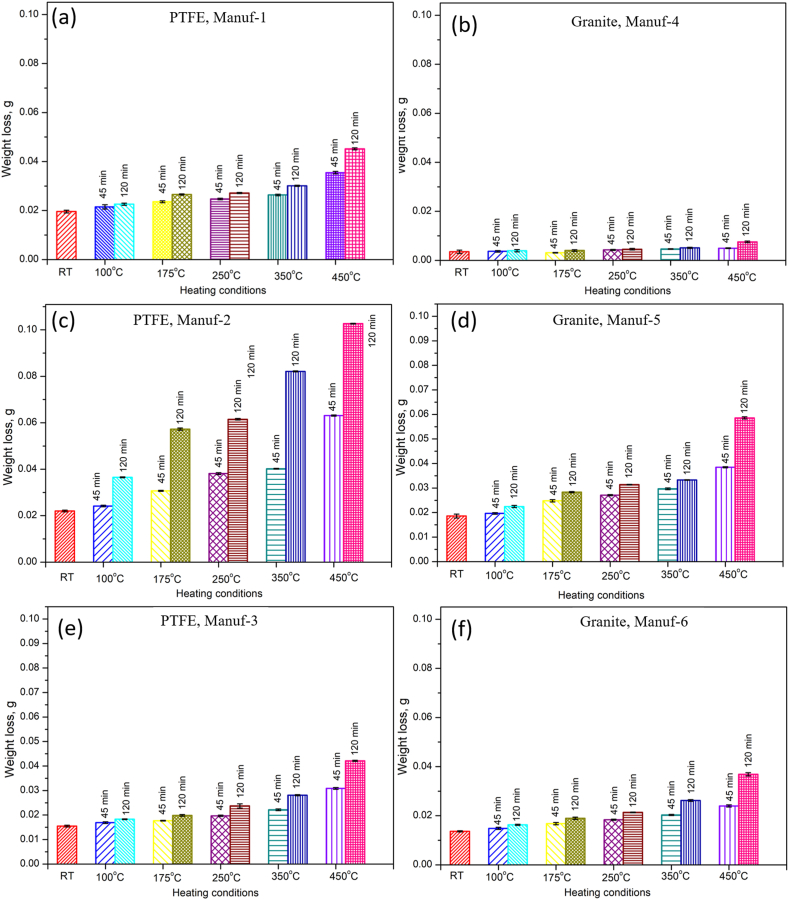
Variation of weight loss after Taber rotary abrasion wear test for all cookware heated with different conditions (RT, 100, 175, 250, 350, & 450 °C for 45 & 120 min): (a) PTFE Manuf-1; (b) Granite Manuf-4; (c) PTFE Manuf-2; (d) Granite Manuf-5; (e) PTFE Manuf-3; and (f) Granite Manuf-6.

Kitchen tools, including brushes and sponges, often cause mechanical damage to non-stick surfaces during use. Furthermore, contact frying with meat is usually carried out at temperatures higher than 200 °C, which causes wear [5]. The Taber abrasion test, a suitable method for assessing abrasion damage [41,42], was conducted. The results, detailed in the Appendices, reveal the weight loss variation after the Taber abrasion rotary test (see Fig. 15). Generally, wear increases significantly with rising temperature and time for all cookware, irrespective of manufacturers, which is expected to form a fouling layer while cooking (due to burnt deposits). For PTFE pans from Manuf-1, weight loss percentages compared to RT after 45 min heating were 9.7 %, 20.48 %, 26.02 %, 34.7 %, and 81.12 % for temperatures 100, 175, 250, 350, and 450 °C, respectively. At 120 min heating, the weight loss percentages compared to RT were 15.3 %, 35.71 %, 38.7 %, 58.16 %, and 130.6 % for 100, 175, 250, 350, and 450 °C, respectively. Similar trends were observed in other PTFE pans. For ceramic-coated cookware, at 45 min heating, Granite pan of Manuf-4 exhibited weight loss percentages compared to RT of 5.4 %, 11.31 %, 22 %, 32.31 %, and 41.43 % for temperatures 100, 175, 250, 350, and 450 °C, respectively. The weight loss increased at 120 min heating to 12.68 %, 15.41 %, 29.58 %, 46.85 %, and 115.21 % for 100, 175, 250, 350, and 450 °C, respectively, demonstrating the loss of mechanical strength with increasing temperature and time in which higher temperature and long heating time influenced much. The wear behavior, detailed in Appendices, indicates that severe wear occurred in certain PTFE and Granite pans, attributed to the low tensile strength (Table 6), and poor bonding of coated materials which is expected to enhance the inert surface chemistry of cookware [43]. Overall, non-stick cookware exhibited drastic wear after heating beyond 350 °C, emphasizing the importance of using cookware below 250 °C to minimize wear [12], which may lead to a decrease in the fouling layer that usually occurs in the cookware. In other words, more wear occurs beyond 250 °C, which was attributed to poor cohesive and adhesive forces between C-F/silane molecules and the substrate [44]. Additionally, ceramic-coated cookware showed less wear, indicating higher wear and thermal resistances compared to PTFE-coated non-stick cookware. TWI results, discussed in Appendices, align with the observed wear loss phenomena.

**Fig. 16:**
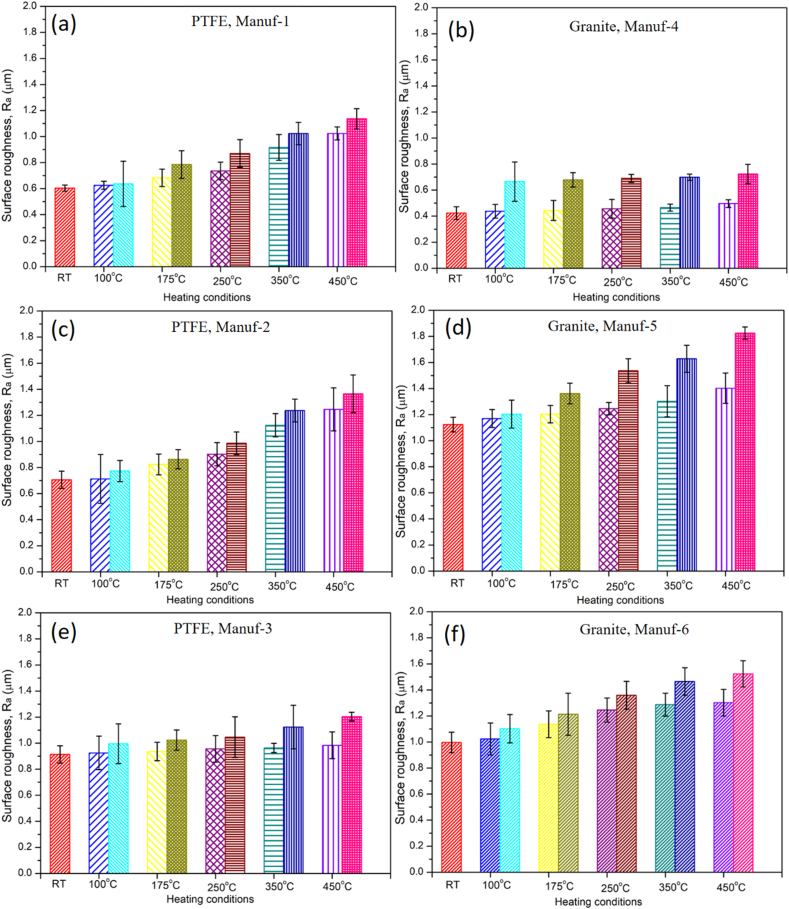
Variation of surface roughness value on cookware after Taber abrasion test heated with different conditions (RT, 100, 175, 250, 350, & 450 °C for 45 & 120 min): (a) PTFE Manuf-1; (b) Granite Manuf-4; (c) PTFE Manuf-2; (d) Granite Manuf-5; (e) PTFE Manuf-3; and (f) Granite Manuf-6.

The study involved measuring surface roughness of clean and damaged surfaces resulting from the Taber abrasion test after heating at different temperatures and times. The appended results detail the surface roughness (Ra) of all cookware, presented in Fig. 16. Fig. 16 demonstrates a clear increase in surface roughness values with rising temperatures and time. This phenomenon is attributed to weakened bonding of C-F atoms in PTFE-coated non-stick cookware and the weakened bonding of silane in ceramic-coated counterparts. Among the cookware investigated, Granite Manuf-4 exhibited lower surface roughness values, indicating strong bonding and a higher concentration of silane materials over the substrate. Conversely, Granite Manuf-5, Granite Manuf-6, and PTFE Manuf-2 non-stick cookware showed the highest surface roughness values, suggesting weaker bonding and lower coating material concentration over the substrates. Up to 250 °C, the observed surface roughness values remained within nominal and acceptable levels, indicating that Al-based non-stick cookware can be heated to 250 °C. Beyond this temperature, other suppliers (PTFE Manuf-2, Granite Manuf-5, PTFE Manuf-3, and Granite Manuf-6) exhibited significantly weakened bonding of C-F atoms and silane compounds over the substrate [12]. Statistically, for instance, at 45 min heating, the variation of percentage of surface roughness value of PTFE Manuf-1 non-stick cookware's compared to RT was 3.63 %, 13.26 %, 22.09 %, 51.89 %, and 69.68 % for 100, 175, 250, 350, and 450 °C, respectively. Whereas, at 120 min heating, the variation of percentage of surface roughness value as function of temperature compared to RT sample was 5.51 %, 30.18 %, 44.19 %, 69.64 %, and 88.60 % for 100, 175, 250, 350, and 450 °C, respectively. These results demonstrate clearly the influences of higher temperature and long heating time affecting the performances. Further statistical details are available in the Appendices.

### 3.5 Examination of pull-off adhesive strength of cookware supplied by different manufacturers

**Fig. 17:**
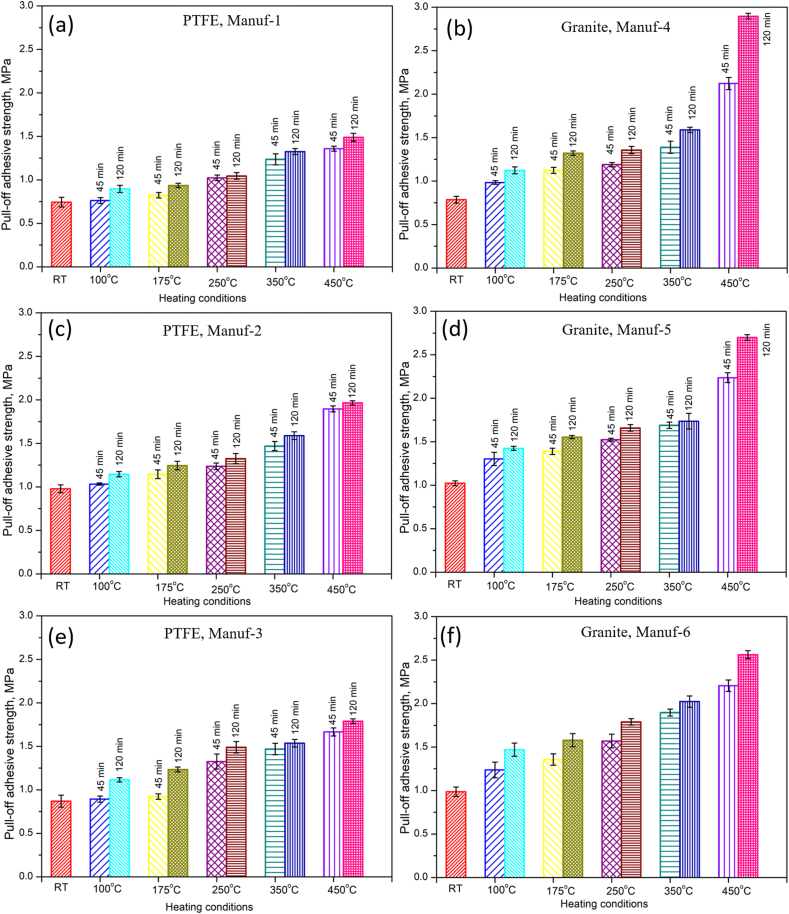
Variation of Pull-off adhesive strength after uniaxial tensile pull-off test for all cookware's heated with different conditions (RT, 100, 175, 250, 350, & 450 °C for 45 & 120 min): (a) PTFE Manuf-1; (b) Granite Manuf-4; (c) PTFE Manuf-2; (d) Granite Manuf-5; (e) PTFE Manuf-3; and (f) Granite Manuf-6.

In the conducted study, an adhesive pull-off test [[45], [46], [47]] was performed to assess stress detachment values after heating pans to varying temperatures and durations. The measured pull-off adhesive strength values are detailed in the Appendices, and Fig. 17 illustrates the variation of pull-off adhesive strength for all cookware. Results indicate a consistent increase in pull-off adhesive strength with rising temperature and time across all cookware, irrespective of manufacturers. The magnitude of this increase varies among manufacturers, attributed to differences in chemical composition (Table 5) and mechanical properties (Table 6) of the as-received pans. For PTFE Manuf-1 non-stick pans after 45 min heating, pull-off strength percentages increased by 2.63 %, 10.75 %, 37.64 %, 66.27 %, and 82.7 % for temperatures 100, 175, 250, 350, and 450 °C, respectively. At 120 min heating, the variation of percentage of adhesive pull-off strength as function of temperature compared to RT pans was 20.54 %, 25.938 %, 40.60 %, 78.26 %, and 100.3 % for 100, 175, 250, 350, and 450 °C, respectively. The increasing of adhesive pull-off strength after 120 min heating indicates the loss of more C-F atoms in PTFE coating material. PTFE Manuf-2 and PTFE Manuf-3 showed varying adhesive pull-off strengths. For Granite coated cookware, Manuf-4 pan exhibited increasing adhesive pull-off strength percentages compared to RT of 25.09 %, 42.89 %, 51.3 %, 76.71 %, and 169.98 % for temperatures 100, 175, 250, 350, and 450 °C, respectively. More adhesive pull-off strength in Granite Manuf-4 pans compared to PTFE Manuf-1 suggested weaker silane compounds over the ceramic cookware. Overall, results indicate a drastic decrease in bonding strength of C-F atoms in PTFE non-stick cookware and silane bonding materials in Ceramic non-stick cookware with increasing temperature and time. PTFE Manuf-1, PTFE Manuf-3, and Granite Manuf-4 non-stick cookware maintained coating materials up to 250 °C, while others exhibited weak bonding beyond 250 °C [12]. Further details are provided in the Appendices.

### 3.6 Examination of room temperature corrosion behavior of as-received cookware

**Fig. 18:**
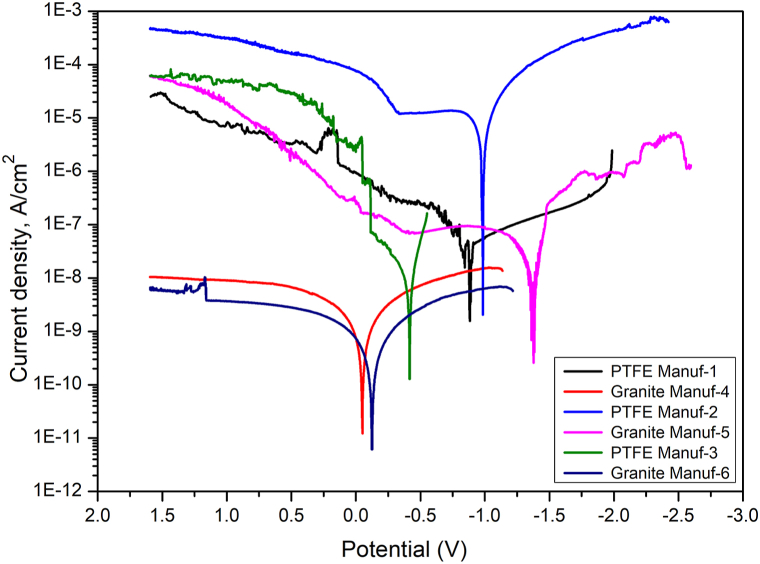
Potentiodynamic polarization curves during room temperature corrosion test of as-received cookware produced by different suppliers.

Fig. 18 displays potentiodynamic polarization (PDP) curves for six as-received cookware samples tested at room temperature, immersed in 3.5 % NaCl solution for 120 min. Table 7 presents corrosion potential (E_corr_) and corrosion current density (I_corr_) parameters. According to electrochemical theories, lower corrosion current density and higher corrosion potential signify enhanced corrosion resistance [48,49]. Results in Table 7 reveal the following order for corrosion current densities: Granite Manuf-4 > Granite Manuf-6 > PTFE Manuf-3 > PTFE Manuf-1 > Granite Manuf-5 > PTFE Manuf-2. This pattern aligns with the corrosion potential. Granite Manuf-4 cookware exhibits the lowest current density (6.82*10^−8^ A/cm^2^), indicating superior corrosion resistance, while PTFE Manuf-2 cookware has the highest current density, signaling poor corrosion resistance.

**Table 7:** Potentiodynamic polarization parameters during room temperature corrosion test of as-received cookware produced by different suppliers

Name of sample	E_Corr_ (V)	I_Corr_ (A/cm^2^)
PTFE Manuf-1	−0.89	6.82*10^−8^
Granite Manuf-4	−0.03	4.11*10^−10^
PTFE Manuf-2	−1.01	5.28*10^−6^
Granite Manuf-5	−1.36	2.64*10^−8^
PTFE Manuf-3	−0.387	7.55*10^−9^
Granite Manuf-6	−0.09	1.84*10^−10^

**Fig. 19:**
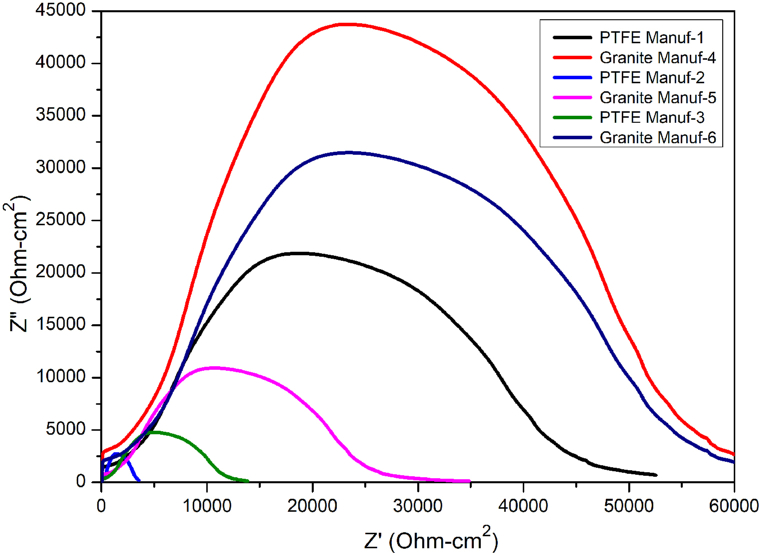
Nyquist plots during room temperature corrosion test of as-received cookware's produced by different suppliers.

Nyquist plots provide valuable insights into corrosion potential during electrochemical testing. Typically, Nyquist plots manifest as semicircle arcs, as the circuit curve of a corroded sample can be represented by trigonometric functions [48]. A larger area of Nyquist plots indicates higher corrosion resistance. Fig. 19 displays Nyquist plots for the as-received cookware, and the results align with those from PDP curves. Therefore, it can be concluded that Granite Manuf-4 ceramic cookware demonstrates superior corrosion resistance compared to other samples.

**Fig. 20:**
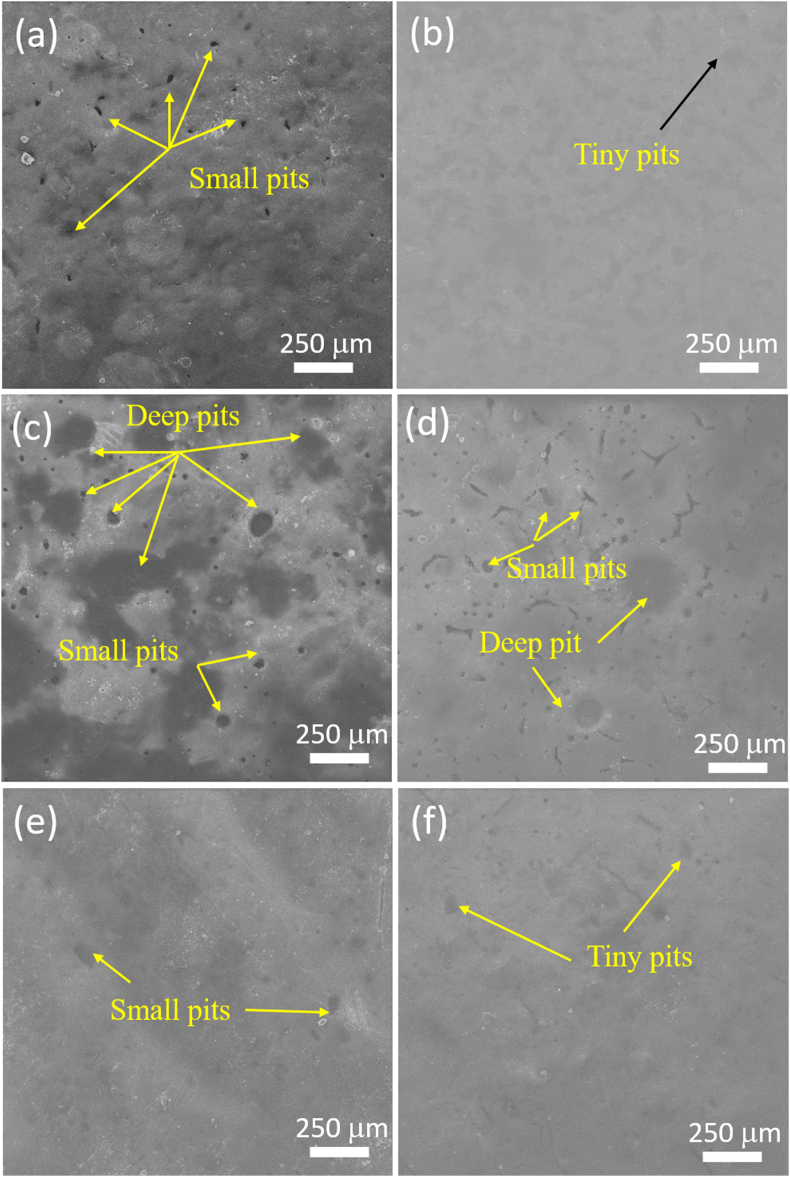
SEM surface morphology of corroded area after room temperature corrosion test for the as-received cookware's: (a) PTFE Manuf-1; (b) Granite Manuf-4; (c)PTFE Manuf-2; (d) Granite Manuf-5; (e) PTFE Manuf-3; (f) Granite Manuf-6.

Fig. 20a–f presents SEM morphologies of corroded cookware samples tested at room temperature. Granite Manuf-4 and Granite Manuf-6 exhibited minimal pits (Fig. 20b and f), indicating enhanced corrosion resistance with oxide layer formation. PTFE Manuf-2 surfaces displayed severe damage, featuring deep 200 μm pits due to intense chloride ion interaction (Fig. 20c). Pits initiated localized pitting corrosion, leading to crystallographic pit formation. Granite Manuf-5 exhibited smaller pits (Fig. 20d), while PTFE Manuf-3 showed non-uniformly distributed pits of around 35 μm (Fig. 20e). Granite Manuf-6 demonstrated few tiny pits, highlighting better corrosion resistance. SEM morphologies align with polarization curves and Nyquist plots, confirming corrosion behavior.

### 3.7 Examination of hot oxidation and corrosion performance of cookware's

**Fig. 21:**
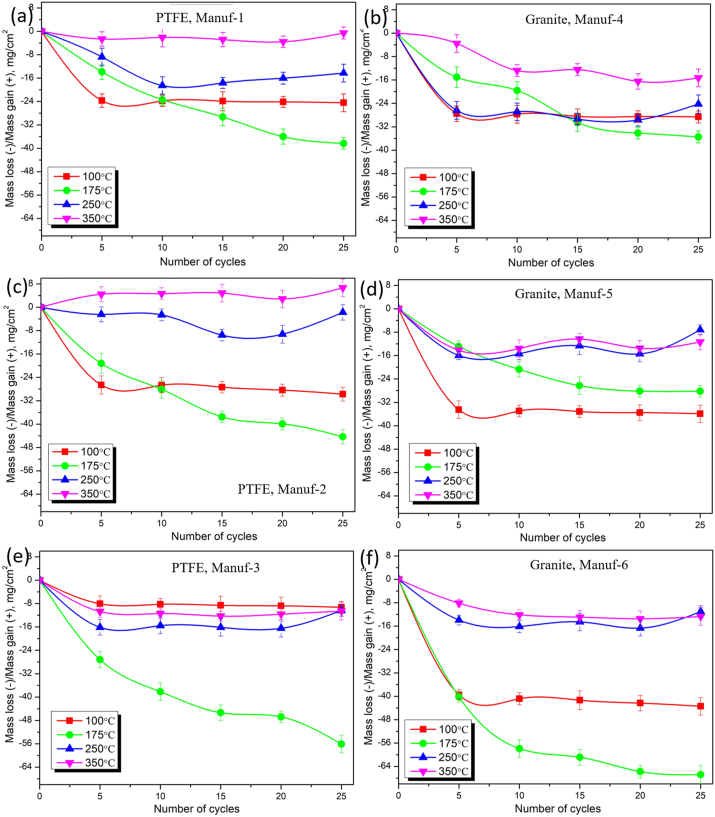
Hot corrosion performance of mass loss (-ve)/mass gain (+ve) with the function of several cycles (hrs) and temperatures (100, 175, 250, and 350 °C): (a) PTFE Manuf-1; (b) Granite Manuf-4; (c) PTFE Manuf-2; (d) Granite Manuf-5; (e) PTFE Manuf-3; and (f) Granite Manuf-6.

**Fig. 22:**
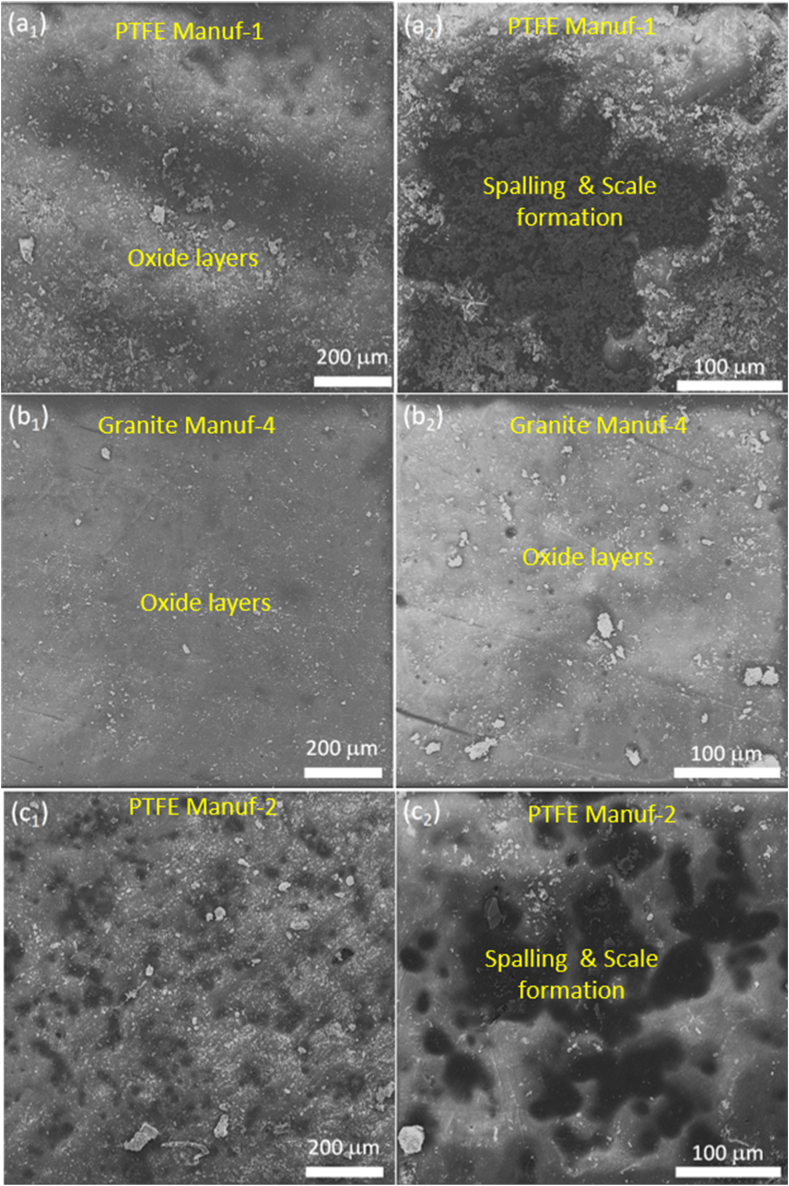

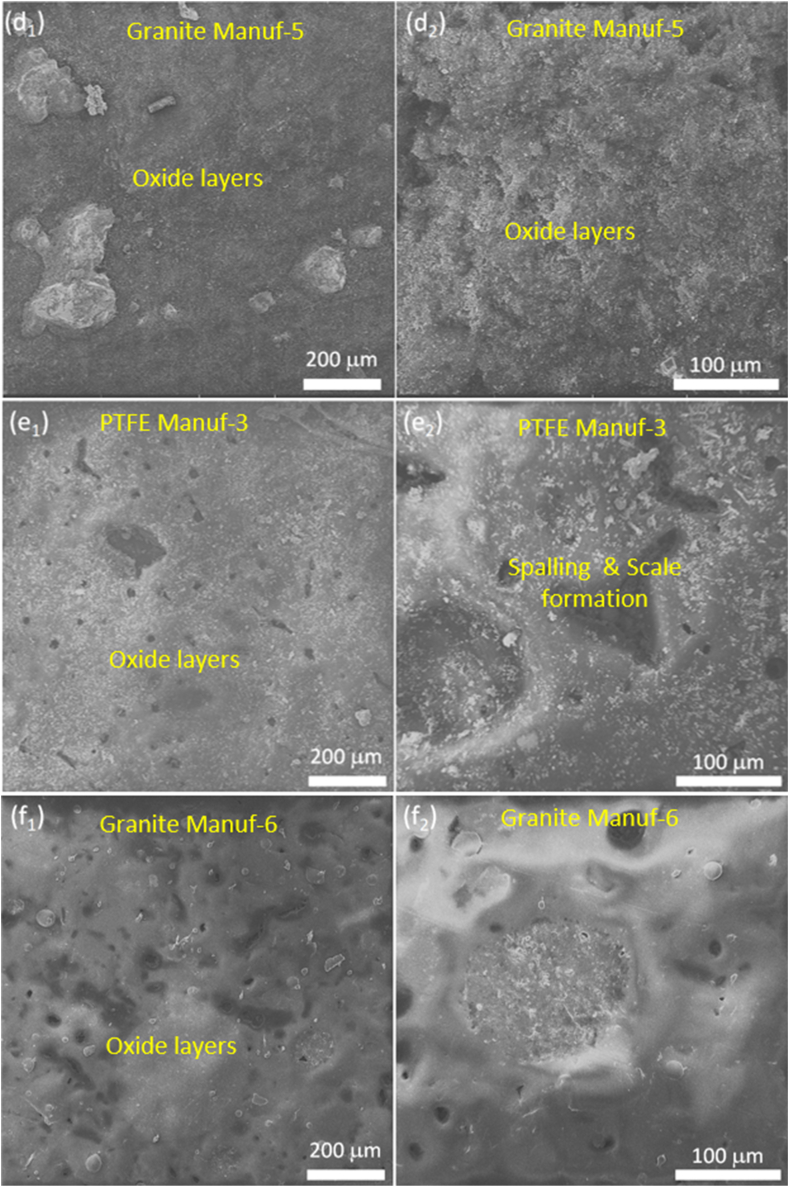
SEM surface morphology of non-stick cookware after hot oxidation and hot corrosion test at 100 °C after 25 number of cycles: (a1) & (a2) PTFE Manuf-1; (b1) & (b2) Granite Manuf-4; (c1) & (c2) PTFE Manuf-2; (d1) & (d2) Granite Manuf-5; (e1) & (e2) PTFE Manuf-3; (f1) & (f2) Granite Manuf-6.

The study investigated the hot oxidation and corrosion performance of as-received cookware, considering cycles (0, 5, 10, 15, 20, & 25 h) and temperatures (100, 175, 250, & 350 °C). Fig. 21 illustrates cumulative weight loss (-ve) and gain (+ve) per unit area with cycles and temperatures, detailed in Appendices. Results indicate temperature and time significantly impact cookware performance, with varied weight loss and gain among suppliers. Scale formation during tests contributes to weight gain, while salt evaporation, spalling, pit formation, and dissolution lead to weight loss [32,50]. Fig. 21 shows increased weight loss at 175 °C due to oxide layer formation and spalling. At 100 °C, weight loss occurs early, decreasing slightly from 5 to 25 h. At 175 °C, spalling dominates, decreasing weight loss. However, at 250 °C and 350 °C, weight loss decreases, and gain increases due to protective oxide layer breakage and oxide particle penetration. PTFE Manuf-2 cookware exhibits severe scale formation, followed by PTFE Manuf-1. Fig. 22 (SEM images) depicts oxide layer formation and spalling, with more observed in PTFE-coated cookware (Fig. 22a_1_ & a_2_; c_1_ & c_2_; e_1_ & e_2_) and less in ceramic-coated counterparts (Fig. 22b_1_ & b_2_; d_1_ & d_2_; f_1_ & f_2_).

## 4 Conclusion and recommendations

The study compared PTFE-based and ceramic-based non-stick cookware made of forged aluminum alloy through tests involving Taber abrasion, adhesive pull-off, and surface roughness. Key findings include.

❖ Cookware composition varied, with PTFE Manuf-1, PTFE Manuf-2, PTFE Manuf-3, Granite Manuf-4, Granite Manuf-5, and Granite Manuf-6 made from AA 1050, AA 1100, AA 1230, AA 4145, AA 1100, and AA 1145 series, respectively.
❖ Optical microstructural analysis revealed α-Al and FeAl_3_ precipitates, with Granite Manuf-4 exhibiting Al_12_SiFe_3_ intermetallic phase and Mg_2_Si residues.
❖ Taber wear test indicated substantial wear in PTFE Manuf-2 (0.0242g after 45 min heating at 100 °C, 0.0631g after 45 min heating at 450 °C) and Granite Manuf-5 (0.0197g after 45 min heating at 100 °C, 0.0385g after 45 min heating at 450 °C) due to low mechanical properties. Granite Manuf-4 (0.0037g after 45 min heating at 100 °C, 0.0046g after 45 min heating at 450 °C) showed minimal wear due to effective silane coating and refined microstructures, indicating superior wear resistance compared to other combinations.
❖ Severe wear occurred in all cookware beyond 250 °C, suggesting usage below this temperature. Ceramic-coated cookware exhibited less wear, indicating higher wear resistance than PTFE-coated counterparts.
❖ Adhesive pull-off tests demonstrated increasing strength with temperature and time, attributed to loss of C-F atoms in PTFE and silane in ceramic coatings. Temperature had a more pronounced effect than heating time. After 120 min heating, PTFE Manuf-1 (0.90 MPa at 100 °C, 1.49 MPa at 450 °C) and Granite Manuf-4 (1.12 MPa at 100 °C, 1.5893 MPa at 450 °C) exhibited lowest adhesive pull-off strength compared to PTFE Manuf-2 (1.14 MPa at 100 °C, 1.96 MPa at 450 °C) and Granite Manuf-5 (1.42 MPa at 100 °C, 2.70 MPa at 450 °C) samples representing effective bonding of C-F atoms and silane, respectively.
❖ Surface roughness increased with temperature and time due to weak bonding. Granite Manuf-4 exhibited lower roughness (0.74 μm at 450 °C after 120 min heating), indicating stronger bonding.
❖ Hot corrosion tests at 175 °C showed increased weight loss due to oxide layers and spalling. At 250 °C and 350 °C, weight loss decreased, and weight gain increased due to protective oxide layer breakdown.
❖ PTFE Manuf-2 exhibited the highest weight gain (6.67 mg/cm^2^ after 25 no of cycles operated at 350 °C), indicating severe scale formation. Ceramic-coated cookware showed less spalling and oxide layers.
❖ Room temperature corrosion tests ranked cookware resistance as Granite Manuf-4 > Granite Manuf-6 > PTFE Manuf-3 > PTFE Manuf-1 > Granite Manuf-5 > PTFE Manuf-2. Lowest current density was obtained from Granite Manuf-4 (4.11*10^−10^, A/cm^2^) indicate better corrosion resistance. Highest current density of 5.28 × 10^−6^ A/cm^2^ was obtained in PTFE Manuf-2 cookware indicating very poor in corrosion resistance
❖ Microstructure and XRD analysis revealed fine grains and high dislocation density in Granite Manuf-4, indicating superior performance compared to other pans.

## Funding

This research was funded by the Saudi Standards, Metrology, and Quality Organization (SASO), General Department of Research and Studies, Research and Studies Center, grant number (7-12).

## Data availability statement

The experimental datasets obtained from this research work and then the analyzed results during the current study are available from the corresponding author on reasonable request.

## CRediT authorship contribution statement

**Abdulaziz S. Alaboodi:** Writing – review & editing, Resources, Project administration, Conceptualization. **S. Sivasankaran:** Writing – original draft, Validation, Methodology, Investigation, Formal analysis, Conceptualization. **Hany R. Ammar:** Investigation, Formal analysis, Data curation.

## Declaration of competing interest

The authors declare that they have no known competing financial interests or personal relationships that could have appeared to influence the work reported in this paper.
