# Supplementary material for: Influence of heating temperature and time on mechanical-degradation, microstructures and corrosion performances of Teflon/granite coated aluminum alloys used for non-stick cookware
Source: Heliyon. 2024 Jul 20;10(14):e34676. doi: 10.1016/j.heliyon.2024.e34676 (PMC11324993; doi:10.1016/j.heliyon.2024.e34676)
Supplement: Multimedia component 1 [file mmc1.pdf]

Supplementary Materials

These non-stick agents coated over the pans (Aluminum, stainless steel, cast iron, or any ceramic materials) should usually be non-toxic and never harm the human organs. If all the non-stick cookware is appropriately used, it will never create any issues. However, if it is not used in proper conditions (temperature, heating methods, washing procedure, etc.), it will generate some problems that will affect human life.

Fig S1 shows the PTFE coated (Teflon) sauce and fry pans and Fig S2 and Fig S3 show the ceramic coated (silica and granite) cookware's.

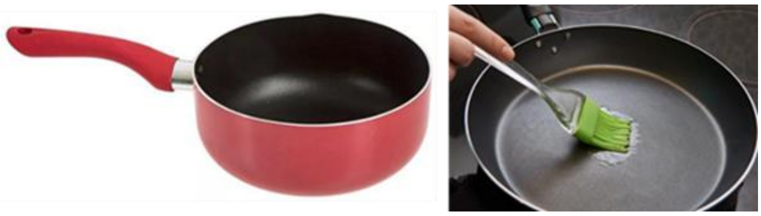

Fig. S1. Teflon (PTFE) coated sauce and fry pans.

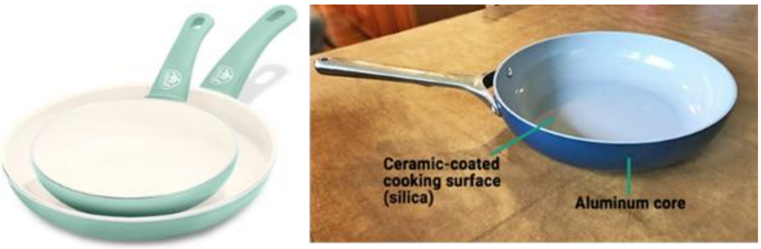

Fig. S2. Ceramic (silica-SiO_2) coated cookware/pan.

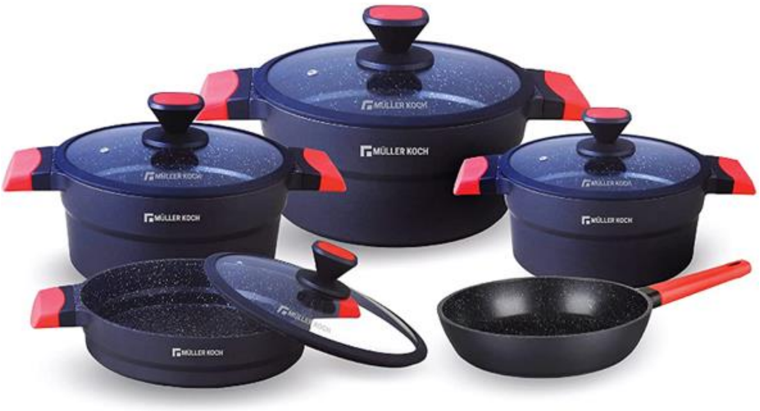

Fig. S3. Granite coated Aluminum cookware.

Fig. S3 shows the photograph of heating of as-purchased pans supplied by various manufactures which are all heated at different temperatures and time using infrared electric oven (100, 175, and 250°C) and electric induction furnace (350, and 450°C).

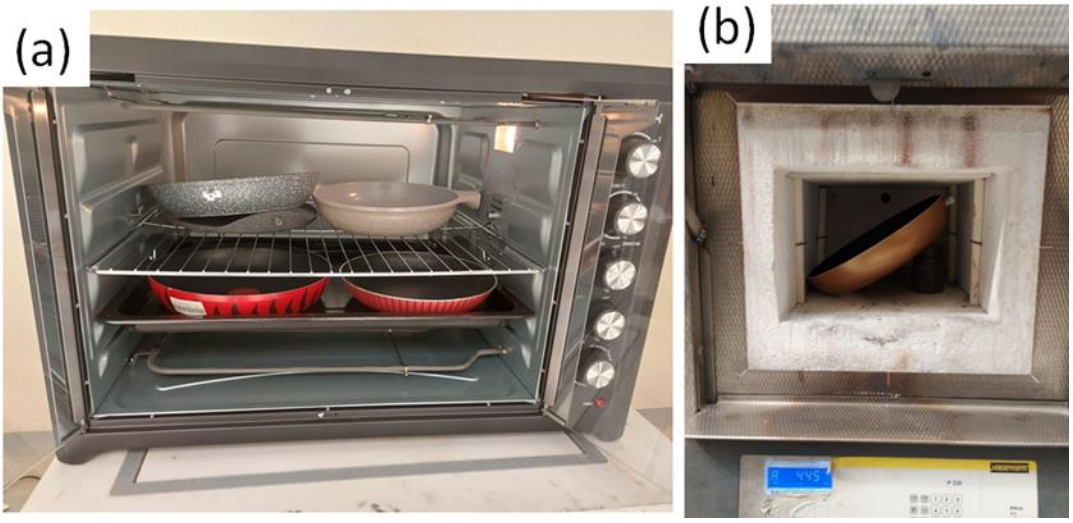

Figure S4 Photograph showing the heating source of as-purchased pans made from different manufactures using temperature controlled equipment: (a) electric infrared stove/oven; (b) electric induction furnace

When the two abraded wheels rotate, the turn-able sample against sliding rotation of two abraded wheels introduces rub-wear action. Here, the two abraded wheels (one is left, and the other is right) rotate opposite each other on a horizontal axis by the sample. One abraded wheel rubs the sample inward towards the center, whereas the other rubs outward towards the periphery during the test. A vacuum system will be connected to extract the debris produced during the test.

Further, two abraded arms can be lowered or raised for examining the sample, which are independently operating ones. Each arm is a precisely balanced one. The test is usually carried out by fixing the number of cycles like 500, 1000, and 1500 at 60 or 72 rpm speed, which a speed regulator can vary. The evaluation criteria for the cookware is a loss in coating materials over the substrate. Hence, the Taber abrasion test is selected in this research work. After heating the pans, a circular disc was cut with a diameter of 105 mm using a laser cutting machine. F shows the photograph of circular-shaped cut samples prepared for the Taber abrasion test from cookware supplied by different manufacturers at different heating temperatures and times.

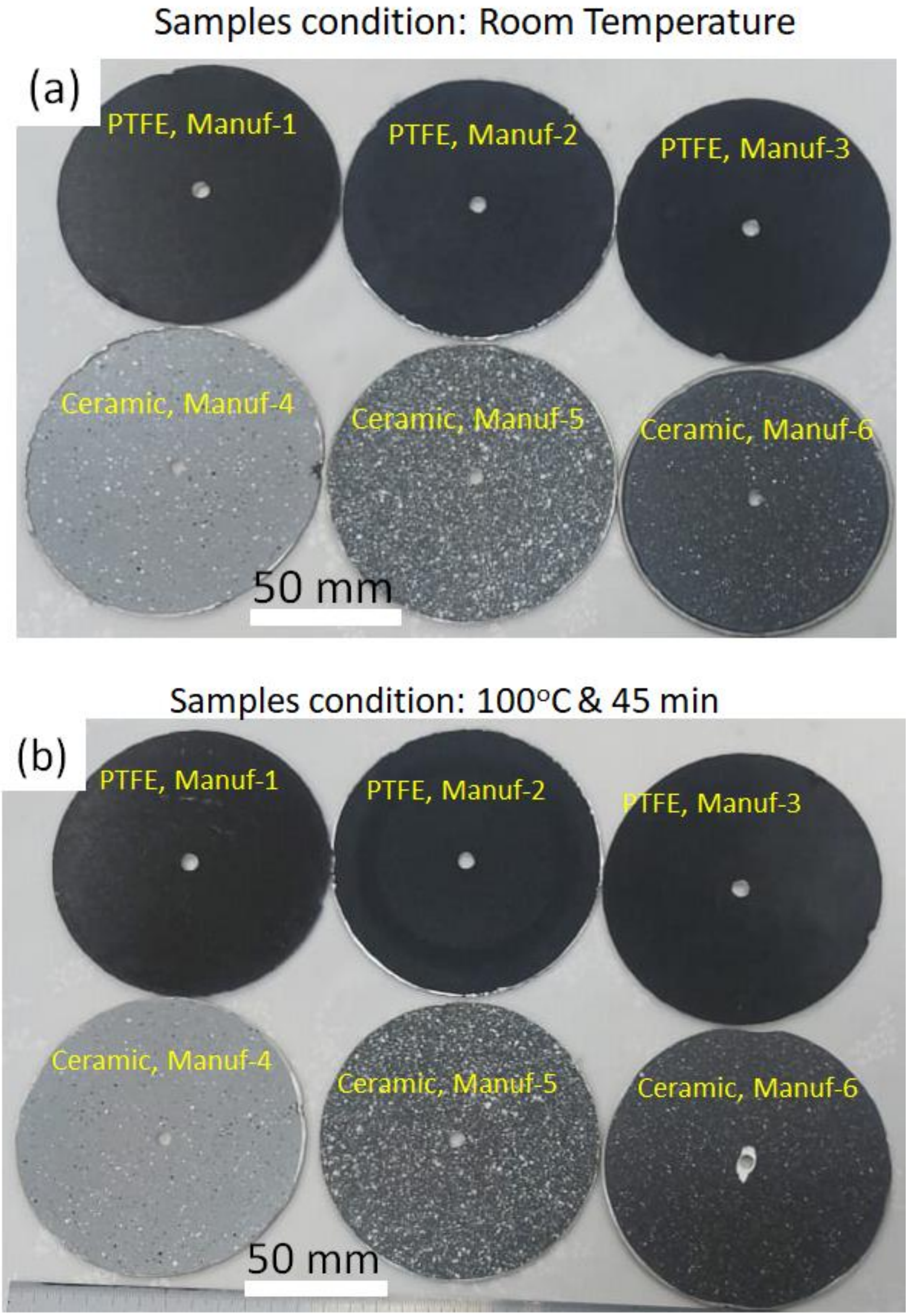
Samples condition: Room Temperature
(a)
PTFE, Manuf-1
PTFE, Manuf-2
PTFE, Manuf-3
Ceramic, Manuf-4
Ceramic, Manuf-5
Ceramic, Manuf-6
50 mm
Samples condition: 100°C & 45 min
(b)
PTFE, Manuf-1
PTFE, Manuf-2
PTFE, Manuf-3
Ceramic, Manuf-4
Ceramic, Manuf-5
Ceramic, Manuf-6
50 mm

Samples condition: 100°C & 120 min

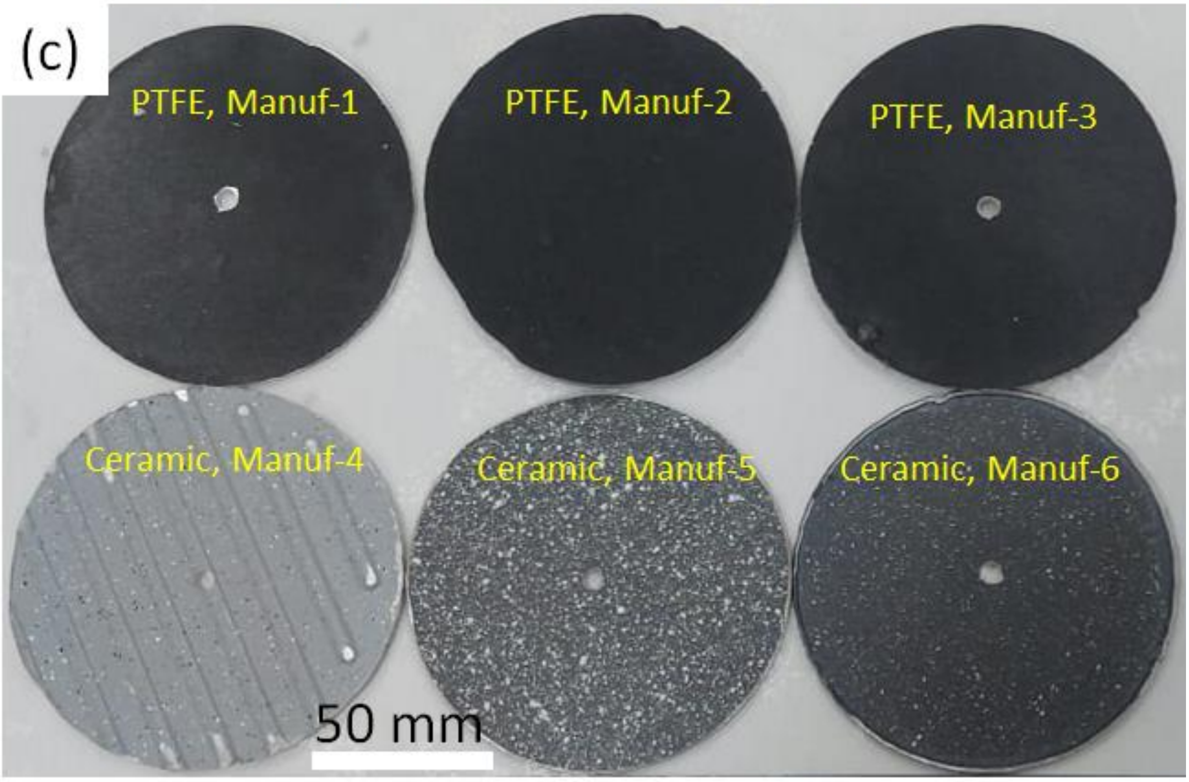

Samples condition: 175°C & 45 min

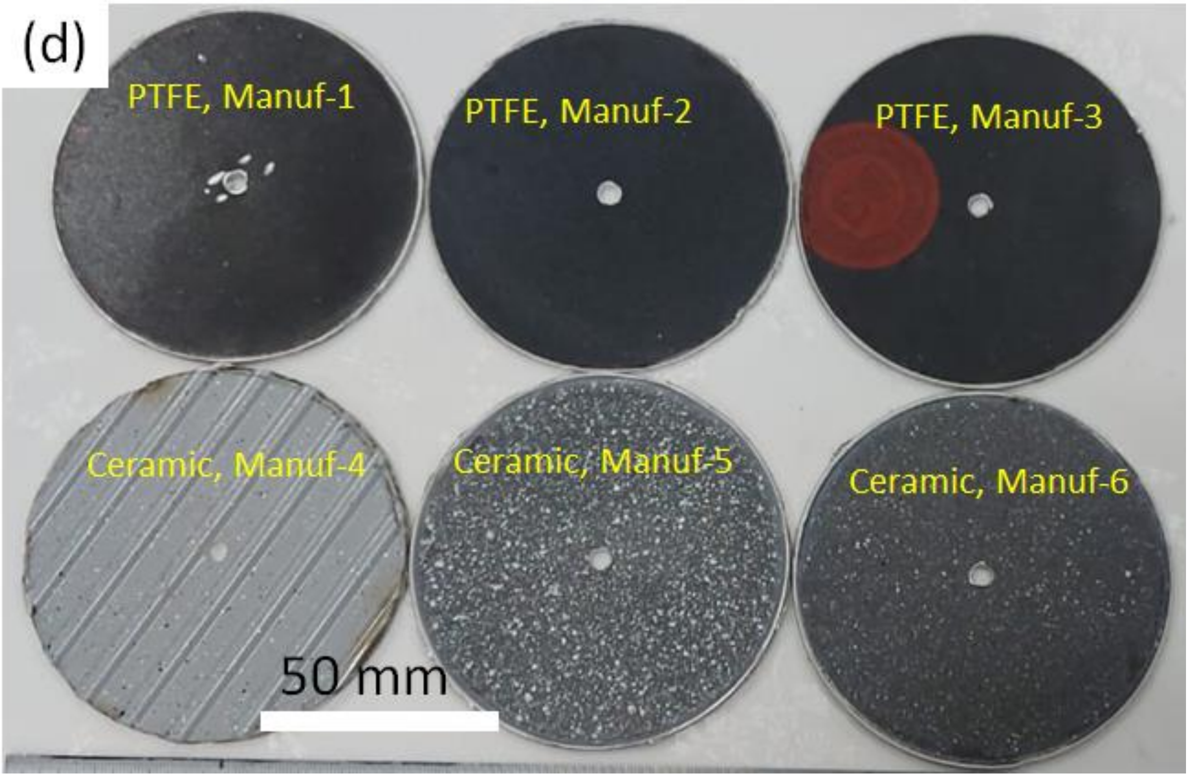

Samples condition: 175°C & 120 min

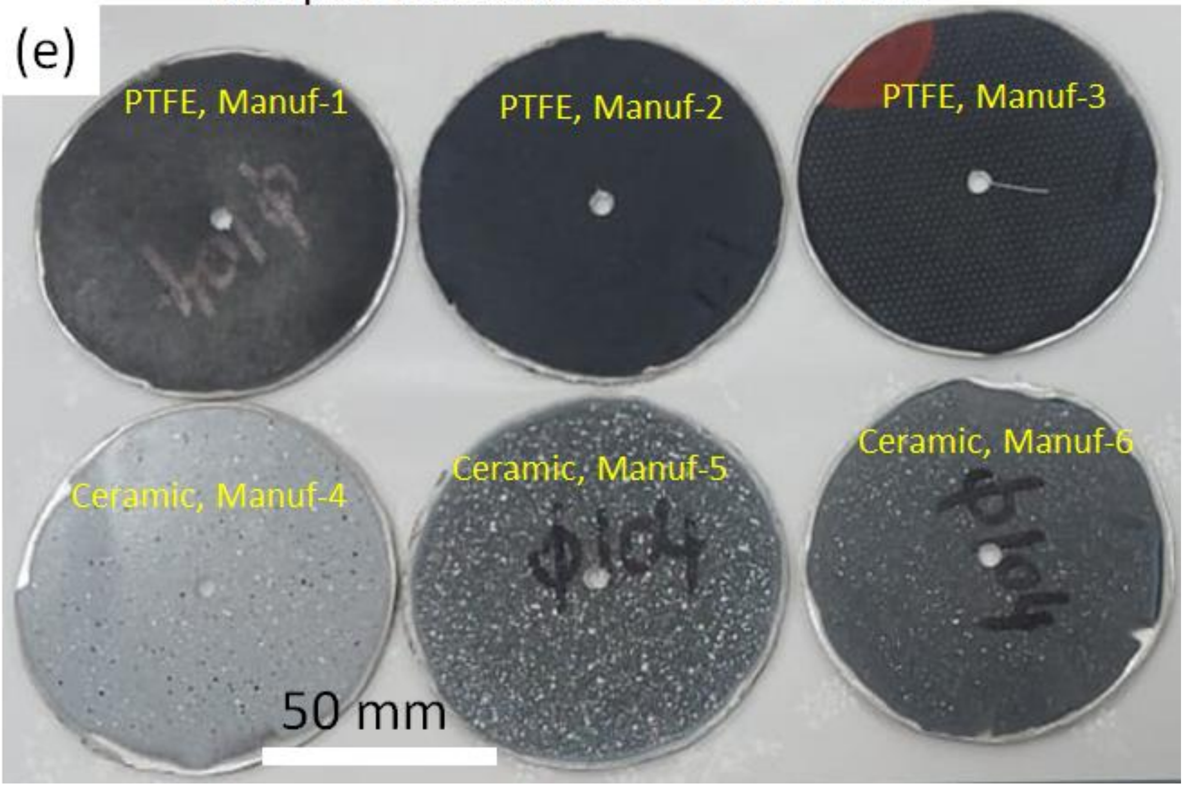

Samples condition: 250°C & 45 min

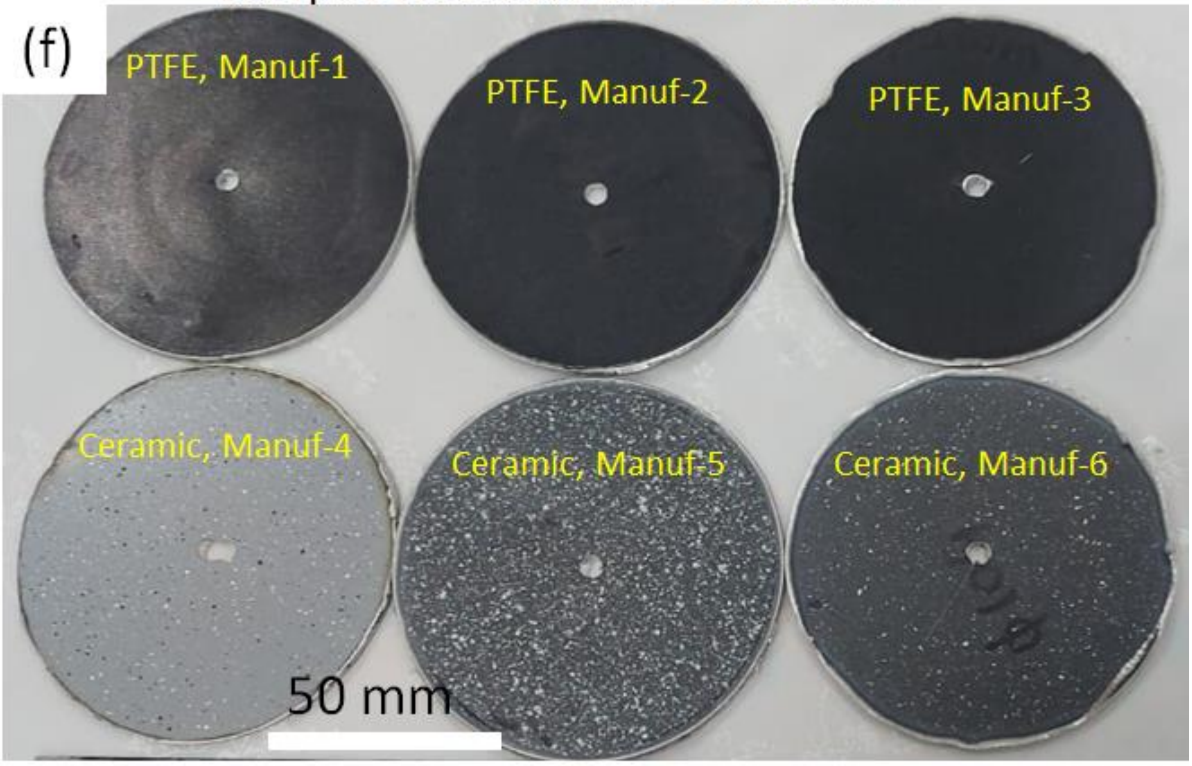

Samples condition: 250°C & 120 min

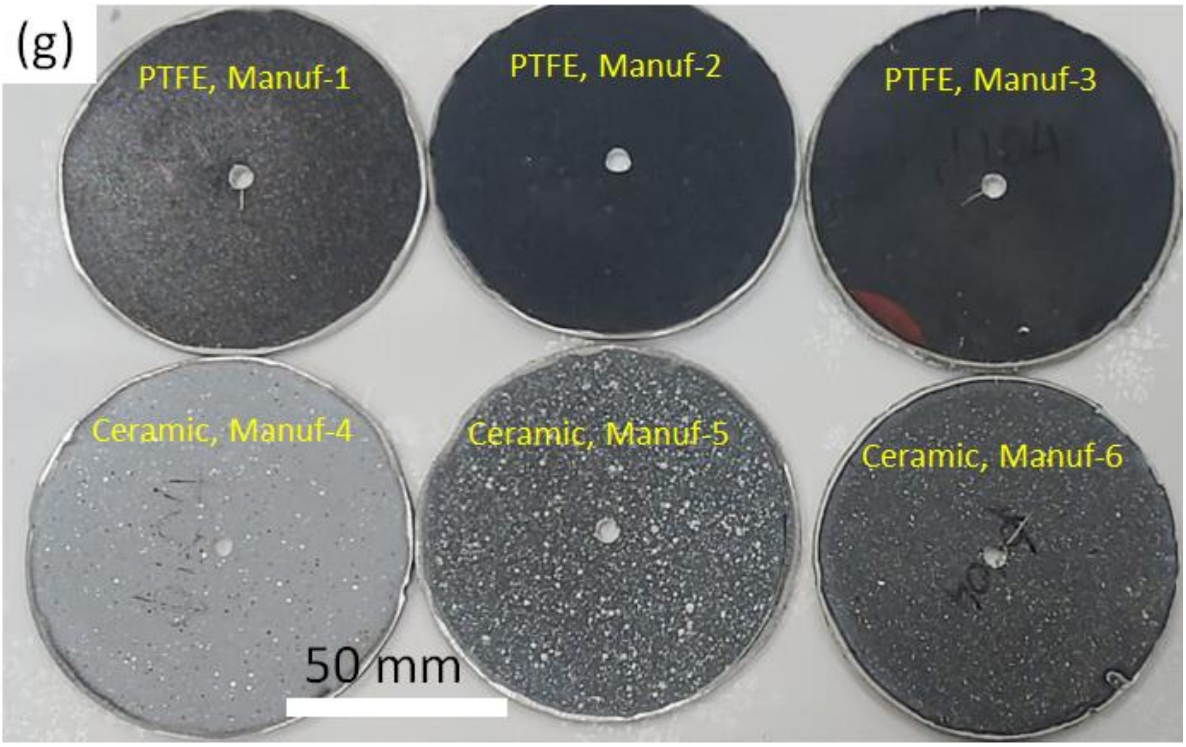

Samples condition: 350°C & 45 min

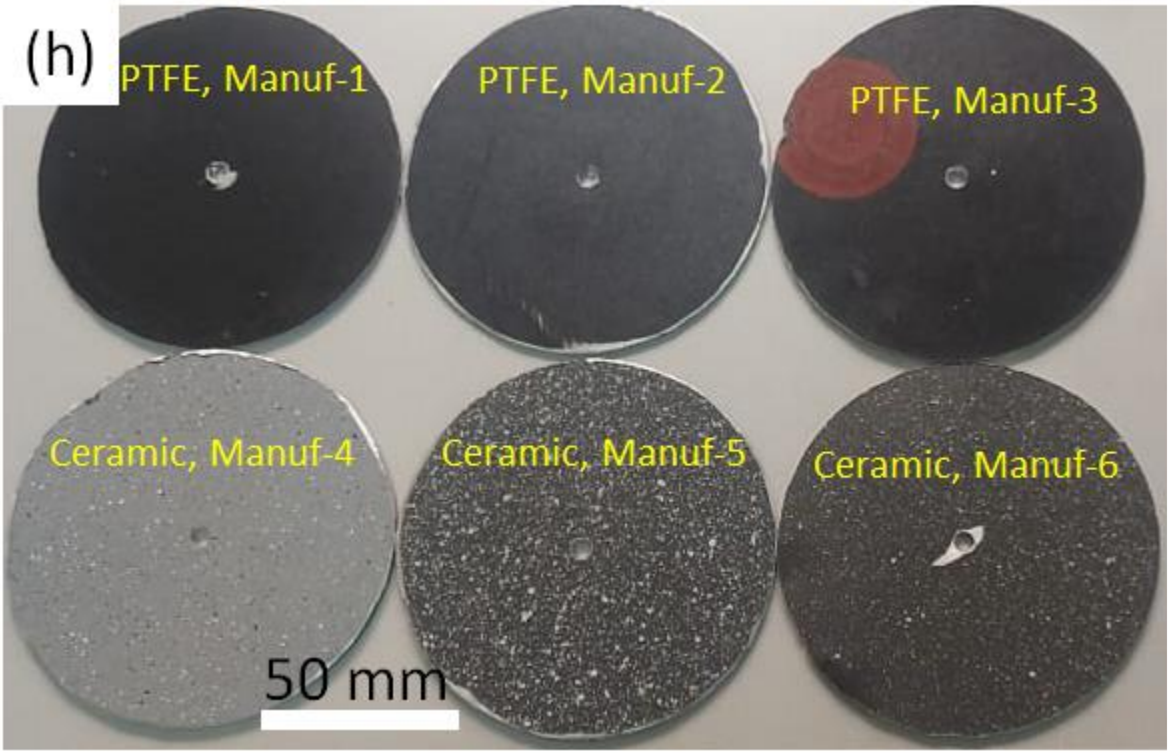

Samples condition: 450°C & 45 min

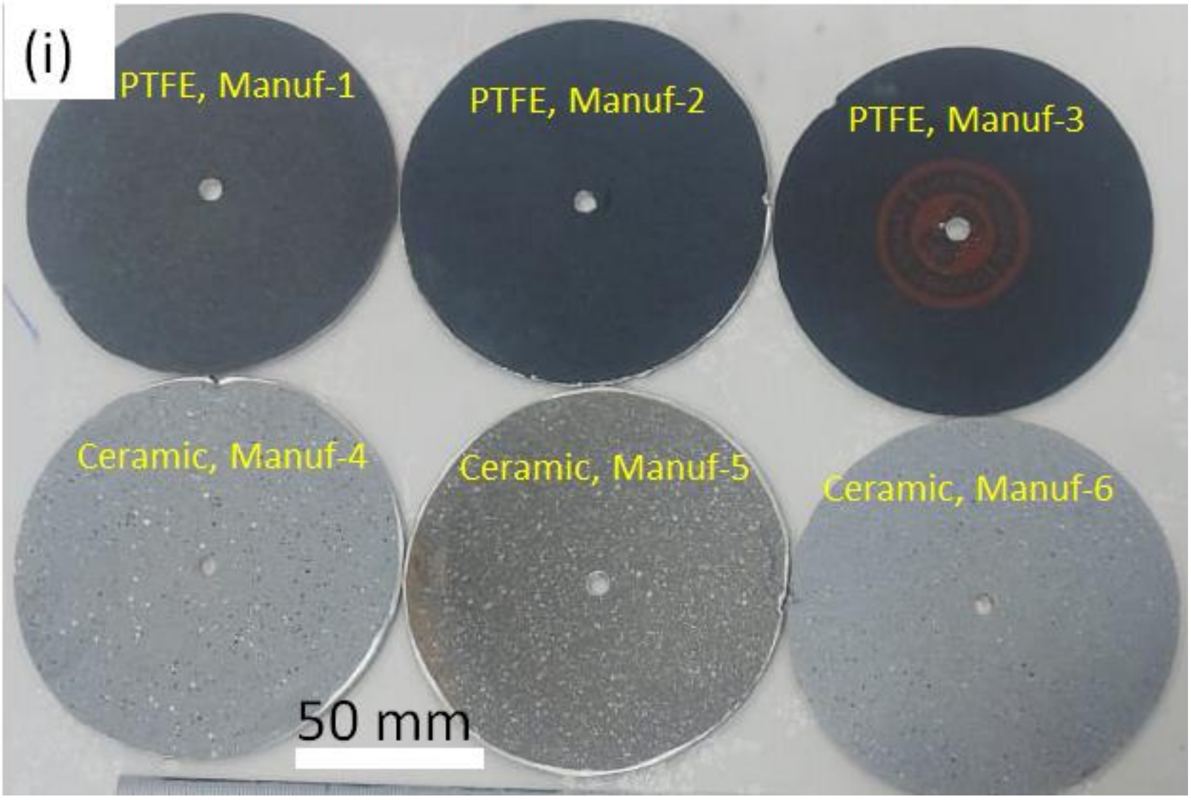

Samples condition: 450°C & 120 min

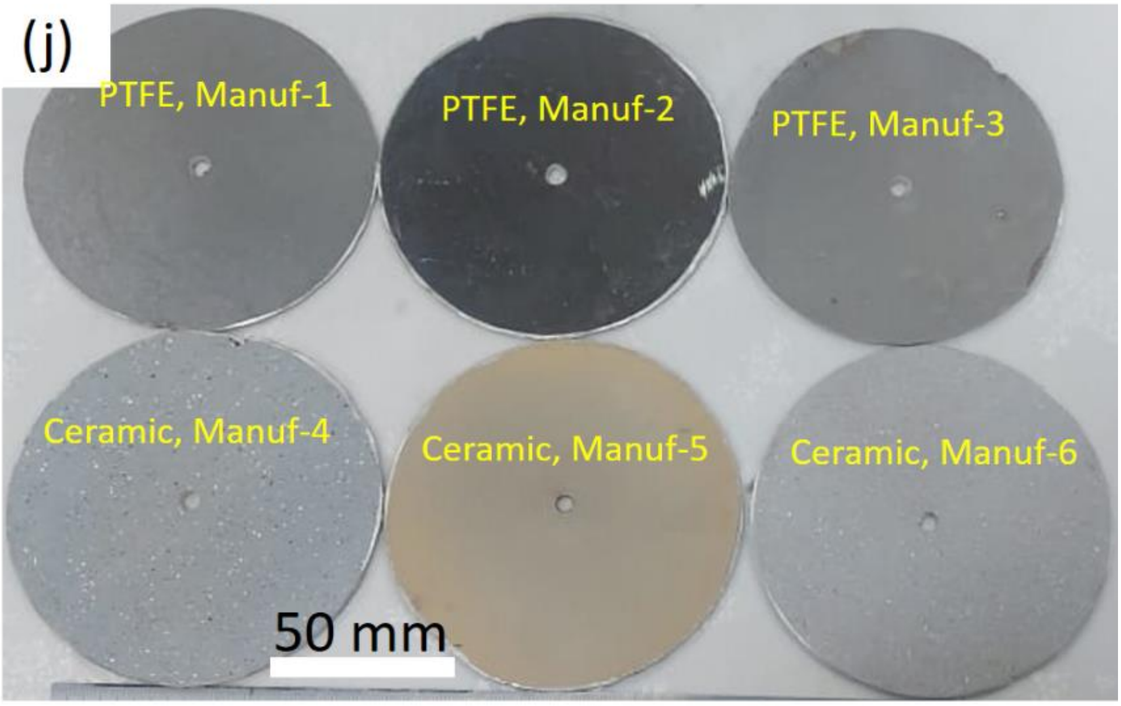

Fig. S5. Photograph of circular shape samples before Taber abrasion test with different conditions: (a) room temperature; (b) 100ºC & 45 min; (c) 100ºC & 120 min; (d) 175ºC & 45 min; (e) 175ºC & 120 min; (f) 250ºC & 45 min; (g) 250ºC & 120 min; (h) 350ºC & 45 min; (i) 350ºC & 120 min; (j) 450ºC & 45 min; (k) 450ºC & 120 min.

Pull-off adhesion test

Portable pull-off adhesion test is the best method for examining the strength of protective coating materials over the substrate. This test is a uniaxial one as per ASTM: D4541 standard. This method can also be used to find-out the strength of coated materials in civil infrastructures. In this test, the sample surface is to be cleaned, a strong glue is to be applied over certain area on the sample, then a dolly (20 mm in diameter) is to be pressed over the substrate, leave the dolly and sample together for curing (at least one day). Next, portable pull-off adhesion tester is to be used for exerting a perpendicular force which remove the dolly along with coated materials from the substrate. A force at which the coating fails per dolly contact area will give adhesive strength. Various factors would affect the performance of pull-off adhesion test, namely, the mixing of epoxy glue, types of glue, sample surface preparation method (either by applying acetone/ethanol or ultrasonic cleaning), the shape and size of dolly, test temperature (room and elevated one), and curing time. Usually, two pack of epoxy (Part-A and Part-B) is to be used during the adhesion test. The Part-A is resin and the part-B is a hardener which are to be mixed in equal manner (approximately 1:1).

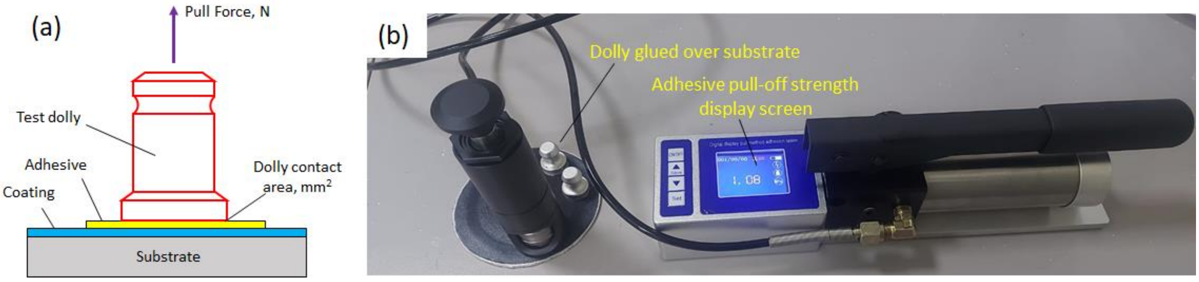

Fig S6. (a) Schematic diagram representing the Pull-Off adhesive test; (b) Pull-Off adhesive tester used during experiment

Fig. S6a shows the schematic diagram indicating the pull-off adhesive test and Fig. S6b shows the photograph of pull-off tester used in this work. Fig S7 show the photograph of new surfaces (after heating) and damaged surfaces (after Taber abrasion test) of samples which were prepared for pull-off adhesive tests from cookware at different heating temperature and time.

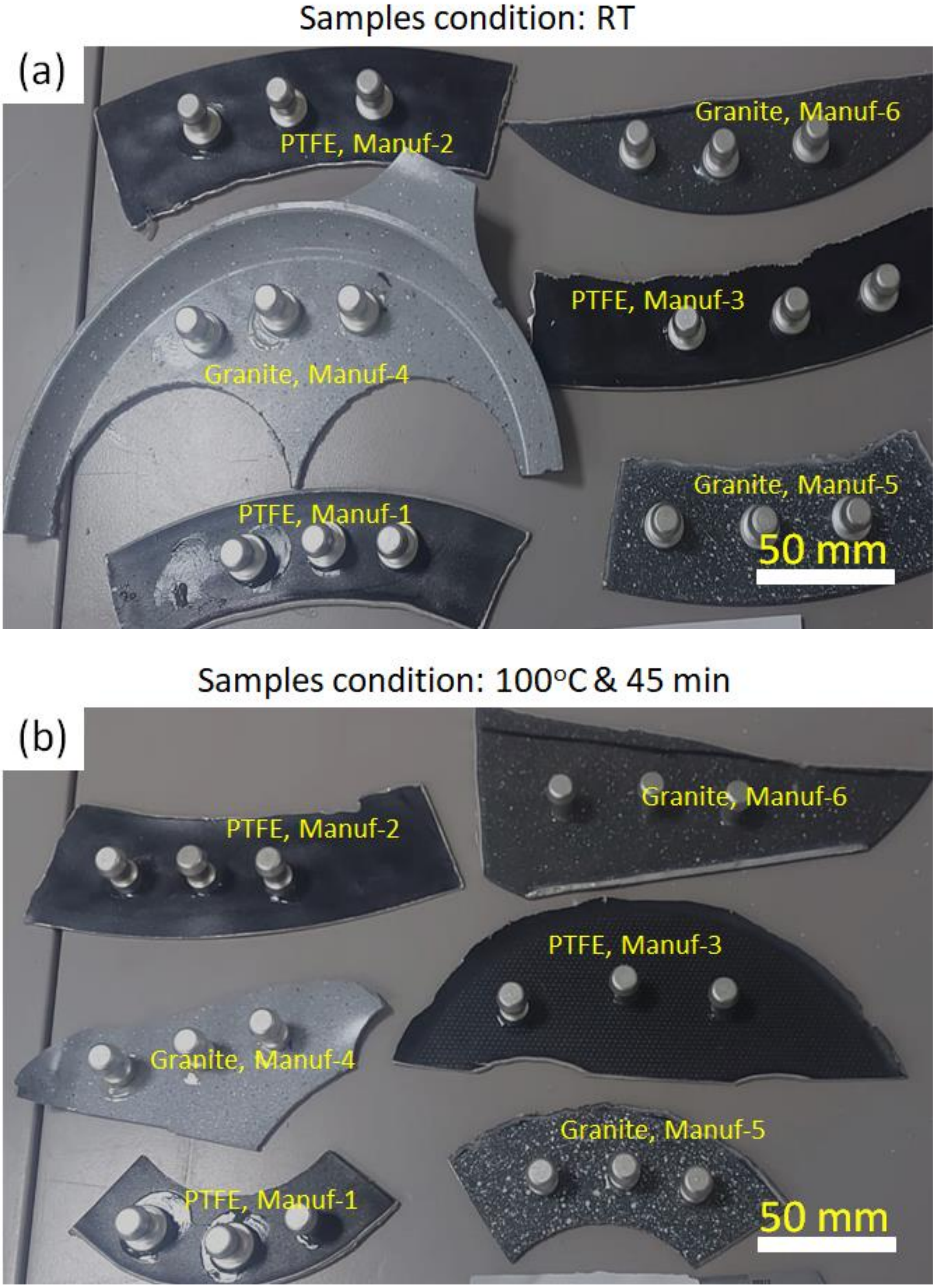
Samples condition: RT
(a)
PTFE, Manuf-2
Granite, Manuf-6
PTFE, Manuf-3
Granite, Manuf-4
Granite, Manuf-5
PTFE, Manuf-1
50 mm
Samples condition: 100°C & 45 min
(b)
Granite, Manuf-6
PTFE, Manuf-2
PTFE, Manuf-3
Granite, Manuf-4
Granite, Manuf-5
PTFE, Manuf-1
50 mm

Samples condition: 175°C & 45 min

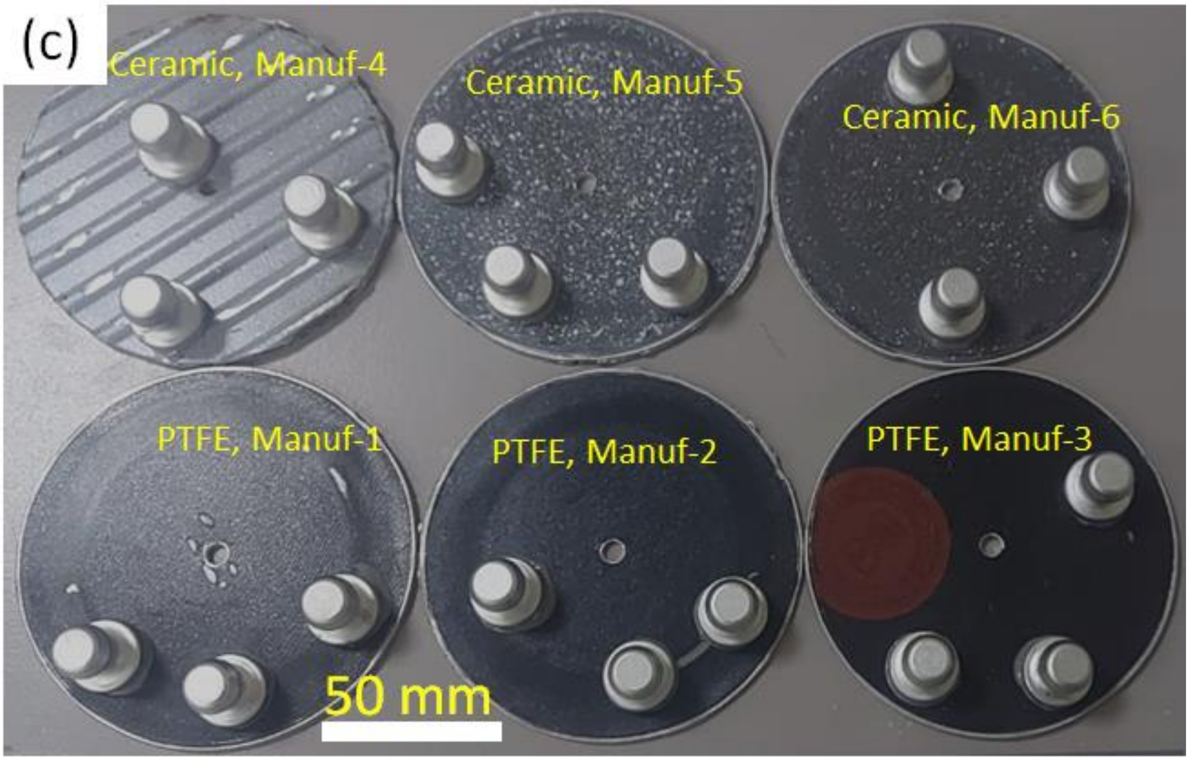

Samples condition: 175°C & 120 min

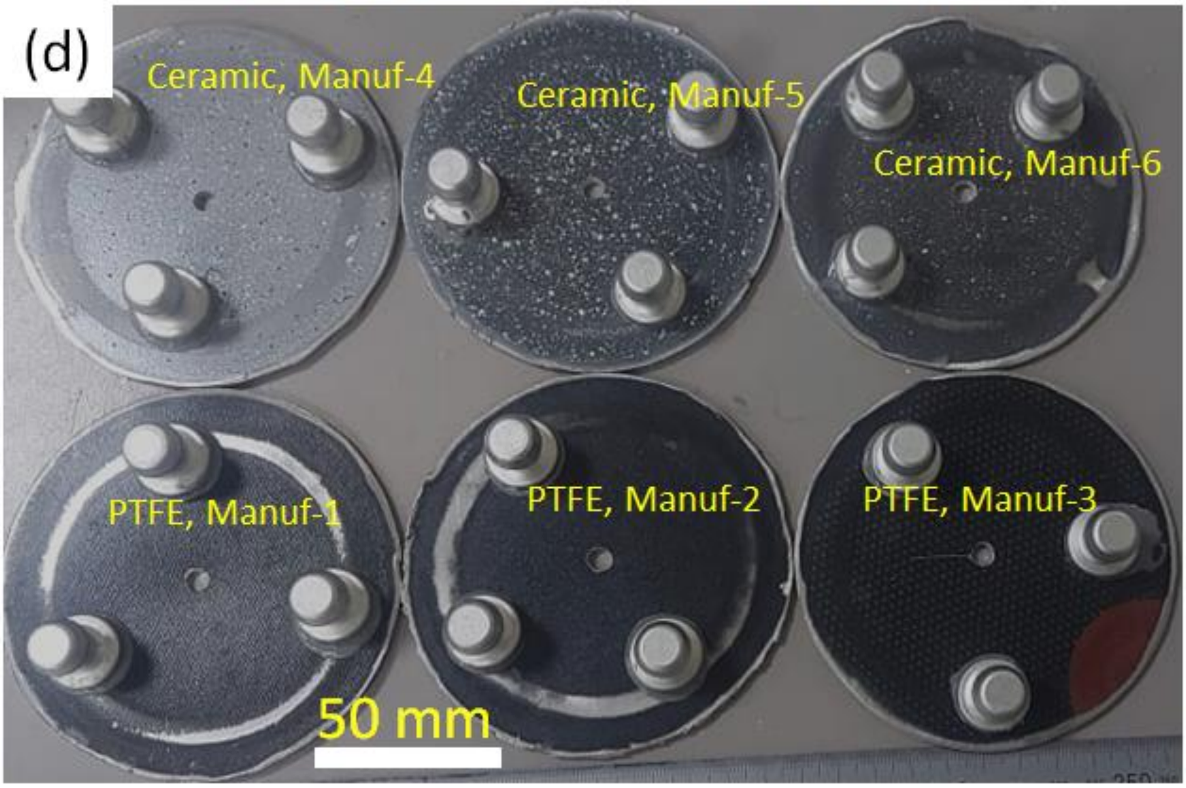

Samples condition: 250°C & 45 min

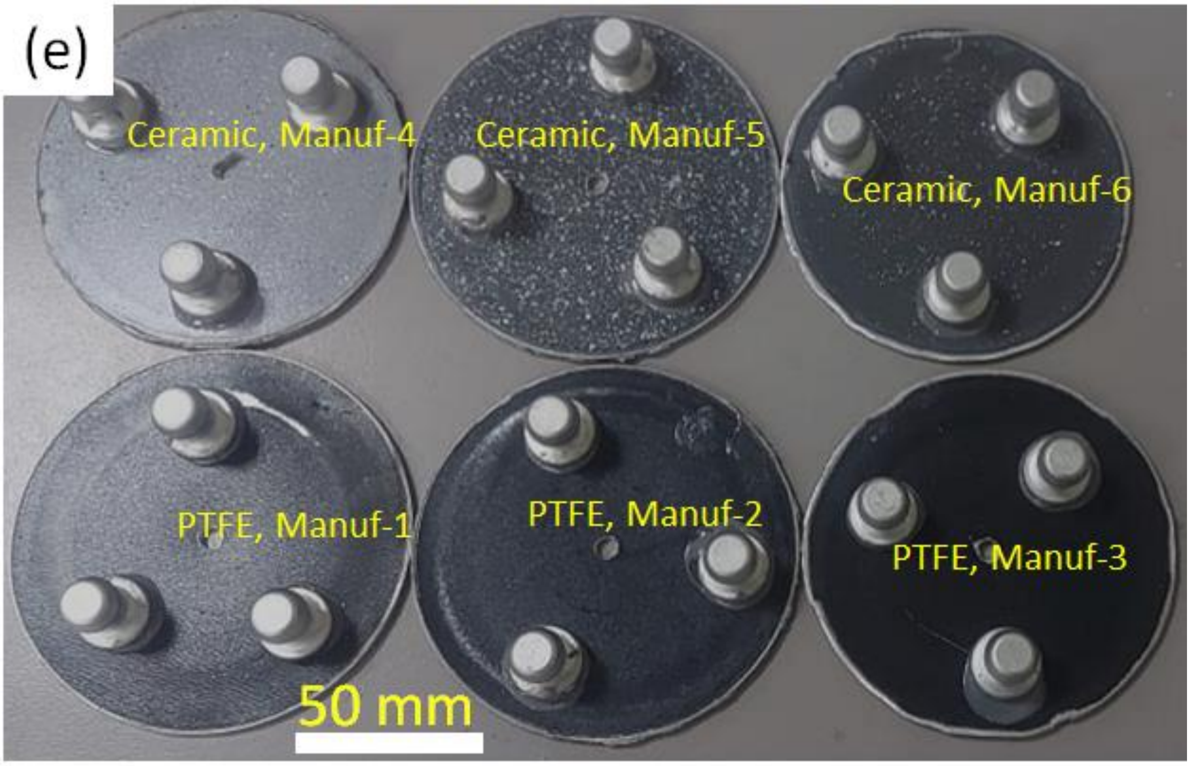

Samples condition: 250°C & 45 min

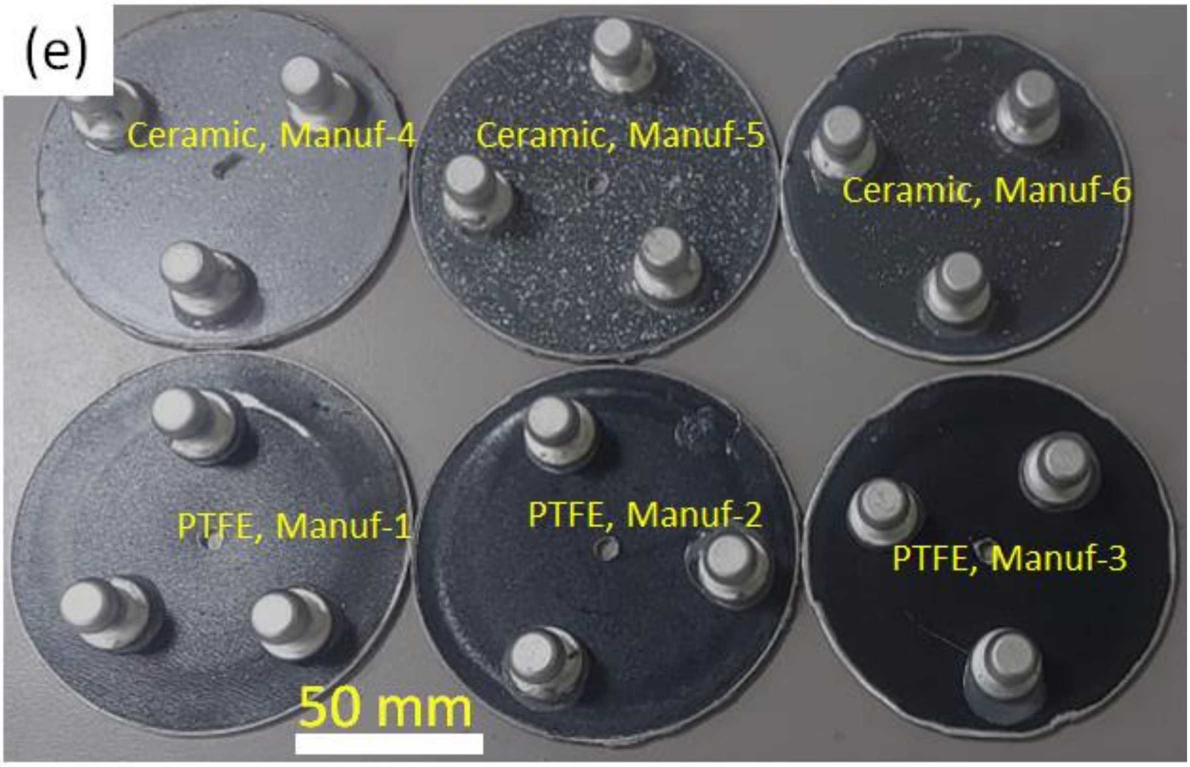

Samples condition: 250°C & 120 min

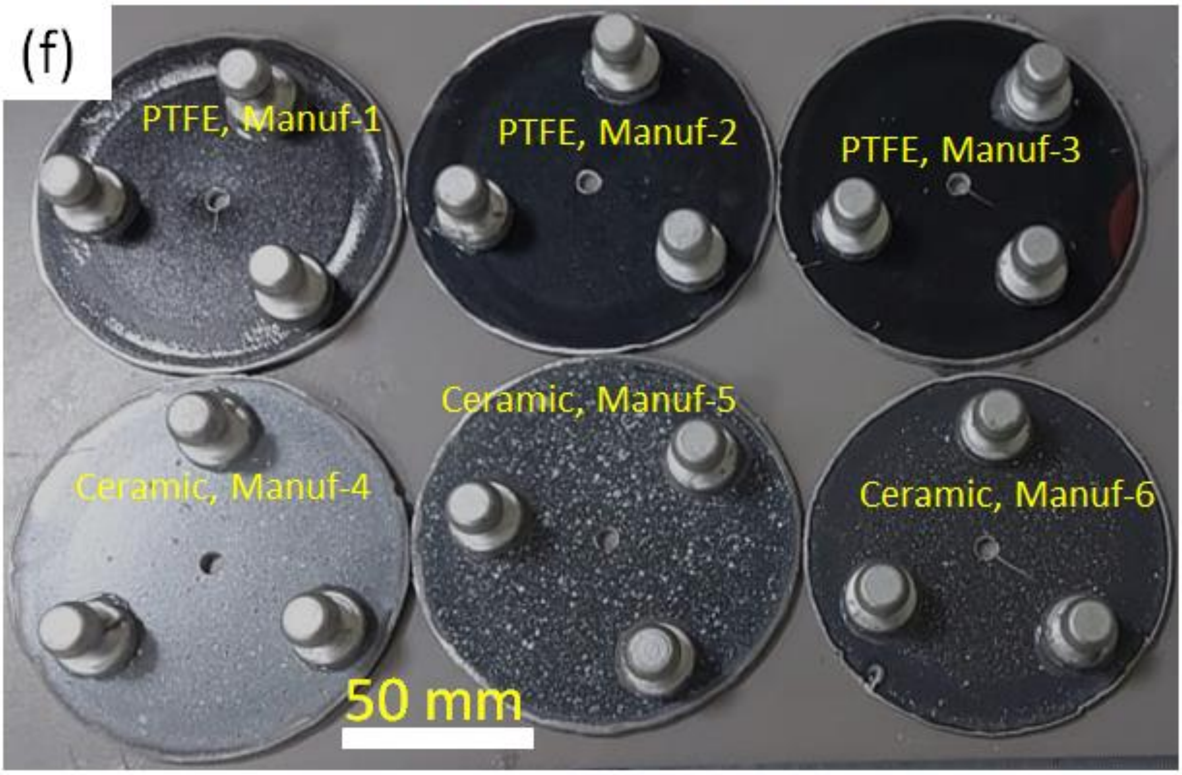

Samples condition: 350°C & 45 min

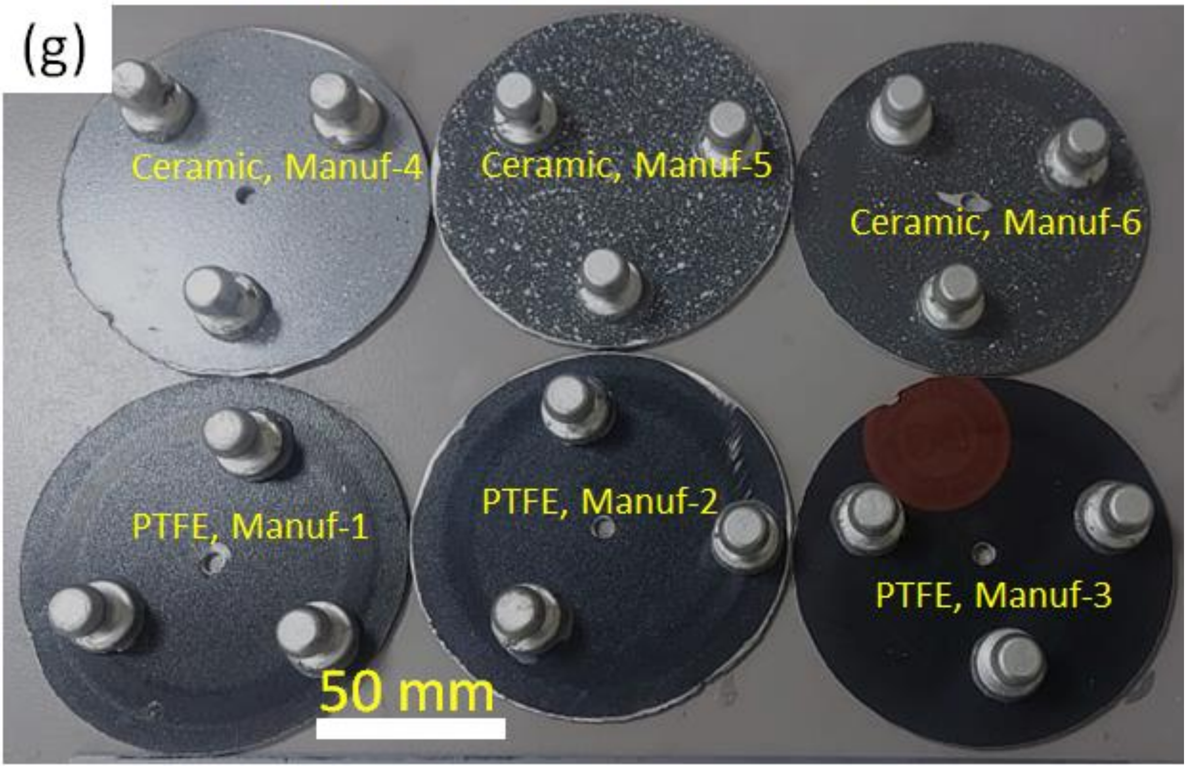

Samples condition: 350°C & 120 min

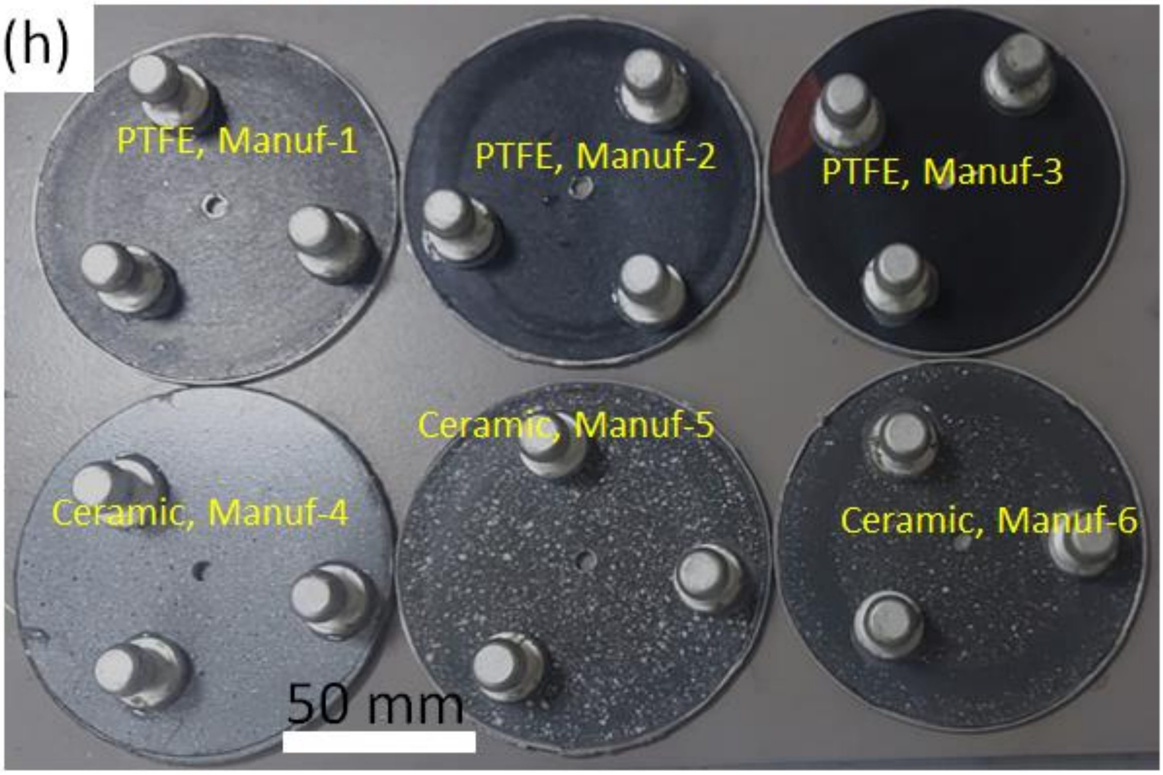

Samples condition: 450°C & 45 min

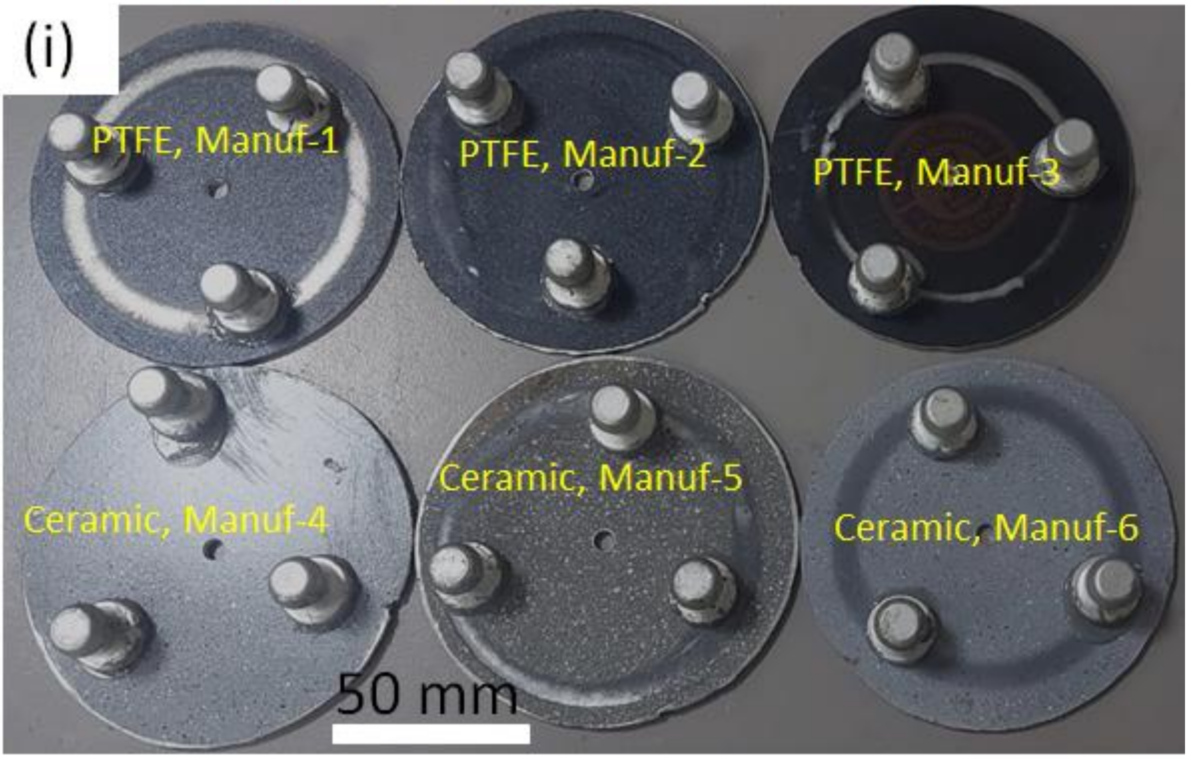

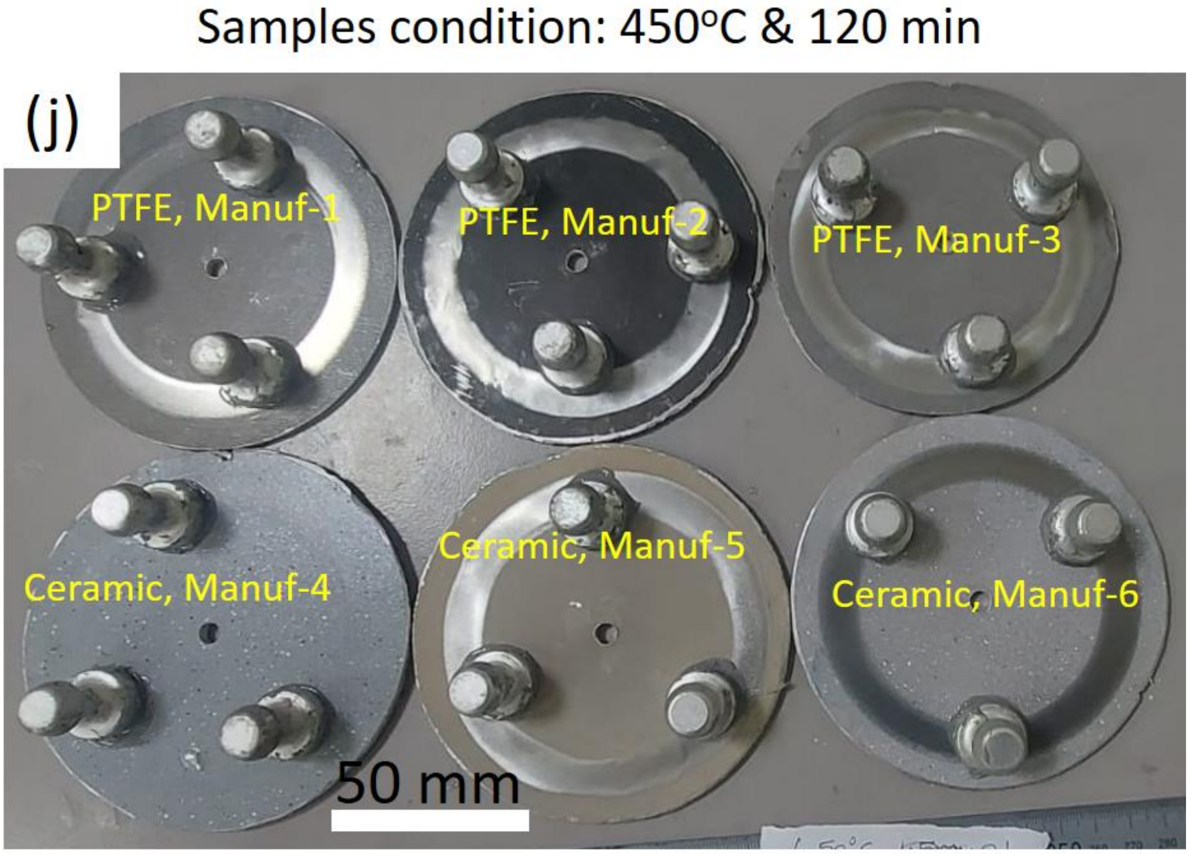

Fig S7. Photograph of heated samples and circular shape samples glued with dollies before Pull-Off adhesive test with different conditions: (a) room temperature; (b) 100ºC & 45 min; (c) 175ºC & 45 min; (d) 175ºC & 120 min; (e) 250ºC & 45 min; (f) 250ºC & 120 min; (g) 350ºC & 45 min; (h) 350ºC & 120 min; (i) 450ºC & 45 min; (j) 450ºC & 120 min.

Corrosion Test:

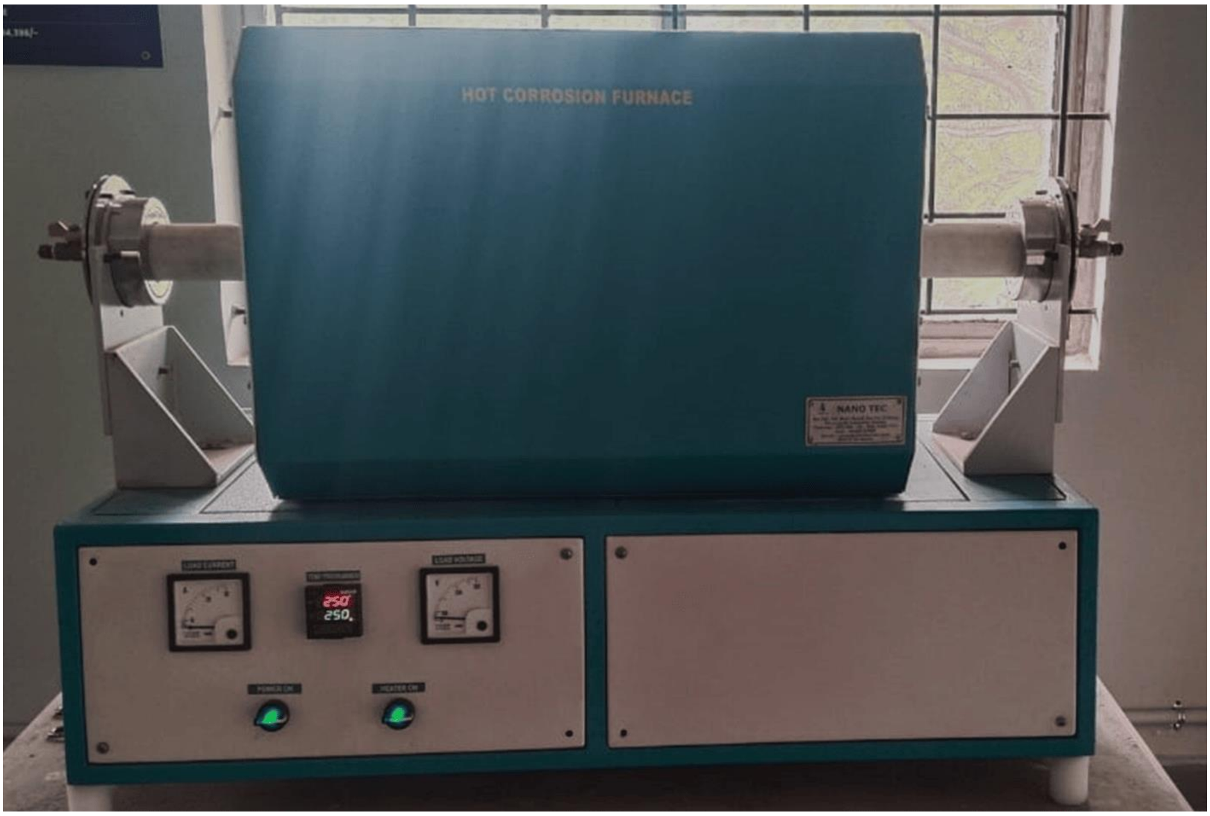

Fig **S8.** Photograph showing the tube furnace used for hot oxidation and corrosion test of cookware used in this study

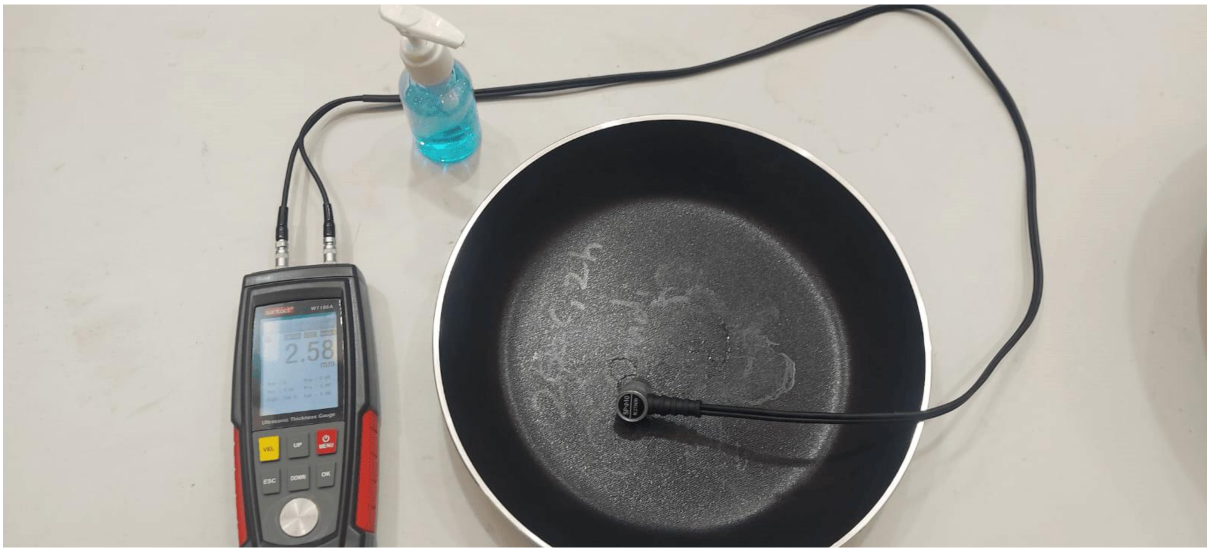

Fig S9. Photograph showing the coating thickness measurement using the ultrasonic method

Taber Wear Test Readings and some Discussion:

Table S1. Variation of average weight loss and Taber wear index at different operating conditions (RT, 100, & 175ºC for 45 & 120 min) measured from Taber rotary abrasion test as per ASTM D4060 standard

Sample Made	Operating Temperature, ºC	Time, min	Average weight loss, g	Taber Wear Index (TWI)

PTFE, Manuf-1	RT	---	0.0196±0.00056	0.0131±0.00012
Granite, Manuf-4	RT	---	0.0035±0.00066	0.0023±0.00037
PTFE, Manuf-2	RT	---	0.0220±0.00037	0.0147±0.00024
Granite, Manuf-5	RT	---	0.0186±0.00079	0.0124±0.00015
PTFE, Manuf-3	RT	---	0.0155±0.00037	0.0103±0.00036
Granite, Manuf-6	RT	---	0.0137±0.00026	0.0091±0.00012
PTFE, Manuf-1	100	45	0.0215±0.00087	0.0143±0.00024
	100	120	0.0226±0.00046	0.0151±0.00026
Granite, Manuf-4	100	45	0.0037±0.00032	0.0025±0.00046
	100	120	0.0039±0.00046	0.0026±0.00024
PTFE, Manuf-2	100	45	0.0242±0.00033	0.0161±0.00012
	100	120	0.0365±0.00019	0.0243±0.00042
Granite, Manuf-5	100	45	0.0197±0.00033	0.0131±0.00024
	100	120	0.0225±0.00046	0.0150±0.00012
PTFE, Manuf-3	100	45	0.0169±0.00037	0.0113±0.00024
	100	120	0.0183±0.00012	0.0122±0.00037
Granite, Manuf-6	100	45	0.0149±0.00046	0.0099±0.00024
	100	120	0.0163±0.00024	0.0109±0.00037
PTFE, Manuf-1	175	45	0.0236±0.00037	0.0157±0.00026
	175	120	0.0266±0.00024	0.0177±0.00037
Granite, Manuf-4	175	45	0.0039±0.00012	0.0026±0.00037
	175	120	0.0040±0.00033	0.0027±0.00026
PTFE, Manuf-2	175	45	0.0307±0.00024	0.0205±0.00037
	175	120	0.0573±0.00046	0.0382±0.00026
Granite, Manuf-5	175	45	0.0248±0.00046	0.0165±0.00026
	175	120	0.0284±0.00024	0.0189±0.00024
PTFE, Manuf-3	175	45	0.0177±0.00014	0.0118±0.00015
	175	120	0.0199±0.00033	0.0132±0.00024
Granite, Manuf-6	175	45	0.0168±0.00046	0.0112±0.00037
	175	120	0.0190±0.00046	0.0126±0.00036

Table S2. Variation of average weight loss and Taber wear index at different operating conditions (250, 350 & 455ºC for 45 & 120 min) measured from Taber rotary abrasion test as per ASTM D4060 standard

Sample Made	Operating Temperature, ºC	Time, min	Average weight loss, g	Taber Wear Index (TWI)
PTFE, Manuf-1	250	45	0.0247±0.00033	0.0165±0.00033
	250	120	0.0272±0.00024	0.0181±0.000124
Granite, Manuf-4	250	45	0.0043±0.00019	0.0028±0.00015

	250	120	0.0045±0.00033	0.0030±0.00023
PTFE, Manuf-2	250	45	0.0381±0.00040	0.0254±0.00024
	250	120	0.0615±0.00037	0.0410±0.00025
Granite, Manuf-5	250	45	0.0271±0.00024	0.0181±0.00016
	250	120	0.0314±0.00015	0.0209±0.00015
PTFE, Manuf-3	250	45	0.0197±0.00024	0.0131±0.00020
	250	120	0.0237±0.00086	0.0158±0.00024
Granite, Manuf-6	250	45	0.0184±0.00024	0.0123±0.00015
	250	120	0.0214±0.00015	0.0142±0.00025
PTFE, Manuf-1	350	45	0.0264±0.00033	0.0176±0.00032
	350	120	0.0301±0.00024	0.0201±0.00019
Granite, Manuf-4	350	45	0.0046±0.00012	0.0031±0.00021
	350	120	0.0051±0.00024	0.0034±0.0004
PTFE, Manuf-2	350	45	0.0402±0.00015	0.0268±0.00031
	350	120	0.0821±0.00024	0.0547±0.00027
Granite, Manuf-5	350	45	0.0297±0.00037	0.0198±0.00018
	350	120	0.0333±0.00012	0.0222±0.00019
PTFE, Manuf-3	350	45	0.0221±0.00037	0.0148±0.00022
	350	120	0.0281±0.00024	0.0187±0.00024
Granite, Manuf-6	350	45	0.0203±0.00024	0.0135±0.00025
	350	120	0.0262±0.00037	0.0175±0.00027
PTFE, Manuf-1	450	45	0.0355±0.00048	0.0237±0.00024
	450	120	0.0452±0.00040	0.0301±0.00024
Granite, Manuf-4	450	45	0.0049±0.00015	0.0033±0.00031
	450	120	0.0075±0.00033	0.0050±0.00024
PTFE, Manuf-2	450	45	0.0631±0.00024	0.0421±0.00027
	450	120	0.1027±0.00015	0.0685±0.00037
Granite, Manuf-5	450	45	0.0385±0.00024	0.0256±0.00024
	450	120	0.0586±0.00046	0.0391±0.0007
PTFE, Manuf-3	450	45	0.0309±0.00037	0.0206±0.00024
	450	120	0.0421±0.00024	0.0281±0.00037
Granite, Manuf-6	450	45	0.0240±0.00046	0.0160±0.00024
	450	120	0.0369±0.00065	0.0246±0.00040

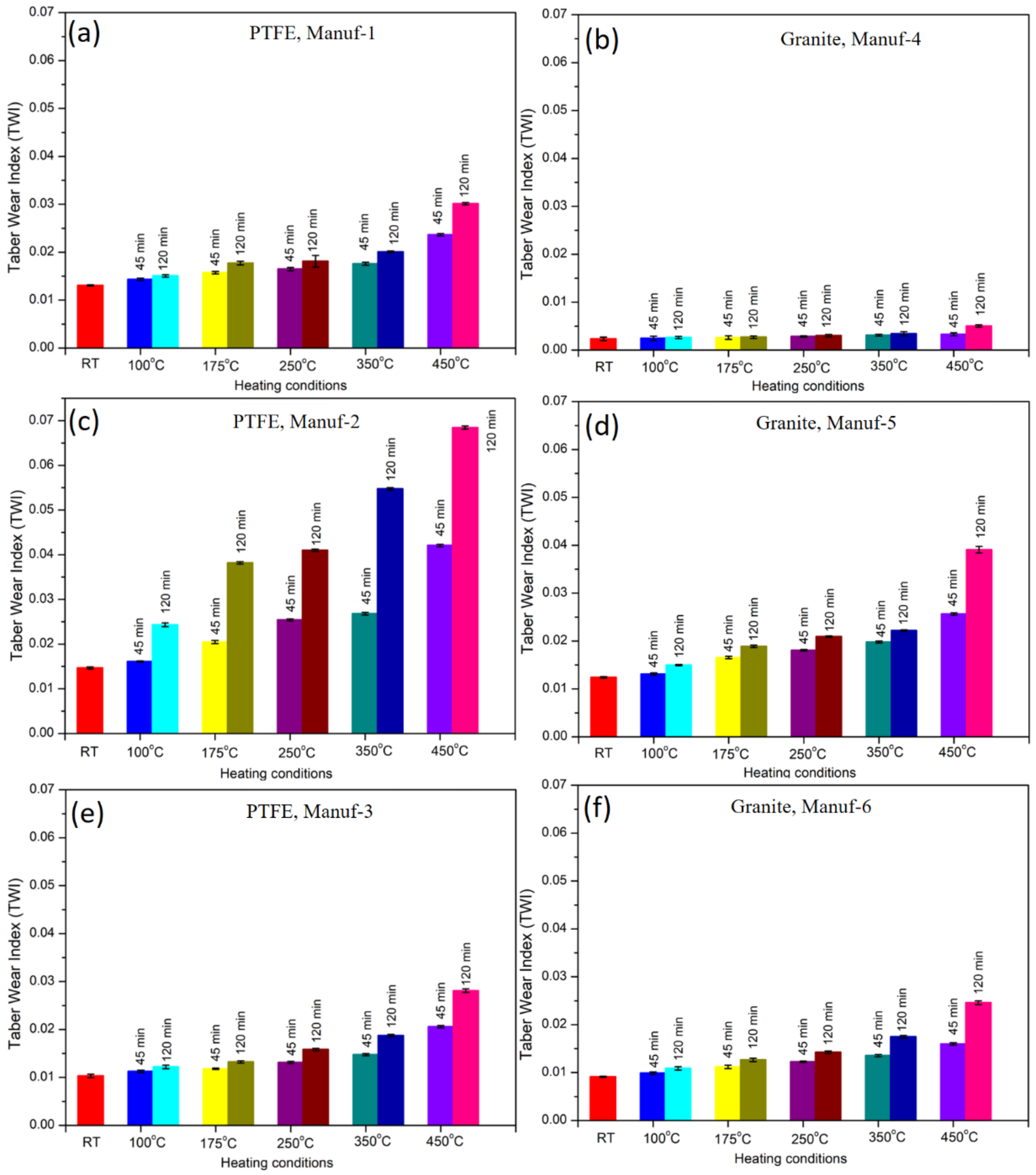

Fig S10. Variation of Taber wear index (TWI) obtained from Taber rotary abrasion wear test for all cookware heated with different conditions (RT, 100, 175, 250, 350, & 450°C for 45 & 120 min): (a) PTFE Manuf-1; (b) Granite Manuf-4; (c) PTFE Manuf-2; (d) Granite Manuf-5; (e) PTFE Manuf-3; and (f) Granite Manuf-6

The weight loss of Manuf-2 pan at 45 min heating exhibited 0.0220, 0.0242, 0.0307, 0.0381, 0.0402, and 0.0631g for RT, 100, 175, 250, 350, and 450°C, respectively. The percentage of variation of weight loss as a function of temperature compared to the RT sample was 9.82%, 39.54%, 73.25%, 82.82%, and 186.6% for 100, 175, 250, 350, and 450°C, respectively. The weight loss of Manuf-3 pan at 120 min heating was 0.0220, 0.0365, 0.0573, 0.0615, 0.0821, and 0.1027g for RT, 100, 175, 250, 350, and 450°C, respectively. In terms of percentage of weight loss, PTFE Manuf-3 pan after 120 min heating compared to RT sample was 65.39%, 160.3%, 179.5%, 273.3%, and 366.7% for 100, 175, 250, 350, and 450°C, respectively. The weight loss of PTFE Manuf-3 pan after 45 min heating was 0.0155, 0.0169, 0.0177, 0.0197, 0.0221, and 0.0309 g for RT, 100, 175, 250, 350, and 450°C, respectively. The weight loss variation percentage compared to the RT sample was 9.03%, 14.06%, 27.1%, 42.78%, and 99.25% for 100, 175, 250, 350, and 450°C, respectively. Similar manner, the weight loss of PTFE Manuf-3 pan after 120 min heating was 0.0155, 0.0183, 0.0199, 0.0237, 0.0281, and 0.0421g for RT, 100, 175, 250, 350, and 450°C, respectively. In terms of percentage of weight loss compared to the RT

sample, PTFE Manuf-3 pan exhibited 18.0%, 28.16%, 53.0%, 81.29%, and 171.9% for 100, 175, 250, 350, and 450°C, respectively.

The weight loss of Granite Manuf-5 pan at 45 min heating exhibited 0.0186, 0.0197, 0.0248, 0.0271, 0.0297, and 0.0385g for RT, 100, 175, 250, 350, and 450°C, respectively. The percentage of weight loss variation of Granite Manuf-5 pan was 5.69%, 33.33%, 56.7%, 59.62%, and 106.7% for 100, 175, 250, 350, and 450°C, respectively. Similar manner, the weight loss of Granite Manuf-5 pan after 120 min heating was 0.0186, 0.0225, 0.0284, 0.0314, 0.0333, and 0.0586g for RT, 100, 175, 250, 350, and 450°C, respectively. The percentage of weight loss variation compared to RT cookware was 20.73%, 52.47%, 68.81%, 78.84%, and 215.04% for 100, 175, 250, 350, and 450°C, respectively. The weight loss of Granite Manuf-6 pan after 45 min heating exhibited 0.0137, 0.0149, 0.0168, 0.0184, 0.0203, and 0.0240g for RT, 100, 175, 250, 350, and 450°C, respectively. The percentage of weight loss variation compared to the RT sample was 8.86%, 22.96%, 34.74%, 48.84%, and 75.82% for 100, 175, 250, 350, and 450°C, respectively. Similarly, the weight loss of Granite Manuf-6 non-stick cookware at 120 min heating was 0.0137, 0.0163, 0.0190, 0.0214, 0.0262, and 0.0369 g for RT, 100, 175, 250, 350, and 450°C, respectively. The percentage of weight loss variation compared to the RT sample was 19.44%, 38.95%, 56.53%, 91.94%, and 170.11% for 100, 175, 250, 350, and 450°C, respectively.

Adhesive Pull-off test readings and some Discussions:

Table S3. Variation of Pull-off adhesive strength obtained from undamaged and damaged surfaces after Taber abrasion test at different operating conditions (RT, 100, & 175ºC for 45 & 120 min) as per ASTM D4541 standard

Sample Made	Operating Temperature, ºC	Time, min	Average Pull-off adhesive strength, MPa
PTFE, Manuf-1	RT	---	0.7437±0.0553
Granite, Manuf-4	RT	---	0.7864±0.0389
PTFE, Manuf-2	RT	---	0.9774±0.0456
Granite, Manuf-5	RT	---	1.0230±0.0278
PTFE, Manuf-3	RT	---	0.8699±0.0689
Granite, Manuf-6	RT	---	0.9863±0.0546
PTFE, Manuf-1	100	45	0.7632±0.0325
	100	120	0.8965±0.0412
Granite, Manuf-4	100	45	0.9837±0.0214
	100	120	1.1235±0.0402
PTFE, Manuf-2	100	45	1.0325±0.0123
	100	120	1.1456±0.0302
Granite, Manuf-5	100	45	1.3025±0.0757
	100	120	1.4237±0.0236
PTFE, Manuf-3	100	45	0.8936±0.0347
	100	120	1.1154±0.0247
Granite, Manuf-6	100	45	1.2365±0.0896
	100	120	1.4699±0.0757
PTFE, Manuf-1	175	45	0.8237±0.0314
	175	120	0.9365±0.0247

Granite, Manuf-4	175	45	1.1237±0.0347
	175	120	1.3215±0.0245
PTFE, Manuf-2	175	45	1.1456±0.0502
	175	120	1.2456±0.0489
Granite, Manuf-5	175	45	1.3897±0.0364
	175	120	1.5536±0.0189
PTFE, Manuf-3	175	45	0.9237±0.0307
	175	120	1.2347±0.0278
Granite, Manuf-6	175	45	1.3564±0.0658
	175	120	1.5789±0.0763

Table S4. Variation of Pull-off adhesive strength obtained from undamaged and damaged surfaces after Taber abrasion test at different operating conditions (250, 350 & 450ºC for 45 & 120 min) as per ASTM D4541 standard

Sample Made	Operating Temperature, ºC	Time, min	Average weight loss, g
PTFE, Manuf-1	250	45	1.0236±0.0323
	250	120	1.0456±0.0389
Granite, Manuf-4	250	45	1.1898±0.0245
	250	120	1.3569±0.0423
PTFE, Manuf-2	250	45	1.2365±0.0378
	250	120	1.3256±0.0578
Granite, Manuf-5	250	45	1.5236±0.0168
	250	120	1.6589±0.0378
PTFE, Manuf-3	250	45	1.3256±0.0865
	250	120	1.4896±0.0654
Granite, Manuf-6	250	45	1.5690±0.0789
	250	120	1.7896±0.0365
PTFE, Manuf-1	350	45	1.2365±0.0632
	350	120	1.3256±0.0345
Granite, Manuf-4	350	45	1.3896±0.0702
	350	120	1.5893±0.0305
PTFE, Manuf-2	350	45	1.4699±0.0523
	350	120	1.5896±0.0436
Granite, Manuf-5	350	45	1.6897±0.0365
	350	120	1.7366±0.0896
PTFE, Manuf-3	350	45	1.4699±0.0658
	350	120	1.5366±0.0432
Granite, Manuf-6	350	45	1.8966±0.0389
	350	120	2.0237±0.0647

PTFE, Manuf-1	450	45	1.3589±0.0289
	450	120	1.4898±0.0456
Granite, Manuf-4	450	45	2.1230±0.0698
	450	120	2.8965±0.0324
PTFE, Manuf-2	450	45	1.8965±0.0345
	450	120	1.9659±0.0248
Granite, Manuf-5	450	45	2.2365±0.0569
	450	120	2.6987±0.0324
PTFE, Manuf-3	450	45	1.6659±0.0456
	450	120	1.7897±0.0268
Granite, Manuf-6	450	45	2.2057±0.0654
	450	120	2.5632±0.0456

The adhesive pull-off strength of PTFE Manuf-2 pan at 45 min heating exhibited 0.9774, 1.0325, 1.1456, 1.2365, 1.4699, and 1.8965 MPa for RT, 100, 175, 250, 350, and 450°C, respectively. The percentage of variation of adhesive pull-off strength as a function of temperature compared to the RT sample was 5.64%, 17.21%, 26.51%, 50.39%, and 94.04% for 100, 175, 250, 350, and 450°C, respectively. Here, PTFE Manuf-2 pan adhesive pull-off strength exhibited more than the PTFE Manuf-1 sample, meaning that more loss of C-F atoms and more damage after the Taber wear test occurred in these cookware's. The adhesive pull-off strength of PTFE Manuf-2 pan at 120 min heating was 0.9774, 1.1456, 1.2456, 1.3256, 1.5896, and 1.9659 MPa for RT, 100, 175, 250, 350, and 450°C, respectively. In terms of percentage of variation of adhesive pull-off strength, PTFE Manuf-3 non-stick cookware after 120 min heating compared to RT sample was 17.21%, 27.44%, 35.63%, 62.64%, and 101.3%, respectively. The adhesive pull-off strength of PTFE Manuf-3 non-stick cookware after 45 min heating was 0.8699, 0.8936, 0.9237, 1.3256, 1.4699, and 1.6659 MPa for RT, 100, 175, 250, 350, and 450°C, respectively. The percentage of variation of adhesive pull-off strength was 2.72%, 6.18%, 52.39%, 68.97%, and 91.57% for 100, 175, 250, 350, and 450°C, respectively. The observed adhesive pull-off strength of PTFE Manuf-3 non-stick cookware was lower than PTFE Manuf-2 cookware and higher than PTFE Manuf-1 non-stick cookware. These results indicate that PTFE Manuf-1 pan exhibited good C-F bonding, followed by France-PTFE and then PTFE Manuf-2 cookware. Similar manner, the adhesive pull-off strength of PTFE Manuf-3 non-stick cookware's after 120 min heating was 0.8699, 1.1154, 1.2347, 1.4896, 1.5366, and 1.7897 MPa for RT, 100, 175, 250, 350, and 450°C, respectively. In terms of variation of percentage of adhesive pull-off strength compared to the RT sample, PTFE Manuf-3 pan non-stick cookware exhibited 28.26%, 41.93%, 71.24%, 76.64%, and 105.7% for 100, 175, 250, 350, and 450°C, respectively. These results demonstrate the poor bonding of C-F atoms in PTFE materials after a long heating of 120 min.

The adhesive pull-off strength of Granite Manuf-4 non-stick cookware after 120 min heating was 0.7864, 1.1235, 1.3215, 1.3569, 1.5893, and 2.8965 MPa for RT, 100, 175, 250, 350, and 450°C, respectively. In terms of percentage of variation, Granite Manuf-4 non-stick cookware compared to the RT sample was 42.88%, 68.04%, 72.55%, 102.11%, and 268.35% for 100, 175, 250, 350, and 450°C, respectively. These results clearly explain that a long heating time (120 min) loosens the silane compound bonding strength over the Al alloy substrate. The adhesive pull-off strength of Granite Manuf-5 non-stick cookware's at 45 min heating exhibited 1.0230, 1.3025, 1.3897, 1.5236, 1.6897, and 2.2365 MPa for RT, 100, 175, 250, 350, and 450°C, respectively. The percentage of variation of

adhesive pull-off strength of Granite Manuf-5 non-stick cookware was 27.32%, 35.84%, 48.93%, 65.17%, and 118.25% for 100, 175, 250, 350, and 450°C, respectively. Here, Granite Manuf-5 non-stick cookware exhibited more adhesive pull-off strength than other suppliers due to the poor strength of silane coating over the substrate. Similar manner, the adhesive pull-off strength of Granite Manuf-5 non-stick cookware's after 120 min heating was 1.0230, 1.4237, 1.5536, 1.6589, 1.7366, and 2.6987 MPa for RT, 100, 175, 250, 350, and 450°C, respectively. The percentage of adhesive pull-off strength variation compared to RT cookware was 39.16%, 51.87%, 62.16%, 69.75%, and 163.80% for 100, 175, 250, 350, and 450°C, respectively. The adhesive pull-off strength of Granite Manuf-6 non-stick cookware after 45 min heating was 0.9863, 1.2365, 1.3564, 1.5690, 1.8966, and 2.2057 MPa for RT, 100, 175, 250, 350, and 450°C, respectively. The percentage of variation of adhesive pull-off strength compared to the RT sample was 25.37%, 37.52%, 59.02%, 92.29%, and 123.63% for 100, 175, 250, 350, and 450°C, respectively. Similarly, the adhesive pull-off strength of Granite Manuf-6 non-stick cookware's at 120 min heating was 0.9863, 1.4699, 1.5789, 1.7896, 2.0237, and 2.5632 MPa for RT, 100, 175, 250, 350, and 450°C, respectively. The percentage of variation of adhesive pull-off strength compared to the RT sample was 49.02%, 60.08%, 81.44%, 105.71%, and 159.88% for 100, 175, 250, 350, and 450°C, respectively.

Surface Roughness values and discussion:

Table S5. Variation of average surface roughness (Ra) value at different operating conditions (RT, 100, & 175ºC for 45 & 120 min) measured from TR 1900 surface roughness tester as per ISO 468 standard

Sample Made	Operating Temperature, ºC	Time, min	Average surface roughness (Ra), µm
PTFE, Manuf-1	RT	---	0.6033±0.0236
Granite, Manuf-4	RT	---	0.4237±0.0488
PTFE, Manuf-2	RT	---	0.7057±0.0659
Granite, Manuf-5	RT	---	1.1237±0.0563
PTFE, Manuf-3	RT	---	0.9137±0.0658
Granite, Manuf-6	RT	---	0.9966±0.0789
PTFE, Manuf-1	100	45	0.6252±0.0309
	100	120	0.6365±0.1740
Granite, Manuf-4	100	45	0.4376±0.0525
	100	120	0.6650±0.1508
PTFE, Manuf-2	100	45	0.7124±0.1876
	100	120	0.7728±0.0815
Granite, Manuf-5	100	45	1.1698±0.0688
	100	120	1.2033±0.1071
PTFE, Manuf-3	100	45	0.9250±0.1285
	100	120	0.9954±0.1529
Granite, Manuf-6	100	45	1.0237±0.1221
	100	120	1.1023±0.1087
PTFE, Manuf-1	175	45	0.6833±0.0667
	175	120	0.7853±0.1062
Granite, Manuf-4	175	45	0.4437±0.0766
	175	120	0.6790±0.0552

PTFE, Manuf-2	175	45	0.8237±0.0801
	175	120	0.8636±0.0739
Granite, Manuf-5	175	45	1.2037±0.0669
	175	120	1.3620±0.0788
PTFE, Manuf-3	175	45	0.9356±0.0707
	175	120	1.0237±0.0773
Granite, Manuf-6	175	45	1.1365±0.1026
	175	120	1.2132±0.1619

Table S6. Variation of average surface roughness (Ra) value at different operating conditions (250, 350, & 450ºC for 45 & 120 min) measured from TR 1900 surface roughness tester as per ISO 468 standard

Sample Made	Operating Temperature, ºC	Time, min	Average surface roughness (Ra), µm
PTFE, Manuf-1	250	45	0.7365±0.0667
	250	120	0.8699±0.1062
Granite, Manuf-4	250	45	0.4563±0.0717
	250	120	0.6898±0.0311
PTFE, Manuf-2	250	45	0.9024±0.0884
	250	120	0.9864±0.0868
Granite, Manuf-5	250	45	1.2456±0.0473
	250	120	1.5363±0.0924
PTFE, Manuf-3	250	45	0.9563±0.1019
	250	120	1.0456±0.1568
Granite, Manuf-6	250	45	1.2456±0.0922
	250	120	1.3589±0.1071
PTFE, Manuf-1	350	45	0.9163±0.0997
	350	120	1.0234±0.0859
Granite, Manuf-4	350	45	0.4654±0.0270
	350	120	0.6987±0.0240
PTFE, Manuf-2	350	45	1.1236±0.0891
	350	120	1.2366±0.0878
Granite, Manuf-5	350	45	1.3023±0.1198
	350	120	1.6282±0.1039
PTFE, Manuf-3	350	45	0.9633±0.0354
	350	120	1.1237±0.1672
Granite, Manuf-6	350	45	1.2870±0.0881
	350	120	1.4652±0.1055
PTFE, Manuf-1	450	45	1.0237±0.0499

	450	120	1.1365±0.0783
Granite, Manuf-4	450	45	0.4966±0.0301
	450	120	0.7237±0.0745
PTFE, Manuf-2	450	45	1.2456±0.1651
	450	120	1.3652±0.1444
Granite, Manuf-5	450	45	1.4023±0.1158
	450	120	1.8253±0.0473
PTFE, Manuf-3	450	45	0.9837±0.1029
	450	120	1.2037±0.0328
Granite, Manuf-6	450	45	1.3021±0.1023
	450	120	1.5237±0.1002

The value of surface roughness of PTFE Manuf-1 non-stick cookware after heating 45 min exhibited 0.6033, 0.6252, 0.6833, 0.7365, 0.9163, and 1.0237 µm for RT, 100, 175, 250, 350, and 450°C, respectively. The variation in the percentage of surface roughness value of PTFE Manuf-1 non-stick cookware compared to RT was 3.63%, 13.26%, 22.09%, 51.89%, and 69.68% for 100, 175, 250, 350, and 450°C, respectively. These results demonstrate low surface roughness variation up to 250°C. However, beyond 250°C, the variation of surface roughness value was increased drastically due to the weak bonding of C-F atoms over the substrate. Similarly, after heating at 120 min, the surface roughness value of PTFE Manuf-1 pan was 0.6033, 0.6365, 0.7853, 0.8699, 1.0234, and 1.1365 µm for RT, 100, 175, 250, 350, and 450°C, respectively. The variation of the percentage of surface roughness value as a function of temperature compared to the RT sample was 5.51%, 30.18%, 44.19%, 69.64%, and 88.60% for 100, 175, 250, 350, and 450°C, respectively. The surface roughness value of PTFE Manuf-2 pan at 45 min heating exhibited 0.7057, 0.7124, 0.8237, 0.9024, 1.1236, and 1.2456 µm for RT, 100, 175, 250, 350, and 450°C, respectively. The percentage of variation of surface roughness value as a function of temperature compared to the RT sample was 0.944%, 16.714%, 27.86%, 59.21%, and 76.51% for 100, 175, 250, 350, and 450°C, respectively. The surface roughness value of PTFE Manuf-2 non-stick cookware at 120 min heating was 0.7057, 0.7728, 0.8636, 0.9864, 1.2366, and 1.3652 µm for RT, 100, 175, 250, 350, and 450°C, respectively. In terms of percentage of variation of surface roughness value, PTFE Manuf-3 non-stick cookware after 120 min heating compared to RT sample was 9.50%, 22.37%, 39.77%, 75.22%, and 93.45%, respectively. The surface roughness value of PTFE Manuf-3 non-stick cookware after 45 min heating was 0.9137, 0.9250, 0.9356, 0.9563, 0.9633, and 0.9837 mm for RT, 100, 175, 250, 350, and 450°C, respectively. The percentage of variation of surface roughness value was 1.24%, 2.40%, 4.66%, 5.42%, and 7.66% for 100, 175, 250, 350, and 450°C, respectively. The observed variation of surface roughness value of PTFE Manuf-3 non-stick cookware was very low due to the strong bonding of C-F atoms and effective concentration of PTFE materials over the substrate. Similar manner, the surface roughness value of PTFE Manuf-3 non-stick cookware's after 120 min heating was 0.9137, 0.9954, 1.0237, 1.0456, 1.1237, and 1.2037 µm for RT, 100, 175, 250, 350, and 450°C, respectively. In terms of variation of percentage of surface roughness value compared to the RT sample, PTFE Manuf-3 non-stick cookware exhibited 8.94%, 12.03%, 14.44%, 22.98%, and 31.74% for 100, 175, 250, 350, and 450°C, respectively. These results demonstrate the poor bonding of C-F atoms in PTFE materials after a long heating of 120 min.

For ceramic Granite coated cookware, the surface roughness value of Granite Manuf-4 non-stick cookware after 45 min heating exhibited 0.4237, 0.4376, 0.4437, 0.4563, 0.4654, and 0.4966 µm for RT, 100, 175, 250, 350, and 450°C, respectively. The percentage of variation of surface roughness value compared to the RT sample was 3.29%, 4.71%, 7.71%, 9.84%, and 17.21% for 100, 175, 250, 350, and

450°C, respectively. Here, the variation of surface roughness value with the function of temperature was very low. This was expected due to the effective concentration of silane materials over the substrate. The surface roughness value of Granite Manuf-4 non-stick cookware after 120 min heating was 0.4237, 0.6650, 0.6790, 0.6898, 0.6987, and 0.7237 µm for RT, 100, 175, 250, 350, and 450°C, respectively. In terms of percentage of variation, Granite Manuf-4 non-stick cookware compared to the RT sample was 56.96%, 60.26%, 62.80%, 64.93%, and 70.80% for 100, 175, 250, 350, and 450°C, respectively. These results clearly explain to us that a long heating time (120 min) loosens the strength of the silane compound over the substrate. The surface roughness value of Granite Manuf-5 non-stick cookware's at 45 min heating exhibited 1.1237, 1.1698, 1.2037, 1.2456, 1.3023, and 1.4023 µm for RT, 100, 175, 250, 350, and 450°C, respectively. The percentage of variation of surface roughness value of Granite Manuf-5 non-stick cookware was 4.10%, 7.11%, 10.85%, 15.89%, and 24.97% for 100, 175, 250, 350, and 450°C, respectively. Here, Granite Manuf-5 non-stick cookware exhibited more surface roughness value than other suppliers due to the poor strength of silane compound coating over the substrate. Similar manner, the surface roughness value of Granite Manuf-5 non-stick cookware's after 120 min heating was 1.1237, 1.2033, 1.3620, 1.5363, 1.6282, and 1.8253 mm for RT, 100, 175, 250, 350, and 450°C, respectively. The variation in the percentage of surface roughness value compared to RT cookware was 7.09%, 21.21%, 36.72%, 44.89%, and 62.44% for 100, 175, 250, 350, and 450°C, respectively. The surface roughness value of Granite Manuf-6 non-stick cookware after 45 min heating was 0.9966, 1.0237, 1.1365, 1.2456, 1.2870, and 1.3021 mm for RT, 100, 175, 250, 350, and 450°C, respectively. The percentage of variation of surface roughness value compared to the RT sample was 2.71%, 14.03%, 24.98%, 29.13%, and 30.65% for 100, 175, 250, 350, and 450°C, respectively. Similarly, the surface roughness value of Granite Manuf-6 non-stick cookware at 120 min heating was 0.9966, 1.1023, 1.2132, 1.3589, 1.4652, and 1.5237 µm for RT, 100, 175, 250, 350, and 450°C, respectively. The percentage of variation of surface roughness value compared to the RT sample was 10.6%, 21.73%, 36.35%, 47.02%, and 52.88% for 100, 175, 250, 350, and 450°C, respectively. These results indicate that the bonding strength of C-F atoms in PTFE non-stick cookware and silane bonding materials in Ceramic non-stick cookware decreased drastically with increasing temperature and time, especially after 120 min long heating. Among the investigated non-stick cookware, PTFE Manuf-1, PTFE Manuf-3, and Granite Manuf-4 up to 250°C retained coating materials over Al substrate. This was attributed to the effective coating provided by the corresponding manufacturers. However, beyond 250°C, other suppliers of non-stick cookware (PTFE Manuf-2, Granite Manuf-5, PTFE Manuf-3, and Granite Manuf-6) exhibited very weak bonding of C-F atoms and silane compound over a substrate.

Hot Corrosion results and discussion:

Table S7. Hot corrosion results of PTFE Manuf-1 and Granite Manuf-4 non-stick cookware at different temperatures (100, 175, 250, and 350°C)

Sample Made	Operating Temperature, ºC	No of cycles	Net weight loss (-) /Gain (+) mg/cm^2	Sample Made	Operating Temperature, ºC	No of cycles	Net weight loss /Gain mg/cm^2
PTFE Manuf-1	100	0	0	Granite Manuf-4	100	0	0
	100	5	-23.688		100	5	-27.511
	100	10	-23.804		100	10	-27.737
	100	15	-23.888		100	15	-28.44
	100	20	-24.124		100	20	-28.515
	100	25	-24.408		100	25	-28.626
	175	0	0		175	0	0
	175	5	-13.835		175	5	-15.031

	175	10	-23.382		175	10	-19.568
	175	15	-29.288		175	15	-30.564
	175	20	-36.004		175	20	-34.146
	175	25	-38.337		175	25	-35.466
	250	0	0		250	0	0
	250	5	-8.764		250	5	-26.386
	250	10	-18.537		250	10	-26.857
	250	15	-17.631		250	15	-29.355
	250	20	-16.017		250	20	-29.697
	250	25	-14.262		250	25	-24.231
	350	0	0		350	0	0
	350	5	-2.608		350	5	-3.528
	350	10	-2.062		350	10	-12.768
	350	15	-2.804		350	15	-12.466
	350	20	-3.537		350	20	-16.471
	350	25	-0.551		350	25	-15.280

Table S8. Hot corrosion results of PTFE Manuf-2 and Granite Manuf-5 non-stick cookware at different temperatures (100, 175, 250, and 350°C)

Sample Made	Operating Temperature, °C	No of cycles	Net weight loss /Gain mg/cm^2	Sample Made	Operating Temperature, °C	No of cycles	Net weight loss /Gain mg/cm^2
PTFE Manuf-2	100	0	0	Granite Manuf-5	100	0	0
	100	5	-26.586		100	5	-34.520
	100	10	-26.675		100	10	-34.955
	100	15	-27.36		100	15	-35.164
	100	20	-28.364		100	20	-35.520
	100	25	-29.746		100	25	-35.906
	175	0	0		175	0	0
	175	5	-19.195		175	5	-12.866
	175	10	-28.097		175	10	-20.688
	175	15	-37.537		175	15	-26.280
	175	20	-39.920		175	20	-28.177
	175	25	-44.342		175	25	-28.195
	250	0	0		250	0	0
	250	5	-2.422		250	5	-15.955
	250	10	-2.600		250	10	-15.377
	250	15	-9.520		250	15	-12.635

	250	20	-9.217		250	20	-15.440
	250	25	--1.733		250	25	-7.164
	350	0	0		350	0	0
	350	5	4.480		350	5	-14.168
	350	10	4.684		350	10	-13.591
	350	15	4.871		350	15	-10.355
	350	20	2.871		350	20	-13.480
	350	25	6.671		350	25	-11.333

Table S9. Hot corrosion results of PTFE Manuf-3 and Granite Manuf-6 non-stick cookware at different temperatures (100, 175, 250, and 350°C)

Sample Made	Operating Temperature, °C	No of cycles	Weight mg/cm2	Sample Made	Operating Temperature, °C	No of cycles	Weight mg/cm^2
PTFE Manuf-3	100	0	0	Granite Manuf-6	100	0	0
	100	5	-8.053		100	5	-39.488
	100	10	-8.253		100	10	-40.831
	100	15	-8.591		100	15	-41.360
	100	20	-8.760		100	20	-42.355
	100	25	-9.266		100	25	-43.426
	175	0	0		175	0	0
	175	5	-27.182		175	5	-40.262
	175	10	-38.168		175	10	-57.884
	175	15	-45.382		175	15	-60.884
	175	20	-46.782		175	20	-65.786
	175	25	-56.088		175	25	-66.813
	250	0	0		250	0	0
	250	5	-16.124		250	5	-13.920
	250	10	-15.577		250	10	-16.142
	250	15	-16.124		250	15	-14.564
	250	20	-16.475		250	20	-16.684
	250	25	-10.377		250	25	-11.035
	350	0	0		350	0	0
	350	5	-10.720		350	5	-8.160
	350	10	-11.417		350	10	-12.128
	350	15	-12.288		350	15	-12.946
	350	20	-11.635		350	20	-13.480
	350	25	-10.573		350	25	-12.720

Optical microstructures of heated samples at 250°C and 350°C:

Fig. S11-Fig S16 shows the optical microstructural changes in the cookware produced by different suppliers after heating under different conditions. These optical microstructural images are also evidenced by the refinement of α-Al grains and increased FeA_3 precipitates with increasing temperatures.

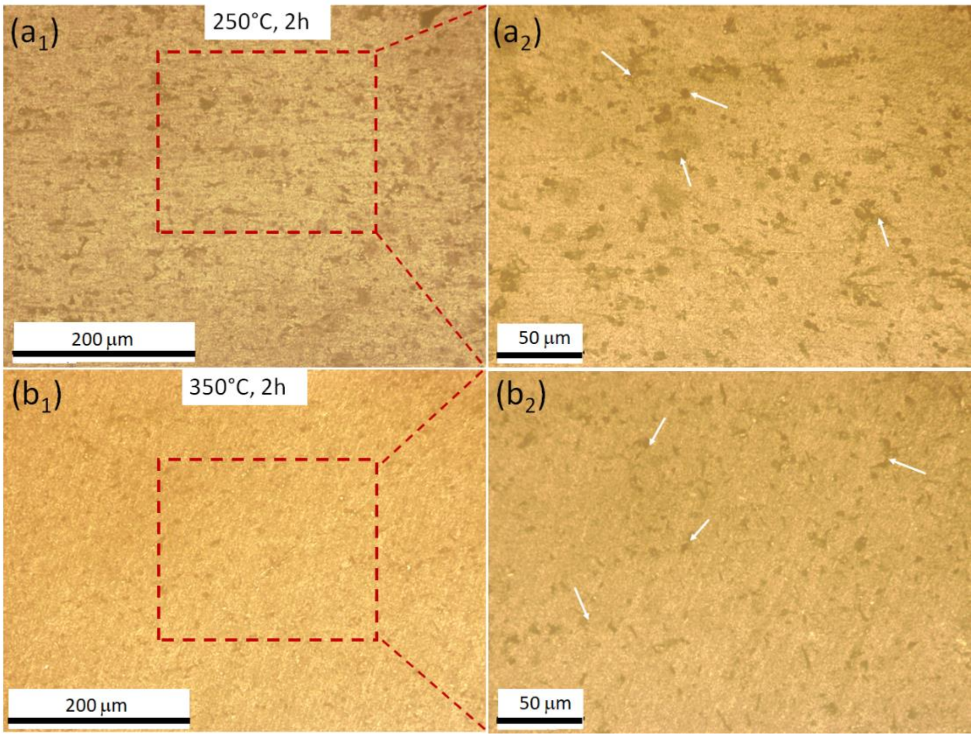

Fig. S11. Optical microstructural images of PTFE Manuf-1 cookware after heating at different temperatures and times: (a_1) & (a_2) 250°C, two hours; (b_1) & (b_2) 350°C, two hours

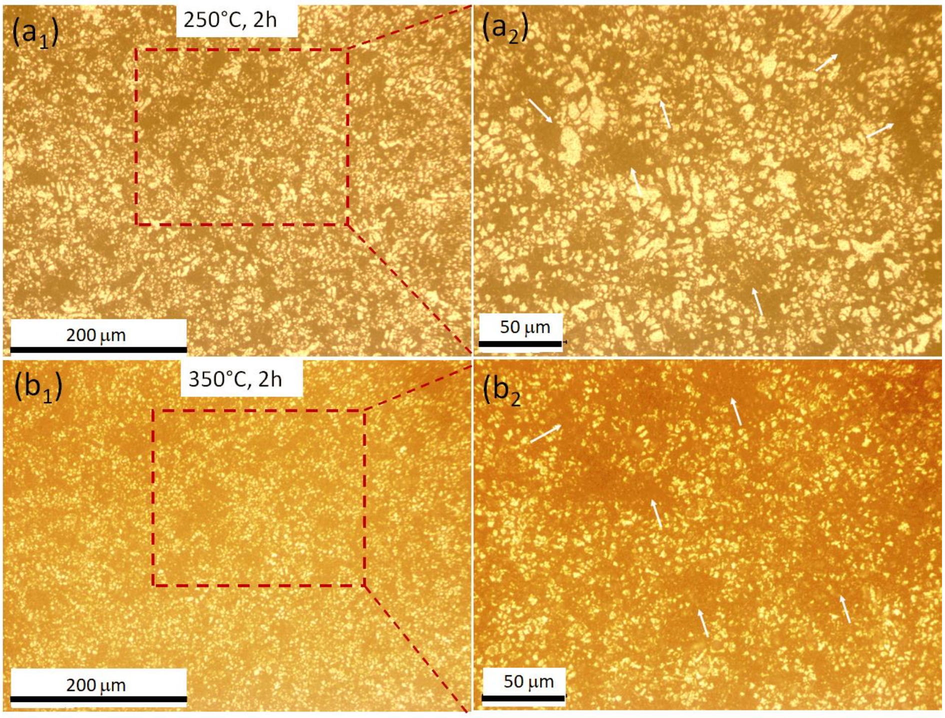

Fig. S12 Optical microstructural images of Granite Manuf-4 ceramic cookware after heating at different temperatures and times: (a_1) & (a_2) 250°C, two hours; (b_1) & (b_2) 350°C, two hours

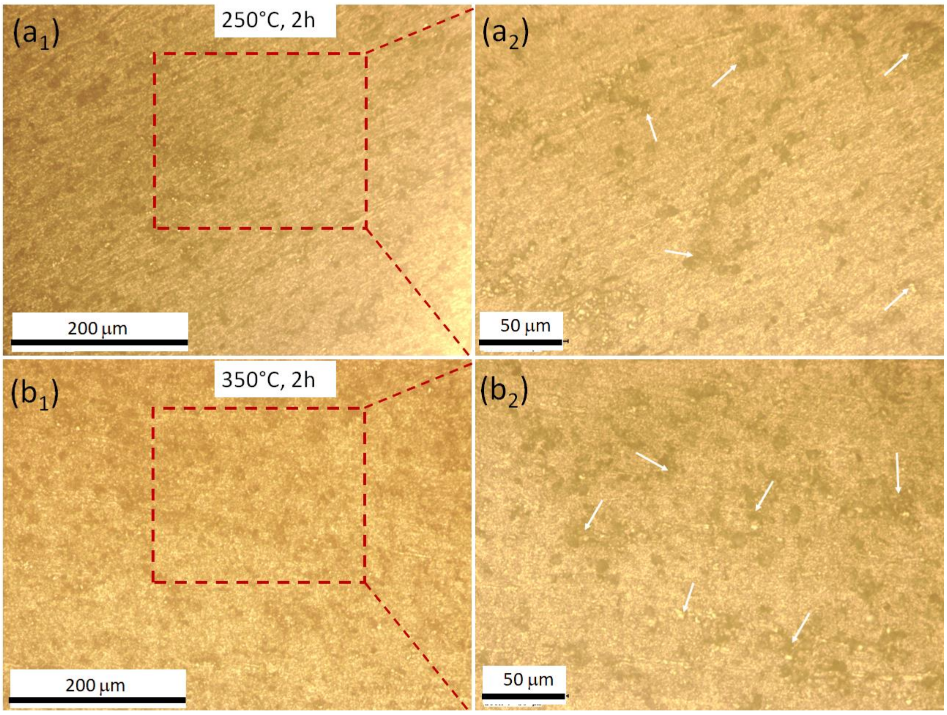

Fig S13 Optical microstructural images of PTFE Manuf-2 cookware after heating at different temperatures and times: (a_1) & (a_2) 250°C, two hours; (b_1) & (b_2) 350°C, two hours

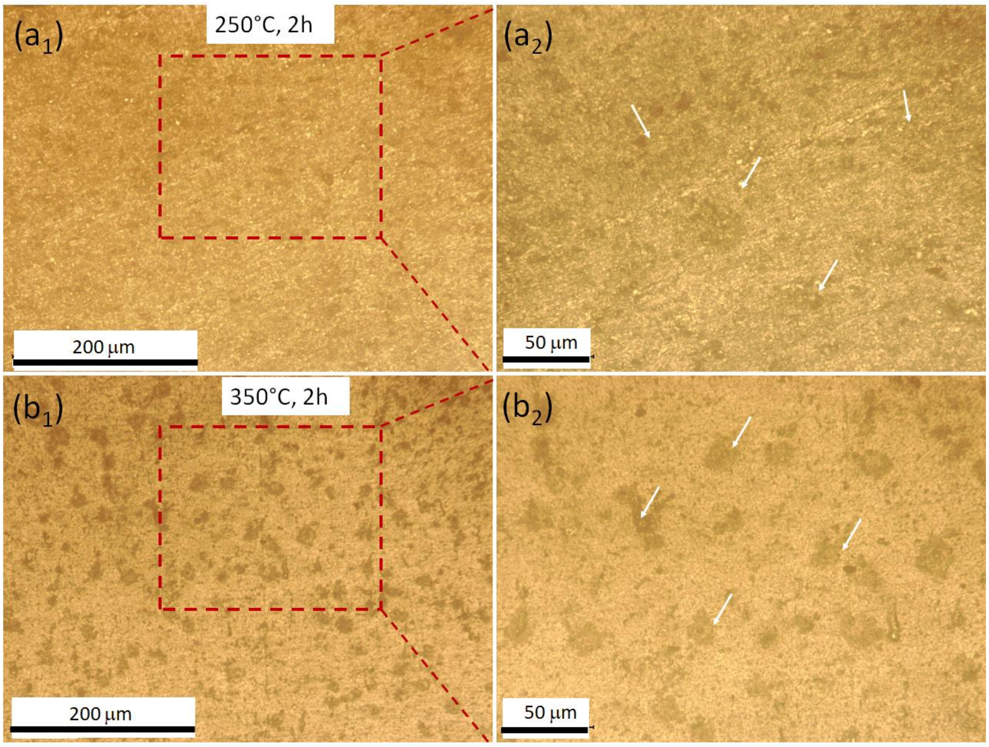

Fig S14 Optical microstructural images of Granite Manuf-5 cookware after heating at different temperatures and times: (a_1) & (a_2) 250°C, two hours; (b_1) & (b_2) 350°C, two hours

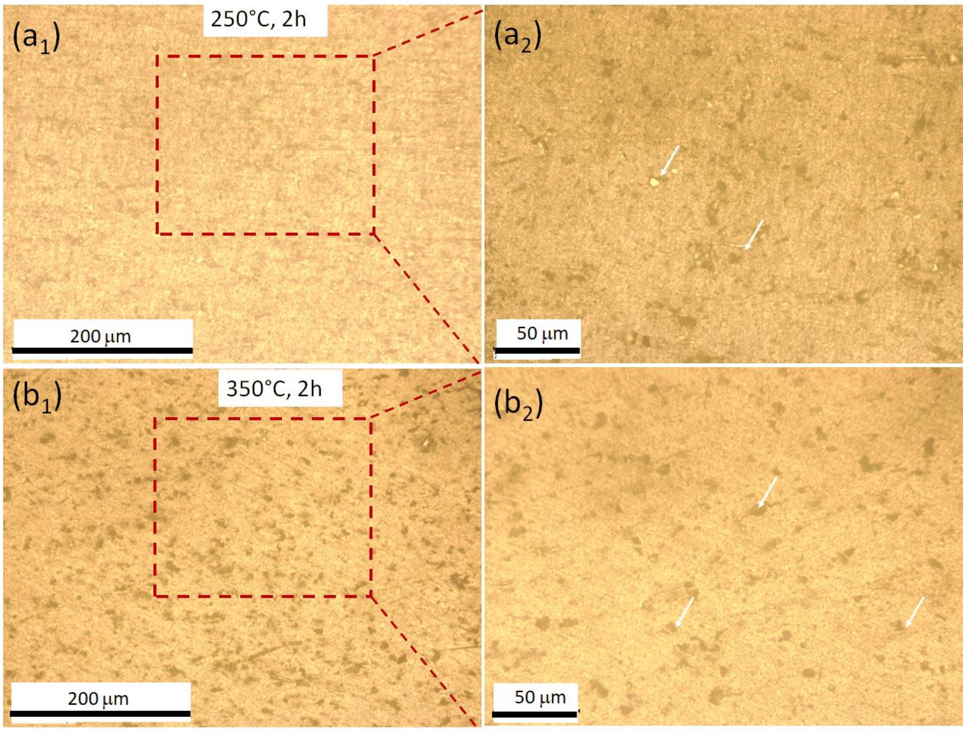

Fig S15 Optical microstructural images of PTFE Manuf-3 cookware after heating at different temperatures and times: (a_1) & (a_2) 250°C, two hours; (b_1) & (b_2) 350°C, two hours

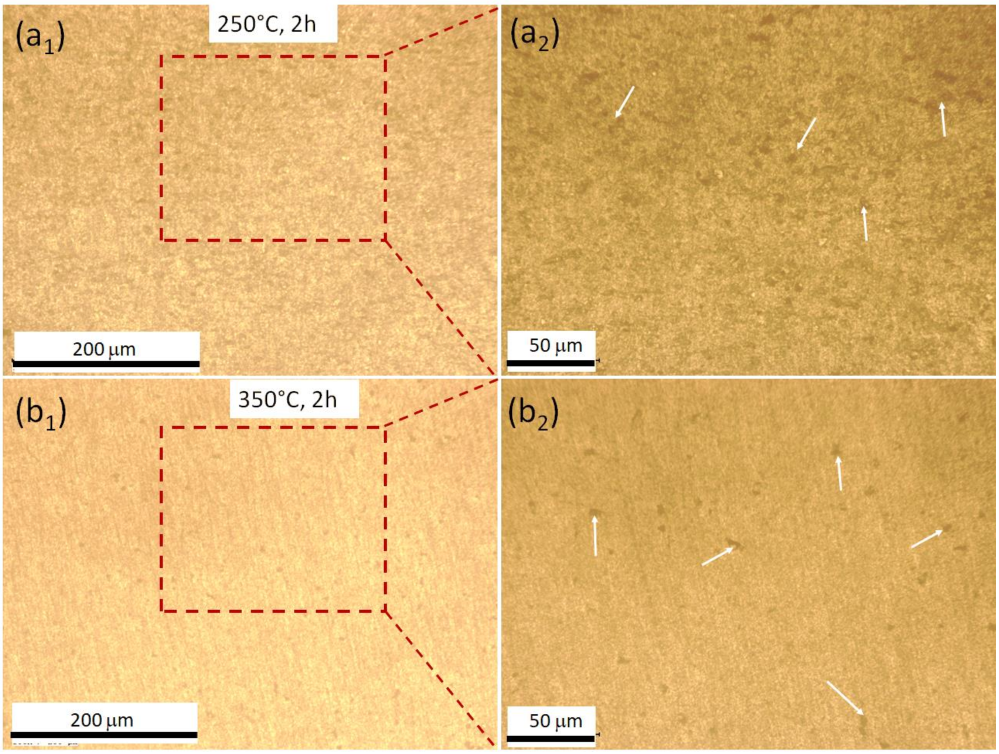

Fig S16. Optical microstructural images of Granite Manuf-6 cookware after heating at different temperatures and times: (a_1) & (a_2) 250°C, two h; (b_1) & (b_2) 350°C, two hours
